# Development of Steady-State and Dynamic Mass and Energy Constrained Neural Networks for Distributed Chemical Systems Using Noisy Transient Data

**DOI:** 10.1021/acs.iecr.4c01429

**Published:** 2024-08-02

**Authors:** Angan Mukherjee, Debangsu Bhattacharyya

**Affiliations:** Department of Chemical and Biomedical Engineering, West Virginia University, Morgantown, West Virginia 26506, United States

## Abstract

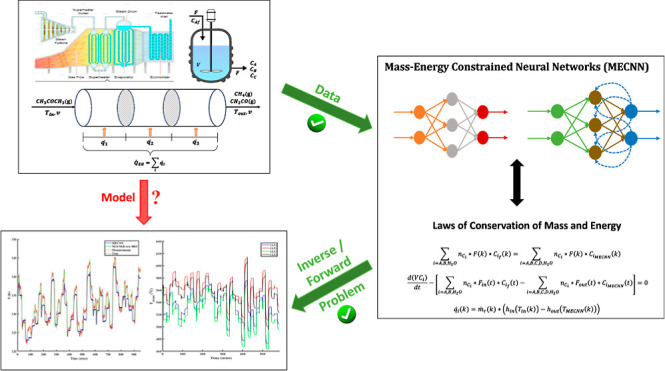

The paper presents the development of algorithms for mass and energy constrained neural network models that can exactly conserve the overall mass and energy of distributed chemical process systems, even though the noisy transient data used for optimal model training violate the same. In contrast to approximately satisfying mass and energy balance constraints of a system by soft penalization of objective function, algorithms have been developed for solving equality-constrained nonlinear optimization problems, thus providing the guarantee of exactly satisfying the system mass and energy conservation laws. For developing dynamic mass-energy constrained network models for distributed systems, hybrid series and parallel dynamic-static neural networks have been leveraged. The developed algorithms for solving both the training and forward problems are validated using both steady-state and dynamic data in the presence of various noise characteristics. The developed data-driven algorithms are flexible to exactly satisfy mass and energy balance constraints for dynamic chemical processes if the system holdup information is available. The proposed network structures and algorithms are applied to the development of data-driven lumped and distributed models of an adiabatic superheater/reheater system, a nonisothermal continuous stirred tank reactor, as well as an electrically heated plug-flow reactor system where one form of energy gets transformed to another. It has been observed that the mass-energy constrained neural networks yield a root mean squared error of <1% with respect to the system truth for the case studies evaluated in this work.

## 1 Introduction

Complex first-principles models are often needed to represent the complicated physics and chemistry associated with many chemical engineering applications. While the first-principles models can exactly satisfy mass and energy conservation, their development often requires considerable time and resources and requires a good understanding of the system physics/chemistry that may not be available or may be difficult to obtain. One alternative to the first-principles models is data-driven models that are relatively easier to develop, simulate, and adapt online for many chemical engineering applications.^1−3^

Recent advances in machine learning (ML) have significantly influenced a wide range of scientific and engineering fields with applications to agricultural sciences,^4^ thermofluidic processes,^5−7^ computational fluid dynamics^8−10^ (CFD), as well as varieties of other complex industrial applications.^11^ More recently, physics-informed neural networks^12^ (PINNs) are being increasingly investigated due to their capability in building models that obey desired physics constraints, with implementations ranging from solving systems of partial differential equations^13−17^ to modeling different chemical processes such as photochemical systems,^18^ hydrofluorocarbon refrigerant mixtures,^19^ biomass pyrolysis process,^20^ heat transfer problems,^11,21^ etc. In the literature cited before, the physics conservation equations have been either incorporated in the objective (loss) function as additional penalty terms to penalize the violation of physics constraint(s) or imposed by imposing the conservation constraints in an additional layer followed by the development of the data-driven model. If the system conservation laws are incorporated in the objective function for training the neural network (NN) models, they act as soft constraints and do not necessarily satisfy physics constraints “exactly”. However, in numerous chemical engineering applications, it is desired that certain mathematical relationships (such as mass and energy balances) are exactly conserved and not only approximated.^5^ In very few recent publications^5,22^ that seek to satisfy the physics constraints exactly, the proposed algorithms are either highly system specific or require a rigorous understanding of the chemical process to impose appropriate physics conservation laws pertaining to the particular system. Another approach for satisfying conservation constraints by NN models for chemical engineering applications^23^ is to send the NN model outputs through a linear transformation block to satisfy mass and energy balances. However, physics conservation laws are not necessarily linear. Furthermore, the corrections applied to the network outputs in the conservation layer, if applied simultaneously while training the network rather than sequentially, can affect the training of the NN model.

It is also desired that the physics constraints be exactly satisfied not only during solving the parameter estimation problem (also interchangeably denoted as training problem) but also during the simulation problem (also interchangeably denoted as forward problem). Another aspect that poses a significant challenge during optimal model synthesis and parameter estimation is developing accurate constrained network models when the training/validation data are noisy.^24^ Uncertainties in data-driven model construction, especially physics-informed networks, can be due to epistemic and/or aleatoric uncertainties because of model form uncertainty, small training data, uncertainty in noise characteristics, parametric uncertainties, etc.^25,26^ The presence of noise in the training data can corrupt the gradient calculations during estimation of network parameters^27^ and can result in biased estimates of parameters during model training. Though variants of PINNs such as Bayesian PINNs,^25^ fractional PINNs,^17^ etc. address the existence of noise in data while solving systems of partial differential equations, algorithms aimed at exactly conserving system physics do not consider the presence of uncertainties in training data at all.^5,22,23^ Furthermore, for most variants of typical PINNs that seek to conserve the mass^5−7,20^ and energy^7,11,21^ of chemical systems, the corresponding parameter estimation algorithms have been set up in a way that the optimization problem only approximately satisfies sets of differential and/or algebraic mass/energy balance equations posed as additional penalty terms in the loss function during training and, hence, may not exactly satisfy the conservation laws especially during solving the forward problem. If constraint violation is merely included as a penalty in the loss function, then even if that penalty term is zero during the training problem, the resulting model may not conserve the laws of system physics exactly when subjected to input conditions during the forward problem (model simulation) that are different from those considered during training. In this work, we seek to develop algorithms that can exactly conserve system physics, particularly mass and energy balance constraints, during both training and forward problems. Our recent work^28^ has shown that fully data-driven mass-constrained neural network (MCNN) models can be successfully implemented for steady-state and dynamic modeling of chemical process systems using noisy transient data. Both training and simulation (forward problem) algorithms had been developed for MCNNs, where the mass (elemental atom) balance equations had been imposed as equality constraints during parameter estimation to ensure that mass conservation laws are exactly satisfied.

Similar to mass conservation, exactly satisfying energy balance constraints becomes critical during construction of accurate data-driven models for many chemical engineering processes, especially energy systems. However, it becomes considerably challenging for a fully data-driven model to exactly guarantee the conservation of energy for most chemical systems due to insufficient quantitative information on the generation/depletion of energy within a system or transformation of energy from one form to another. Contrary to mass balance, the distributed nature and variability of energy constraints, specifically stemming from energy addition/removal at different spatial locations as well as loss or generation of heat due to complicated mechanisms including endothermic/exothermic reactions within the system, make it difficult to develop accurate data-driven models by using only available input/output boundary conditions and limited local measurements. Furthermore, it is typically not obvious what specific constraints should be imposed during parameter estimation to ensure the exact conservation of energy based on different modes of heat transfer without requiring a rigorous understanding of the underlying system. Although a few papers exist until date focusing on exactly conserving the mass of a system for data-driven models,^5,6^ there is currently no work in the existing literature focused on exactly conserving the energy of distributed chemical process systems. This work seeks to develop algorithms for both training and simulation of data-driven models for exactly satisfying mass and energy conservation constraints. It is desired that during training, mass and energy constraints are still exactly satisfied even if noisy data that violate mass/energy constraints are used for training. It should be noted that in the development of data-driven models of many chemical engineering systems for satisfying physics constraints, the training data are typically generated from a first-principles model (and therefore noise-free) or by choosing specific data from high throughput experimental studies^29^ that satisfy mass/energy balances as determined by comparing with a first-principles model. It is also desired that a fully data-driven dynamic model be developed that can satisfy physics constraints, especially mass and energy constraints. This is particularly challenging because mass/energy conservation constraints can be “exactly” applied only to the steady-state data especially if the system is distributed and there is no local measurement available for system holdup. To the best of our knowledge, existing papers^5^ focusing on “exactly” conserving mass/energy balances consider steady-state NNs and obviously steady-state data. It should also be noted that one key difference between satisfying mass constraints and energy constraints is that unlike most mass (atom) balance equations, energy/enthalpy conservation constraints are typically nonlinear and are likely to make the training problem comparatively more challenging. In this paper, we extend the algorithms developed for MCNNs by incorporating additional enthalpy (energy) conservation constraints to ensure the exact conservation of both mass and energy balances by using noisy transient data while solving the training and forward problems. Since we are primarily focused on exactly conserving the mass and energy of the chemical process system, we denote the corresponding NN models as mass-energy constrained neural networks (MECNNs). The proposed structures and algorithms for MECNNs have been applied to develop lumped and distributed models of adiabatic superheater/reheater systems, a nonisothermal stirred tank reactor where heat is generated within the system due to an exothermic reaction, as well as an electrically heated distributed reactor system where one form of energy (such as electric energy) is converted to another (e.g., heat energy). These examples represent many typical chemical engineering systems in which energy conservation is desired to be satisfied by the data-driven model.

In summary, this work addresses the following questions for the development of data-driven NN models:

(1) How can algorithms be developed for guaranteeing the exact conservation of mass and energy during both training and forward (simulation/validation) problems?
(2) What constraints need to be imposed to ensure that mass and energy conservations are exactly satisfied for any distributed chemical process systems involving different modes of heat and mass transfers?
(3) What data are needed for satisfying mass and energy constraints for distributed systems? How do noise characteristics in the data affect the model development?
(4) How can training data that include both steady-state and dynamic data be utilized for model development? How do the training algorithms vary and how do the data-driven model structure and its performance differ based on whether holdup measurement/information is available or not?
(5) How can the sequential parameter estimation algorithms developed in our previous works^2,28^ be leveraged to solve equality-constrained training problems for exactly satisfying mass and energy conservation?

## 2 MECNN Architectures and Error Characterization

This section provides brief descriptions of different candidate NN models considered in this work for developing steady-state and dynamic MECNNs as well as the error (noise/bias) characterizations that were taken into consideration for the training data. Further details about various network architectures can be found in refs (30–34). Since the neural network models are data-driven models, it has been ensured that the model inputs and outputs are measurable by using the current measurement technology and that the desired mass and energy conservation constraints are formulated by using the measured variables. For example, in one of the case studies (discussed later in Section 4) involving a reactive system, the input and output variables include the concentration and temperature measurements of the feed and product streams, respectively, and these variables are used to formulate the corresponding mass (atom) and energy (enthalpy) balance constraints.

### 2.1 Steady-State MECNN Architecture

**Figure 1:**
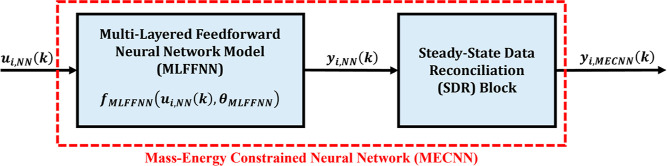
General architecture of steady-state mass energy-constrained neural networks (MECNNs).

A block-oriented structure is considered for steady-state MECNNs, as shown in Figure 1, where a typical multilayered feedforward nonlinear static NN model (MLFFNN) is connected in series with a steady-state data reconciliation (SDR) block. The final outputs from the *i*th neuron of the MECNN are denoted by ***y***_*i*,MECNN_, whereas ***y***_*i*,NN_ represents the intermediate outputs obtained from the *i*th neuron of the fully connected MLFFNN model consisting of parameters (weights and biases) collectively referred to as θ_MLFFNN_. The input variable to the *i*th neuron of the MLFFNN model is denoted by ***u***_*i*,NN_, while *k* represents the indices of steady-state data points. It is to be noted that the selection of the MLFFNN architecture for developing steady-state MECNNs is completely arbitrary since the proposed approaches can be applied irrespective of the choice of network architectures.

The mass and energy conservation laws are imposed as equality constraints as part of the SDR block during both training and forward problems to ensure the exact satisfaction of such constraints. The SDR block also provides the additional degrees of freedom necessary for solving the equality-constrained parameter estimation problem, thus facilitating the simultaneous estimation of parameters for both the MLFFNN and SDR blocks. The proposed architecture is also flexible to accommodate any linear or nonlinear close-form transformation equations that may be used to ensure constraint satisfaction by substituting the SDR block, which requires solving an optimization problem.

The MLFFNN is a typical fully connected feedforward NN including bias. The simplest version of an MLFFNN leads to a three-layered network structure with one input layer, one hidden layer, and one output layer, which can be mathematically represented^2,30^ as
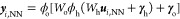
1where *W*_h_ and *W*_o_ denote the connection weight matrices between the input and hidden layers and that between the hidden and output layers, respectively, γ_h_ refers to the bias vector for the hidden layer, γ_o_ represents the bias vector for the output layer, and ϕ_h_(·) and ϕ_o_(·) signify the activation functions of the nodes in the hidden and output layers of the MLFFNN, respectively. In Figure 1, *W*_h_, *W*_o_, γ_h_, and γ_o_ have been collectively referred to together as θ_MLFFNN_.

### 2.2 Dynamic MECNN Architectures

Although typical feedforward NNs feature sufficiently satisfactory performance while modeling various steady-state systems, it may be significantly challenging for static networks to accurately represent highly complex nonlinear temporal systems with time-lagged input variables.^35^ One of our previous papers^2^ has shown that hybrid series and parallel all-nonlinear static-dynamic network models exhibit superior performance than many state-of-the-art recurrent neural networks (RNNs), especially while handling large-sized training data and complex nonlinearities in process transients. The block-oriented structures of the hybrid series and parallel neural network models have been shown in Figure S1 in Supporting Information S.1. While the nonlinear static (NLS) network model in the hybrid structure is represented by an MLFFNN as discussed in Section 2.1, the nonlinear autoregressive model with exogenous inputs (NARX) type of RNN has been considered to represent the nonlinear dynamic (NLD) neural network. Such structures are comparatively compact compared to feedforward networks for similar approximation capabilities while modeling temporal systems due to the presence of feedback connections which eradicate the necessity of data windows for time-lagged inputs.^31^ For all implementation examples in this work, it is assumed that the NARX-type RNN model has zero input delay and one output delay. The resulting RNN model thus obtained can be mathematically expressed in terms of discrete time input–output recursive equations as in ref (31)
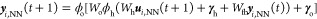
2where ***y***_*i*,NN_(*t* + 1) refers to the predicted output from the NARX-type RNN at time (*t* + 1), *W*_fb_ refers to the connection weights corresponding to the feedback of the model outputs from the previous time step, and the other variables/functions have the exact same interpretation as eq 1. Moreover, similar to the MLFFNN, *W*_h_, *W*_o_, *W*_fb_, γ_h_, and γ_o_ for the NARX-type RNN model have been collectively referred to together as θ_NARX_ for the rest of the paper.

Furthermore, for many systems, holdup information/measurements may or may not be available. For example, the holdup information is typically not available for most distributed industrial processes since it is challenging to account for mass/energy holdup at every spatial location for chemical systems with spatial variations. Therefore, the algorithms for developing accurate dynamic MECNNs may differ based on whether holdup measurements are available or not. Since both the hybrid parallel and series network models can be considered during the development of dynamic MECNNs, two different configurations are possible while taking advantage of the hybrid NN architectures, namely, the hybrid parallel MECNN and the hybrid series MECNN, respectively.

#### 2.2.1 Hybrid Parallel MECNN Structure

**Figure 2:**
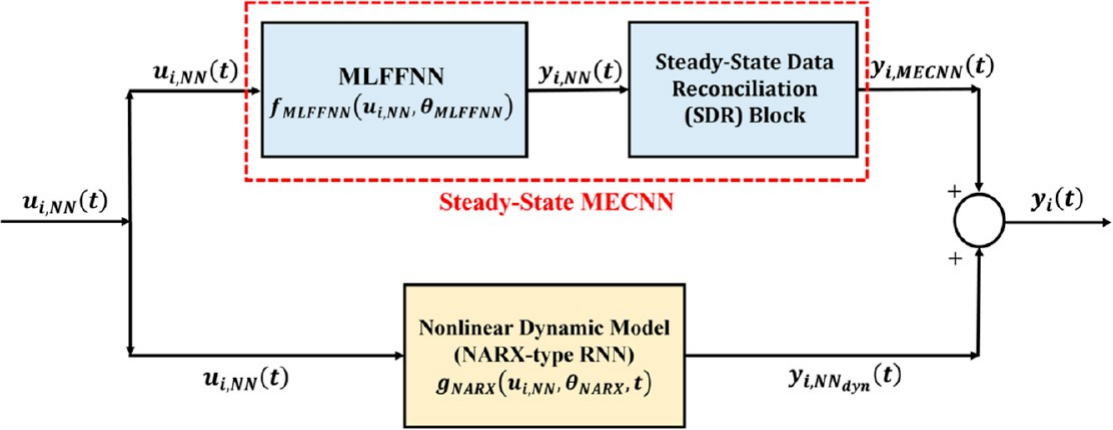
Block-oriented architecture of hybrid parallel MECNN.

The hybrid parallel MECNN structure is proposed especially for systems where holdup information is not available, and hence the mass and energy balance constraints can only be exactly satisfied under steady-state operations. In this approach, it has also been assumed that the overall transient (time-series) data can be distinctly partitioned into dynamic and steady-state zones. Drawing motivation from the hybrid all-nonlinear parallel static-dynamic neural network architecture (refer to Figure S1c in Supporting Information S.1) as proposed in a previous paper by the same authors^2^ and under the assumption of distinctive data partitioning, the architecture of the hybrid parallel MECNN is given by Figure 2. In the proposed architecture, the nonlinear static MLFFNN model coupled with the SDR block together forms the MECNN model, which is connected in parallel with a deterministic nonlinear dynamic neural network model, represented by NARX-type RNN, specifically aimed at predicting the deviations of the dynamic time-series data from the corresponding steady-states. ***y***_*i*_(*t*) denotes the overall outputs from the hybrid parallel MECNN structure, whereas  refers to the outputs from the deterministic dynamic deviation model (NARX-type RNN). It should be noted that Figure 2 is a generic representation of the considered architecture and does not necessarily indicate that the same inputs must be used for the MLFFNN and NARX-type RNN. In Figure 2, *u*_*i*,NN_(*t*) simply represents the union of all inputs that are used as inputs to the MLFFNN and NARX-type RNN.

#### 2.2.2 Hybrid Series MECNN Structures

**Figure 3:**
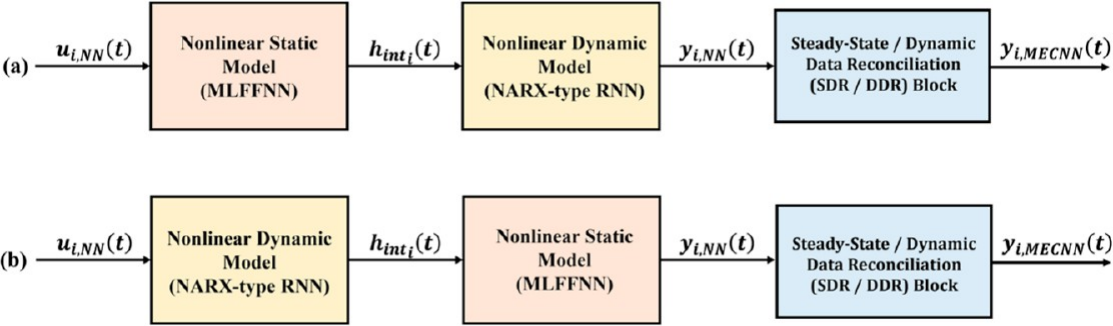
Block-oriented architectures of hybrid series [(a) NLS–NLD, (b) NLD–NLS] MECNNs.

Unlike the hybrid parallel MECNN model, the hybrid series MECNN structures can utilize the entire data during model development without necessarily partitioning them into steady-state and dynamic zones and hence are applicable irrespective of the availability of holdup information. While the unavailability of holdup measurements still requires that the mass and energy balance constraints be imposed only on steady-state zones through an SDR block, the availability of holdup information will facilitate the same constraints to be applied at every transient data point with the SDR block replaced with a dynamic data reconciliation (DDR)-based transformation.^28^ Obviously, in such cases, it is redundant to separately consider the steady-state constraint equations to be imposed in addition to the dynamic constraints. Since two different types of hybrid all-nonlinear static-dynamic networks^2^ are possible while connecting the NLS network (MLFFNN) and the NLD network (NARX-type RNN) in series with each other (i.e., MLFFNN followed by NARX-type RNN referred to as the NLS–NLD model and the reverse configuration denoted as the NLD–NLS model), the hybrid series MECNN can also take up two possible architectures as shown in Figure 3 while modeling dynamic process systems. As before, ***y***_*i*,MECNN_(*t*) represents the final outputs from the overall hybrid series MECNNs, whereas ***y***_*i*,NN_(*t*) denotes the outputs obtained from the hybrid series all-nonlinear static-dynamic neural network models.

Our typical observation from the implementation of hybrid series and parallel all-nonlinear static-dynamic network models for modeling nonlinear dynamic systems is that the overall computational expense associated with each hybrid all-nonlinear model varies from system to system. However, typically, the hybrid models along with their respective sequential training algorithms have shown relatively faster convergence as compared to many existing state-of-the-art models/approaches such as long short-term memory (LSTM), gated recurrent units (GRU), etc., while modeling different transient systems. The key reason for the computational differences between the hybrid series and parallel models is due to their differences in architecture and sequential parameter estimation algorithms presented in our previous publication.^2^

### 2.3 Noise Characterizations

Measurement data considered for developing optimal accurate data-driven models are often noisy. The algorithms and structures proposed in this work have been developed/evaluated by not only considering the absence of noise but also under consideration of two additional types of error (noise/bias) characterizations during both training and forward problems. The primary goal is to assess how the presence or absence of noise and its characterization impact the performance of the proposed MECNNs and their capabilities to precisely conserve the mass and energy of a system. Therefore, the following three types of noise characterizations have been considered during model development and simulation for both steady-state and dynamic implementations of MECNNs.

#### 2.3.1 No Noise

Under consideration of no uncertainties in measurements, the target training data (***y***_*i*,tar_) for MECNNs can be given by
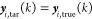
3where ***y***_*i*,true_(*k*) refers to the true data mainly generated from a simulation. Even when trained with the true data that conserve mass and energy, it is highly unlike that, in the absence of mass and energy balance constraints, the outputs from a NN model will exactly satisfy the mass and energy balance constraints unless there is a “perfect match” compared to ***y***_*i*,true_(*k*).

#### 2.3.2 Constant (Time-Invariant) Bias with Gaussian Noise

In this case, the noise in measurement data is characterized by a constant (time-invariant) bias (β_*i*_) in addition to the Gaussian noise (η(0,σ^2^)) with zero mean and known variance (σ^2^), as given by
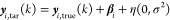
4

#### 2.3.3 Random (Time-Varying) Bias with Gaussian Noise

Here, the measurement noise is characterized by a random (time-varying) bias (β_*i*_(*k*)) in addition to the Gaussian noise of zero mean (η(0,σ^2^)), as given by
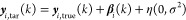
5

It is to be noted that eqs 3–5 remain exactly the same while considering transient time-series data for developing dynamic MECNNs, with the only exception being *k* (indices for steady-state data) being replaced by *t* (time steps for dynamic data).

## 3 Training and Forward Problems for MECNN

### 3.1 Training Problem Formulation for Steady-State Modeling

The steady-state MECNN model considers only the nonlinear static MLFFNN structure representing the NN model, as shown in Figure 1, followed by an SDR block to ensure the exact satisfaction of the mass and energy balance constraints. Moreover, since the SDR transformation considered in this case does not yield any close-form system of equations and itself involves solving an optimization problem, the training problem for steady-state modeling using MECNNs combines optimization problems representing the regression of MECNN model parameters as well as reconciliation of the neural network outputs as given by eq 6a. Therefore, in such approaches, not only the MLFFNN model parameters (referred to as θ_MLFFNN_) are estimated but also the outputs from the MLFFNN model (***y***_*i*,NN_) are reconciled simultaneously.
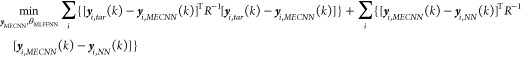
6as.t.
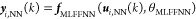
6b
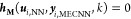
6c
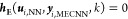
6d

The equality constraints denoted by eqs 6b–6d imposed as part of the constrained training problem (optimization) formulation as shown in eq 6a can be classified into three categories. While eq 6b represents the set of nonlinear static MLFFNN model equations (involving activation functions, network parameters, etc.), eqs 6c and 6d denote the mass (***h***_**M**_(·)) and energy (***h***_**E**_(·)) balance constraints, respectively, corresponding to the SDR block, which can be posed as a function of only input/output data without necessarily requiring a rigorous understanding of the underlying chemical process. However, the specific forms of such mass and energy balance constraint equations may vary with respect to the system under consideration as well as the type of measurements available for a particular system, which will be discussed for each case study considered in this paper in Section 4. The equality-constrained optimization problem is solved using the Interior Point OPTimization^36^ (IPOPT) solver available in the OPTimization Interface (OPTI) toolbox in MATLAB (R2021a) as well as using the algebraic modeling language Pyomo^37^ (v6.7.1) in Python since we found that many commercial Deep Learning/Machine Learning packages available in various software platforms such as in MATLAB (R2021a), TensorFlow (v2.14.0), PyTorch (v2.2.2), or NeuroMANCER^38^ (v1.4.2) are not flexible to incorporate equality constraints (given by eqs 6b–d).

The equality-constrained parameter estimation problem as shown in eq 6a has been formulated based on a “minimum bias” criterion, i.e., for the first two types of error characterizations (eqs 3 and 4), when no noise/bias or a constant (time-invariant) bias with/without Gaussian noise are added to the true data (***y***_*i*,true_) to generate noisy measurements (***y***_*i*,tar_), solution of eq 6a would lead to minimum bias, given the ANN structure/activation functions and training data. However, for the third type of noise characterization (eq 5) considered in MECNN development, i.e., when the measurements are corrupted with a random (time-varying) bias, the parameter estimation problem given by eq 6a would have a lack of identifiability. To address this issue, we propose several plausible additional noise models to be incorporated in the training problem while addressing random (time-varying) bias in measurement data. The different parametric model forms considered and compared in this paper, for specifically modeling the random bias in the training data, consist of linear and quadratic noise (error) models. It is to be mentioned here that the choice of the parametric form of the error model is arbitrary and not specific to either the system or the magnitude of bias/noise that the measured data is corrupted with. Any other model forms for noise can readily be considered and incorporated in the proposed algorithms if and when necessary. In this context, we also assume that the measurement uncertainty (noise/error) model is a function of the overall outputs of the MECNN, i.e., ***y***_*i*,MECNN_ instead of the target (training) measured data (***y***_*i*,tar_) because the uncertainties in measurements are assumed to be a function of the system truth and our notion of true values of model output variables is represented by ***y***_*i*,MECNN_. It should be noted, though, in the absence of such an error model, the model outputs, i.e., ***y***_*i*,MECNN_ will still exactly satisfy the mass and energy conservation equations, albeit with likely higher errors when compared to the true data. The training problem for steady-state modeling when a linear noise model is considered is given by eq 7a, where ***l***_*i*_ denotes the parameters (decision variables) corresponding to the linear noise model (y_*i*,n_*), and *R*_1_, *R*_2_ are weightage terms representing measurement uncertainties. When the quadratic noise model is considered, eq 7b is appropriately reformulated.
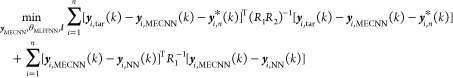
7as.t.
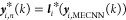
7b
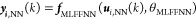
7c
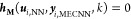
7d
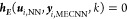
7e

For all case studies considered in this work, the linear and quadratic noise models have been compared based on analyzing the minimum value of the corrected Akaike Information Criteria^39^ (AIC_c_) as given by eq 8, where  is the log-likelihood estimate evaluated at the maximum likelihood parameter values, , *k* is the number of parameters characteristic of the overall MECNN model, and *n* is the total sample size. The objective of this approach to select the optimal noise (error) model is to penalize overfitting during model training and reduce the model complexity. The AIC_c_incorporates a bias correction term due to small sample sizes assuming that the estimated parameters are normally distributed around the true parameter values.^40^
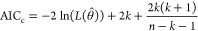
8

### 3.2 Forward Problem Formulation for the Steady-State Model

**Figure 4:**
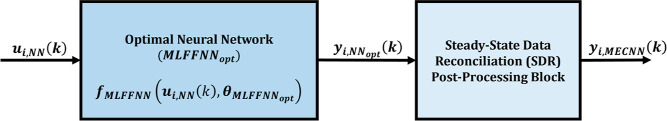
General architecture for forward problem formulation for MECNNs.

Although the MLFFNN model parameters (weights and biases) can be regressed to guarantee the exact satisfaction of mass and energy balance constraints for all data on which the MECNN is trained, it can still not be ensured that the optimal MLFFNN will continue to exactly conserve mass and energy of a system for forward problems (i.e., model simulation) when subjected to unknown inputs different from those used during the training problem. Therefore, for the forward problem as well, the SDR block is included following the optimal MLFFNN model, as seen in Figure 4, to ensure the exact conservation of system mass and energy during the forward problem. With the SDR block in place, the forward problem evolves from being a straightforward validation task into an equality-constrained optimization problem described in eq 9a. The formulation of the forward problem, however, remains consistent, irrespective of the type of error characterization considered in this work.
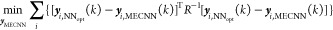
9as.t.
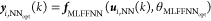
9b
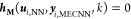
9c
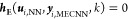
9d

### 3.3 Training Problem Formulations for Dynamic Modeling

The training problem formulation for dynamic MECNNs differs based on whether parallel or series network architecture shown in Figures 2 and 3, respectively, is considered.

#### 3.3.1 Training Problem for Hybrid Parallel MECNN

The training problem for the hybrid parallel MECNN is formulated under the assumption that the entire time-series data can be distinctly partitioned into steady-state and dynamic zones. While several approaches have been developed in the literature for partitioning steady-state and dynamic data,^41−44^ in this work, we simply characterize the steady-state zones (referred to as *t*_s_) as continuous time intervals within the overall transient training data where the maximum deviation from the mean value does not exceed ±0.5%. It should be noted that the algorithm developed in this work can be applied regardless of the specific algorithm used for partitioning the data. The partitioning of training data facilitates the flexibility of solving a sequential optimization^2^ problem for the overall training problem of the hybrid parallel MECNN. In such an approach, the MECNN block (as represented by the red dashed box in Figure 2) is first developed based on only the steady-state zones, i.e., the formulation of the training problem remains exactly same as given by eqs 6a and 7a with *k* being replaced by *t*_s_, followed by training of a deterministic nonlinear dynamic deviation model represented by a NARX-type RNN by using the residuals/deviations obtained with respect to the training data and steady-state MECNN model outputs. The deviation data thus generated serve as the training data for the NARX-type RNN model independently during the sequential approach while minimizing the typical squared error objective function as given by eq 10a. The detailed algorithm for solving the training problem of hybrid parallel MECNN involving eqs 6a and 10a can be found in Figure S2 in Supporting Information S.1. Obviously, when the training data is considered to be contaminated with the presence of time-varying bias, it will require eq 6a being appropriately replaced by eq 7a by incorporating the parametric model form of the error model as an additional constraint.
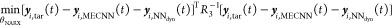
10as.t.
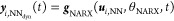
10b

#### 3.3.2 Training Problem for Hybrid Series MECNNs

In contrast to the hybrid parallel MECNN, the hybrid series MECNNs are flexible to accommodate the entire time-series dynamic data, regardless of whether there is access to system holdup information or not. When holdup measurements are not available, then the mass and energy conservation constraints can only be applied to the steady-state data (*t*_*s*_) while formulating the SDR block in the training problem due to insufficient information about the system holdup during transients, thus leading to eq 11a where **φ**_**NLS**–**NLD**_ represents the set of neural network model equations for the hybrid series NLS–NLD type of static-dynamic network. Although the hybrid all-nonlinear series static-dynamic network structure^2^ may have two different possible configurations, i.e., NLS–NLD and NLD–NLS, we only show here the formulation of the NLS–NLD type of hybrid series network, given by eq 11a, for the training problem of hybrid series MECNN in the absence of any holdup information. However, it is to be noted that the training problem formulation involving the NLD–NLS type network model will be exactly the same, with the only exception of replacing **φ**_**NLS**–**NLD**_ by **φ**_**NLD**–**NLS**_ in eq 11b, i.e., the hybrid model equations for NLD–NLS type of series static-dynamic NN structure.
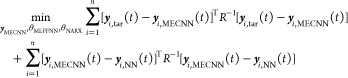
11as.t.
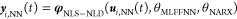
11b
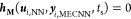
11c
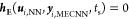
11d

For the case in which the system holdup information is available during formulation of the training problem for hybrid series MECNNs, the approach discussed above can readily be extended to exactly satisfy the mass (***h***_**M**_(·)) and energy (***h***_**E**_(·)) balance constraints at each time step in the transient profile. Holdup measurements may be available for systems with no or negligible spatial distribution, such as a continuous stirred tank reactor (CSTR). The corresponding training problem for hybrid series (NLS–NLD) MECNN in the presence of holdup measurements can be given by
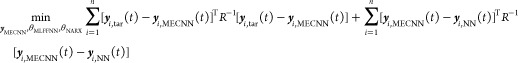
12as.t.
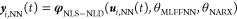
12b
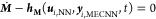
12c
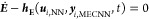
12dwhere ***M*˙** and ***E*˙** denote the mass and energy holdup/accumulation terms, respectively, within the system at a given time step *t*.

However, unlike the parallel hybrid MECNN training algorithm, it may lead to excessive computational expense to simultaneously estimate the optimal parameters for both the nonlinear static and dynamic neural network models in a monolithic approach along with satisfying the equality constraints imposed within the SDR/DDR block in the series architecture as shown in Figure 3. Therefore, the sequential training algorithms that have been developed as part of our previous work^2,28^ can be leveraged since those have been shown to perform significantly superior to the simultaneous approach and provide flexibility of solving the NLS and NLD models independently using same/different training algorithms irrespective of their arrangement. Such decomposition-based approaches implemented for training different networks in a hybrid framework or even evaluating model parameters for different layers within a single complex deep neural network can ensure faster computation by decomposing the overall model structure and utilizing existing deep learning packages if necessary. Furthermore, similar to steady-state modeling, the training problem formulations discussed in eqs 11a and 12a based on the availability of system holdup measurements can be slightly modified to incorporate the noise model (linear, quadratic, etc.) as additional constraints when the measured data are corrupted with a time-varying bias. As before, the choice of the specific model form for the noise models is arbitrary and not dependent on the systems under consideration. Typically, the parameter estimation algorithm proposed for hybrid series MECNN models shows higher computational expense as compared to that for hybrid parallel MECNNs because it incorporates the entire time-series data in the equality-constrained parameter estimation problem, unlike the latter that partitions the data into steady-state and dynamic zones, followed by solving the equality-constrained problem only for the steady-state zones, which form a much smaller subset of the entire transient data set.

Furthermore, since the proposed MECNNs are fully data-driven models developed assuming the availability of only input/output boundary conditions and thermo-physical properties, and assuming that a rigorous understanding of the heat transfer mechanisms of the underlying system is not available, then sufficient information (measurements) on the accumulation of mass and energy within the system needs to be available if it is desired that the proposed approaches exactly satisfy mass and energy conservation even during transients. The lack of hold-up measurements for any given system does not necessarily mean that the proposed MECNN models cannot be used for those systems. It simply implies that the mass and energy conservation laws can only be applied at steady-state during solving the training problem for dynamic MECNNs.

### 3.4 Forward Problem Formulations for the Dynamic Model

For both hybrid parallel and series MECNNs, the corresponding forward problems follow a sequence of algorithms similar to the respective training problems. The forward problem for the hybrid parallel MECNN model involves the exact same simulation algorithm of steady-state MECNNs (as described in eq 9a) involving the SDR postprocessing block to generate steady-state outputs that are added to those obtained from the optimal NARX-type RNN deviation model yielding the final time-series dynamic outputs. On the contrary, for the hybrid series MECNN, the forward problem considers that the outputs obtained from the optimal series (NLS–NLD or NLD–NLS) all-nonlinear static-dynamic network models are postprocessed through an SDR block when the holdup information is not available or DDR block when the holdup information is available for exactly satisfying mass and energy conservation. The formulation of the forward problem for a particular type of hybrid series or parallel MECNN, however, remains consistent under all types of noise characterizations considered in this work.

## 4 Case Studies and Corresponding Mass–Energy Balance Constraints

The proposed MECNN architectures and training/simulation algorithms discussed above are evaluated for various nonlinear chemical process systems. While for most chemical processes, the quantification of mass conservation is unambiguous and straightforward, quantifying the overall energy balance of the system may be relatively challenging in a fully data-driven approach due to different possible sources of energy transfers associated with a system including (but not limited to) generation/depletion of heat as well as energy transformation from one form to the other within the system. Therefore, in this work, three nonlinear processes with different levels of complexity for mass and energy transfer within the system have been chosen for modeling. While the first case study considers the development of both lumped and distributed models of an adiabatic superheater/reheater system^45,46^ involving no generation/loss/conversion of energy within the system, the second and third case studies, respectively, consider developing MECNNs to model the widely used nonisothermal lumped (i.e., CSTR) van de Vusse reactor system^47,48^ involving an exothermic reaction (hence heat generation), as well as an electrically heated distributed tubular (plug-flow) reactor^48,49^ involving vapor-phase cracking of acetone during the production of acetic anhydride, where electric energy gets transformed to heat to facilitate the endothermic reaction. Although, the mass balance constraints are trivially satisfied for the superheater/reheater system (Case Study 1), both mass and energy conservation laws have been simultaneously incorporated as equality constraints for the other two case studies considered in this work. In this work, the mass conservation (expressed as elemental atom balance equations) and energy (or enthalpy) conservation constraints have been posed using only a subset of input/output measurements, as well as operational parameters and thermo-physical properties for each case study. It is to be noted that the proposed structures and algorithms are generic and can readily be extended while considering additional physics/chemistry-based constraints depending on the systems under consideration.

For all case studies considered in this work, the training and validation (simulation) data have been generated from respective first-principles models subject to step-changes in model input before contaminating them with different types of noise/bias characterizations. It is also assumed that we have access to measurements for all of the input and output variables necessary to ensure mass and energy conservation. These input and output variables are normalized using min–max normalization, and 60% of the entire data set is allocated for training the MECNNs, while the remaining 40% is used for model simulation (validation). We perform k-fold cross-validation (with *k* = 5) for all case studies. Both the standard logistic sigmoid and hyperbolic tangent activation functions have been used in the neural network models considered in this work. Even though the NNs are trained with respect to the normalized values of the input and output variables, it is ensured that the mass and energy balance constraints involve the actual absolute values of the variables concerned. For steady-state modeling, the initial guess for the MECNN parameters is determined by training a standalone MLFFNN without any mass balance constraints (i.e., without the DDR block) irrespective of the noise characterization. All neural network models considered in this work are structured as fully connected single hidden layered networks, with the number of neurons in the input and output layers exactly equal to the number of input and output variables, respectively.

### 4.1 Case Study 1: Adiabatic Superheater/Reheater System

The development of accurate data-driven models for superheater/reheater systems poses to be significantly challenging, due to their inherent nonlinearities and complexity. This work focuses on developing both lumped and distributed data-driven models of a superheater/reheater system, under the following assumptions:

- The process is adiabatic, i.e., no heat depletion/loss/generation within the system.
- No transformation of energy takes place from one form to the other (such as due to resistive heating, viscous heat dissipation, etc.) within the system.
- No change of phase occurs among the different species involved in the system.

**Figure 5:**
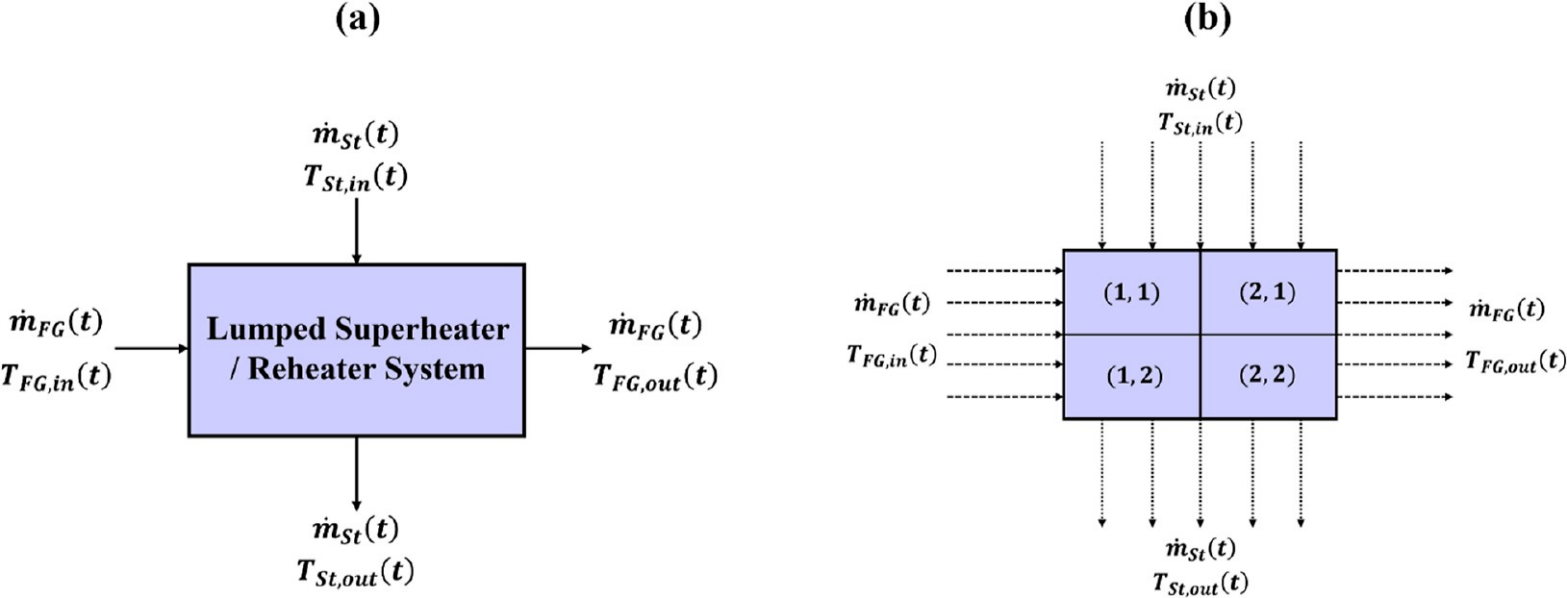
Schematic of the (a) lumped and (b) distributed superheater/reheater model for ECNN implementation.

A schematic of the lumped parameter model for the superheater/reheater system is shown in Figure 5a, where flue gas and steam flow in a cross-flow arrangement. Since the overall mass of both steam and flue gas are trivially conserved in such a system, the mass flow rates of the two streams  are considered to be exactly the same at the inlet and outlet of the superheater/reheater system. No spatial variation is considered for steam and flue gas flows while developing the lumped parameter model. For both lumped and distributed models, the input variables of the MECNN are represented by the inlet mass flow rates, inlet temperatures (*T*_St,in_,*T*_FG,in_), and pressures (*P*_St_,*P*_FG_) of steam and flue gas streams, whereas the outlet temperatures (*T*_St,out_,*T*_FG,out_) of both streams have been considered as model outputs. Furthermore, since only energy conservation laws need to be incorporated for this case study, the optimal MECNNs thus obtained will be simply referred to as energy-constrained neural networks (ECNNs). The energy balance constraints (***h***_**E**_(·)) imposed during the formulation of both training and forward problems for the lumped parameter ECNN model can be expressed as steady-state enthalpy balance equations involving the input and output boundary conditions, as given in eq 13. The specific heat capacity of the flue gas stream (*C*_pFG_) is assumed to be a constant value calculated from ideal gas laws, whereas the specific enthalpies of steam (*h*_St,in_,*h*_St,out_) have been calculated using functions defined in the IAPWS R7-97 formulation for the properties of water and steam in terms of steam inlet/outlet temperature and pressure. It should be noted that the algorithm is agnostic to the specific formulation of the energy conservation constraint.
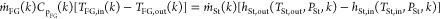
13

Though the lumped ECNN model ensures that the energy conservation laws are exactly satisfied at the boundary conditions during both training and forward problems, no information can be derived regarding the distribution of temperature within the superheater/reheater system. Therefore, the proposed algorithms for developing the lumped parameter model can be easily extended to construct a distributed model, if needed, by imposing additional constraints on the ECNN model representing the closure of the energy balance for each discrete element of the spatial coordinates. The schematic of the distributed superheater/reheater model for ECNN implementation has been shown in Figure 5b.

For simplicity, the superheater/reheater model, as shown in Figure 5a, has been discretized into two control volumes each in the horizontal and vertical directions, thus leading to a 2 × 2 grid structure as shown in Figure 5b. Obviously, a larger number of discretization grid points may be considered for higher spatial resolution, if needed.^46^ The flow pattern as well as the input and output variables to the ECNN model remain the same for both flue gas and steam as compared to the lumped model. However, for both training and forward problems while developing ECNN for the distributed model, the enthalpy balance constraint eq (eq 13) has to be exactly satisfied for each of the individual control volumes in spatial directions, along with the boundaries of the system. Local measurements of both flue gas and steam temperatures at the intermediate grid points have been considered during the development of optimal ECNNs.

### 4.2 Case Study 2: Non-Isothermal van de Vusse Reactor System

The nonisothermal model of the van de Vusse reactor is one of the most vastly studied multi-input-multioutput (MIMO) processes due to its highly complex nonlinearities.^47^ The reaction involves the production of cyclopentenol (B) from cyclopentadiene (A) by acid-catalyzed electrophilic addition of water in a dilute aqueous solution. The side products of this reaction are cyclopentanediol (C) and dicyclopentadiene (D). Cooling water flows through the jacket surrounding the reactor to remove excess heat generated due to the highly exothermic reaction within the system, as shown in Figure S3 in Supporting Information S.2. The detailed mathematical model consisting of the overall material and energy balance equations and nominal values of the involved parameters can be found in the literature.^50,51^ Furthermore, unlike the first case study, the development of optimal MECNNs for the nonisothermal van de Vusse reactor system requires both mass and energy balance constraints to be imposed at steady-state during training and forward problem formulations.

The feed stream contains predominantly component A, with small quantities of B. The volumetric flow rate (*F*) and inlet temperature (*T*_0_) of the feed, volumetric flow rate (*F*_j_) of the jacketing fluid (cooling water), along with the concentrations of A (*C*_Af_) and B (*C*_Bf_) in the feed stream have been considered as inputs to the MECNN model, whereas the model output variables are represented by the concentration of all reaction species, i.e., A (*C*_A_), B (*C*_B_), C (*C*_C_), and D (*C*_D_), in the product stream and the outlet temperatures of the product stream (*T*) and jacketing fluid (*T*_j_). It is assumed that the inlet and outlet volumetric flow rates are the same across the reactor system as well as across the cooling jacket. Furthermore, since the reaction takes place in an aqueous solution, water (H_2_O) has been considered as one of the species while formulating the mass balance constraints for the training and forward problems. The mass balance constraints in terms of elemental atom (C, H, and O) balance equations as well as the energy balance constraint for this system have been provided in eq 14 through eq 17, where *n*_C_*i*__, *n*_H_i__, and *n*_O_*i*__ represent the number of C, H, and O atoms, respectively, in the *i*th species, ρ_r_ denotes the density of the reactor mixture, *h*_in_ and *h*_out_ refer to the specific enthalpy of the reactant and product mixtures as functions of inlet (*T*_0_) and outlet (*T*_MECNN_) temperatures, respectively, *m*_*j*_, *V*_*j*_, and *C*_p_*j*__ refer to the mass, volume, and specific heat capacity of cooling water flowing through the jacket, and *T*_*j*_0__ represent the inlet temperature of the jacketing fluid (cooling water).
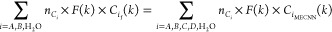
14
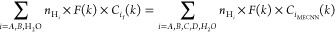
15
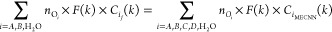
16
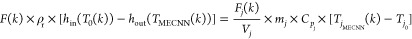
17

Since this case study considers a CSTR system with no spatial distribution, the system holdup information is explicitly modeled using the outlet boundary conditions and instantaneous volume of the reactor. For example, the mass (in terms of C atom) and energy balance constraints imposed at every time step (*t*) for this case study are expressed in terms of inlet/outlet flow rates (*F*_in_,*F*_out_) and instantaneous reactor volume as
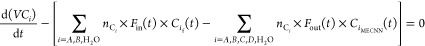
18
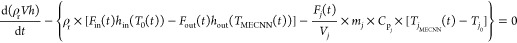
19

### 4.3 Case Study 3: Electrically Heated Tubular Reactor for Cracking of Acetone

The vapor-phase cracking of acetone (A) to yield ketene (B) and methane (C) is considered as one of the key steps in the production of acetic anhydride for manufacturing purposes.^49^ The endothermic reaction is first-order with respect to A and is typically carried out in tubular (plug flow) reactors, as shown in Figure S4 in Supporting Information S.2. The detailed mathematical model with the overall material and energy balance equations and nominal values of the involved parameters can be found in refs (48, 52, and 53). The feed stream to the reactor has been assumed to contain pure acetone in the vapor phase. The volumetric flow rate (*v*) of the reaction mixture remains constant across the distributed tubular reactor. The following variables are considered to be inputs to the MECNN model–concentration of A in the inlet stream (*C*_Ain_), temperature of the inlet stream (*T*_in_), volumetric flow rate of the reaction mixture (*v*), and the heat fluxes  provided by the external electric heater to each discretized element of the tubular reactor in the axial direction, while the outlet concentrations of all reaction species, i.e., A (*C*_A_), B (*C*_B_), and C (*C*_C_) and the temperature (*T*) of the product stream are considered as model outputs. Similar to Case Study 2, both mass and energy (enthalpy) balance constraints have been imposed at steady-state while developing MECNNs for this case study, as well. However, unlike the lumped nonisothermal CSTR system, the tubular reactor is discretized into numerous elements in the axial directions (in this case, three discretization grids have been shown in Figure S4 in Supporting Information S.2) with the mass and energy balance constraints imposed on each discretized control volume in addition to the system boundaries. Elemental atom balance equations for C, H, and O balance as well as the enthalpy balance constraint in terms of input/output boundary conditions and thermo-physical properties of the reaction species have been described in eq 20 through eq 23 for the *i*th control volume, where  refers to the mass flow rate of the reactor mixture, and *h*_in_ and *h*_out_ denote the specific enthalpies expressed as functions of *T*_in_ and *T*_MECNN_, respectively. It is to be noted that since the tubular (plug flow) reactor is a distributed system and the holdup information for this system is assumed to be unavailable, the mass and energy conservation constraints cannot be applied during the transients.
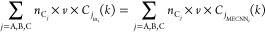
20
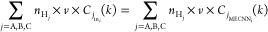
21
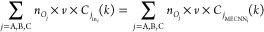
22
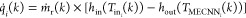
23

## 5 Results and Discussion: Steady-State Modeling

### 5.1 Case Study 1: Adiabatic Superheater/Reheater System

The different types of error characterizations considered in this work for generating measurement data for steady-state modeling of both lumped and distributed adiabatic superheater/reheater systems have been separately analyzed and discussed. For presenting results of this and other case studies (also for dynamic modeling), the data used for training the MECNN/ECNN models are referred to as “measurements”, irrespective of whether they had been generated from a first-principles model or collected from experiments. Furthermore, since mass is trivially conserved for this case study, the performance of ECNNs has been analyzed based on model efficiency in exactly satisfying the energy conservation of the system. The comparison of error in energy balance for all cases of error characterizations considered for the lumped and distributed ECNN models has been evaluated in terms of absolute percentage error (APE) in energy (*Q*) balance defined as
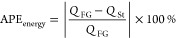
24where *Q*_FG_ and *Q*_St_ refer to the heat flux expressions for flue gas and steam, respectively.

#### 5.1.1 Lumped Parameter Model

The lumped parameter model considers a simple input–output superheater/reheater model with no spatial distribution of the temperature profiles within the system.

##### 5.1.1.1 No Noise

**Figure 6:**
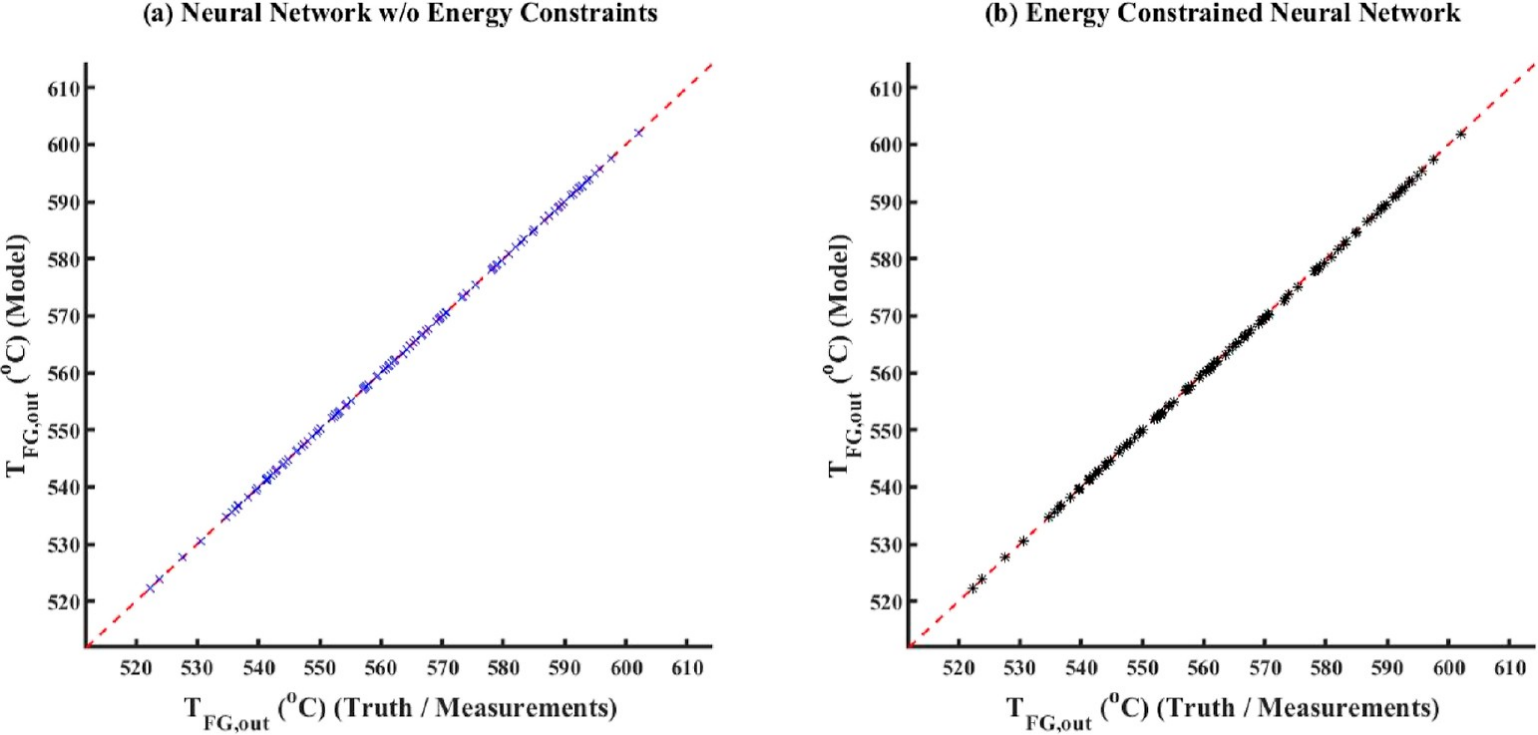
Comparison of results between ECNN and NN w/o energy constraints for the simulation data of *T*_FG,out_ (noise in the measurement data represented by eq 3).

**Figure 7:**
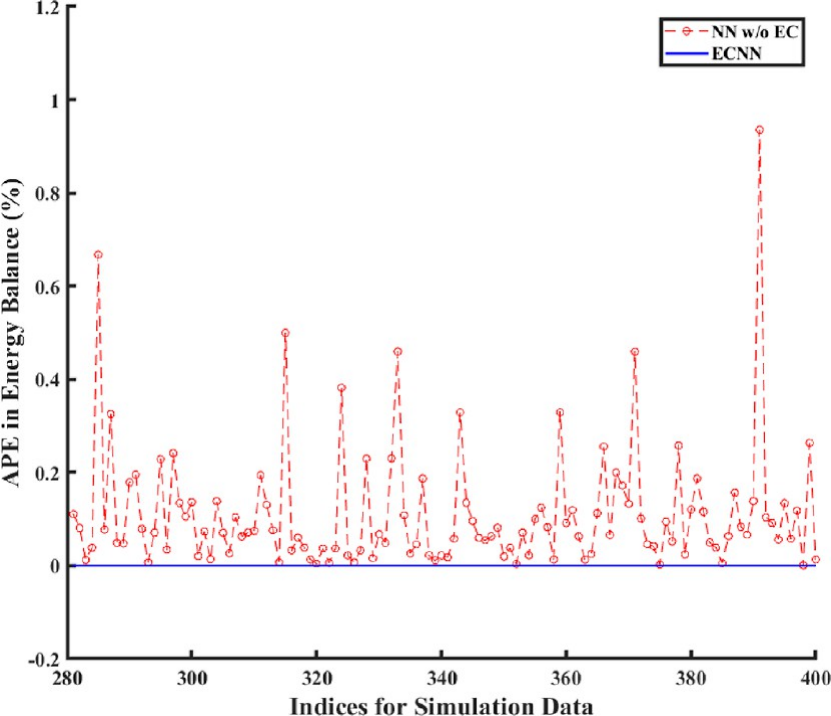
Comparison of error between ECNN and NN w/o energy constraints for energy balance during simulation (noise in the measurement data represented by eq 3).

For the case with no noise added to true data to generate measurements, Figure 6 compares the model simulation results with truth/measurements (here, measurements is the same as true data) between ECNN and NN without (w/o) energy constraints for *T*_FG,out_. Similar parity plots for simulation data of *T*_St,out_ have been shown in Figure S5 in Supporting Information S.3. The corresponding training plots have not been included here for the sake of brevity. From Figure 6, it can be clearly observed that both ECNN and unconstrained NN have a relatively good match with the simulation data irrespective of whether energy conservation constraint is considered, resulting in around 0.04 and 0.07% root mean squared error (RMSE), respectively. However, Figure 7 shows that although the ECNN exactly satisfies the energy balance (in terms of APE in *Q* balance defined in eq 24) for the simulation data, the NN w/o energy constraints leads to relatively larger APEs, the maximum error recorded being almost 1%.

##### 5.1.1.2 Constant Bias with Gaussian Noise

**Figure 8:**
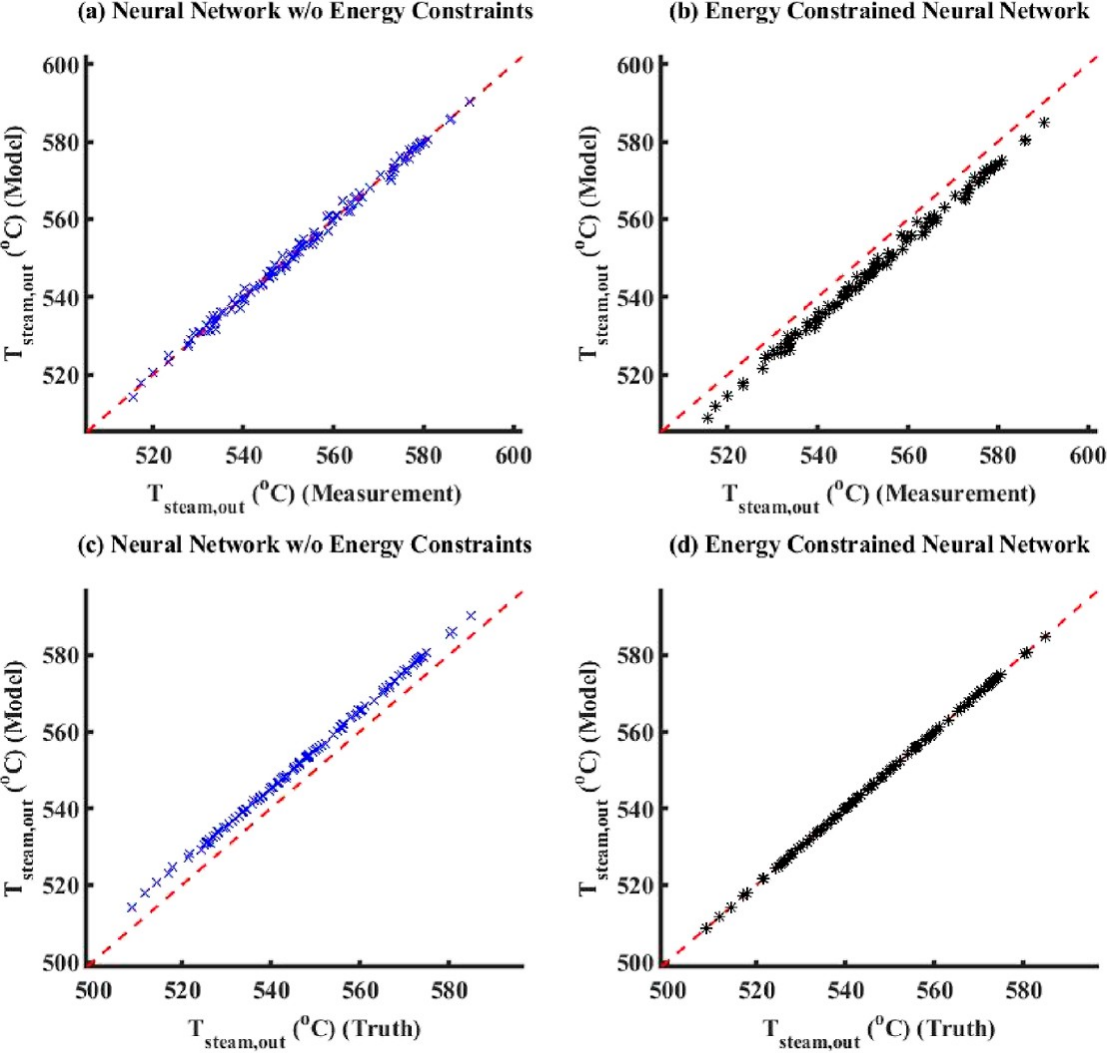
Comparison of results between ECNN and NN w/o energy constraints for the simulation data of *T*_St,out_ (noise in the measurement data represented by eq 4).

For this case study, a constant bias of around 5 °C has been added to the simulated true output temperatures from the first-principles model to generate training data, along with an additional Gaussian noise distribution with μ = 0 and σ^2^ = 1. The parity plots obtained by solving the training problem (training) showing the comparison between ECNN and a standalone unconstrained NN in the absence of any energy balance constraints while modeling *T*_St,out_ is given in Figure S6 in Supporting Information S.3, whereas the corresponding results obtained by solving the forward problem for the same output variable are shown in Figure 8. Similar plots for *T*_FG,out_ have been included under Figures S7 and S8 in Supporting Information S.3.

As expected from Figure 8a, it is evident that the outputs of the standalone neural network (with or without energy constraints) show an excellent match (0.2% RMSE) with the measurements while leading to larger errors (around 1.0% RMSE) with respect to the true data. The ECNN does exactly satisfy energy conservation at steady-state showing an accurate match with the true data with nearly 0.01% RMSE during both training and simulation. A similar observation can be made in Figure S9 in Supporting Information S.3 that shows the APE in energy balance, where the NN w/o energy constraints results in APE as high as 20 and 30% for training and simulation data, respectively, while the ECNN has zero error.

##### 5.1.1.3 Random Bias with Gaussian Noise

**Figure 9:**
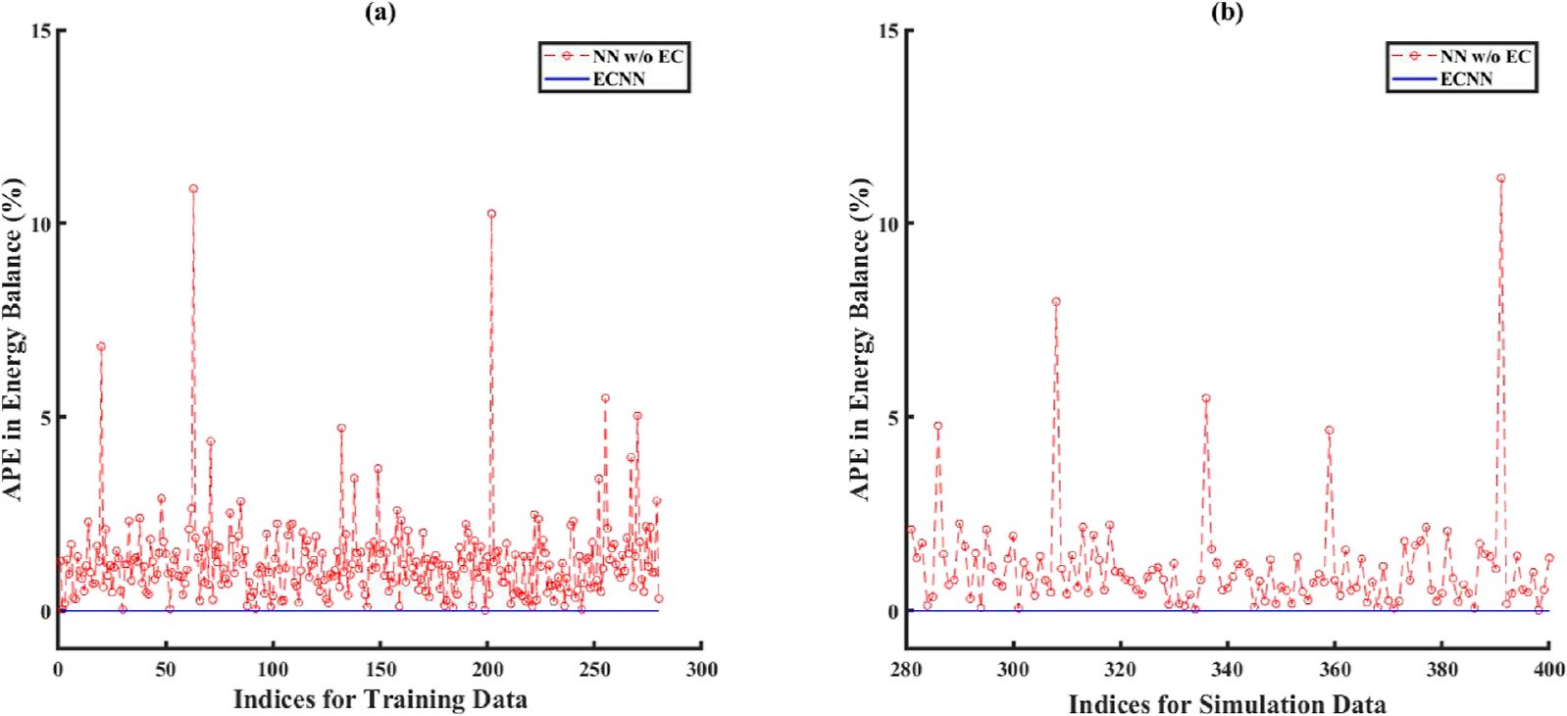
Comparison of error between ECNN and NN w/o energy constraints for energy balance during (a) training and (b) simulation (noise in the measurement data represented by eq 5).

For this case, a random correlated bias selected within the range of −8 °C to +2 °C has been added to the system truth to generate training measurement data, in the presence of an additional Gaussian noise distribution with μ = 0 and σ^2^ = 4. The results have shown characteristics similar to those observed for the other two types of error characterizations considered for this case study. Though the parity plots comparing the performances of ECNN and NN w/o energy constraints for training data have not been included in this paper for brevity, Figures S10 and S11 in Supporting Information S.3 show model results vs truth and model results vs measurements for ECNN and unconstrained NN during forward problem (simulation) when the measurement data are corrupted with a random bias. Furthermore, it is to be noted that the linear form of the error model yielded superior results as compared with the cases when a no-noise model or a quadratic noise model had been used in the training problem for this case study. However, as noted earlier, the choice of the noise model is arbitrary and specific to neither the system nor the magnitude of bias/noise added to the true data to generate training data. As before, the NN w/o energy constraints yielded an excellent match (RMSE value of 0.06%) with respect to the measurements during the forward problem, while showing errors in the range of 0.8–0.9% RMSE (equivalent to around 4–5 °C) when compared to truth. On the contrary, ECNN shows an excellent match with the truth (RMSE of 0.05 and 0.06%, respectively, for outlet temperatures of steam and flue gas) but a high error with respect to measurements (RMSE of around 1% for both steam and flue gas outlet temperatures). Figure 9 shows that the ECNN yields practically negligible APE during both training and simulation, whereas the NN w/o energy constraints shows APE of as high as 11 and 12% for training and simulation data, respectively.

#### 5.1.2 Distributed Model

As discussed before, the development of the distributed model of the superheater/reheater system involves the exact satisfaction of the energy balance constraint equation (as given by eq 13) at each discretized element (control volume) in the spatial coordinates, in addition to the system boundaries. Furthermore, for all types of error characterizations considered in this case study, it is assumed that all local measurements are available for both training and forward problems due to optimal placement of measurement sensors in the system.^54,55^ However, for one specific type of error characterization, i.e., when the measurement data are corrupted with a constant bias in the presence of an additional Gaussian noise distribution, two sensitivity analyses have been performed to describe how the unavailability of some local measurements influences the optimal model development. Obviously, such sensitivity analyses can be easily performed for other types of error characterizations as well as other similar case studies involving local measurements for steady-state/dynamic modeling but are not included here for brevity.

##### 5.1.2.1 No Noise

**Figure 10:**
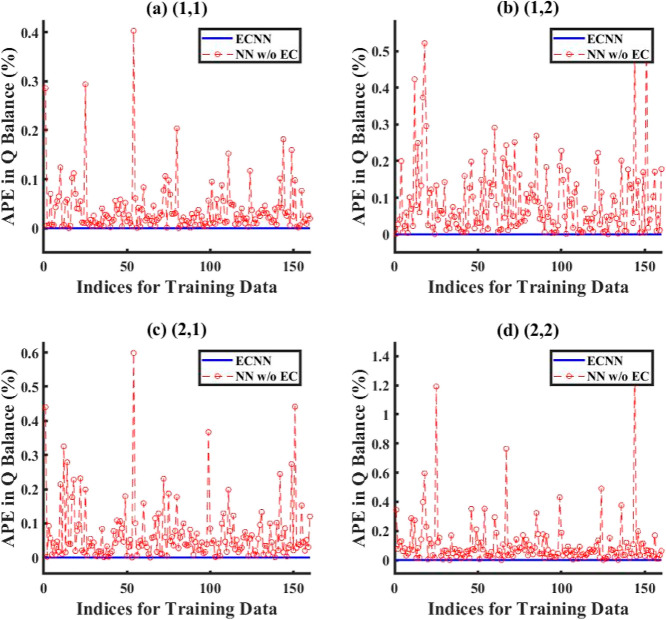
Comparison of error between ECNN and NN w/o energy constraints for energy balance at intermediate grids during training (noise in the measurement data represented by eq 3).

For the case with no noise, Figures S12 and S13 in Supporting Information S.3 compare the model results vs truth/measurements (for this case, true data and measurements are the same) for the ECNN and NN without energy constraints for training data of *T*_FG,out_ and *T*_St,out_, respectively, at the grid (2,2). Similar performance plots at other locations have not been included here for the sake of brevity. It can be observed that both ECNN and NN w/o energy constraints show excellent matches with the training data, with percentage RMSE values of 0.03 and 0.04%, respectively. However, Figures 10 and S14 in Supporting Information S.3 show that while the ECNN exactly satisfies energy (*Q*) balance not only at the system boundaries but also at each discretized grid location for the training data, there is a relatively larger error recorded for the NN w/o energy constraint, showing maximum APE as high as 0.6% at the boundary conditions and 1.4% at one of the grid points.

Figures S15 and S16 in Supporting Information S.3 show the comparison between ECNN and NN w/o energy constraints in terms of APE in energy balance for simulation data at the boundary as well as the individual grid points, respectively. Again, the ECNN satisfies energy balance constraints even during the forward problem, whereas the standalone unconstrained NN model shows relatively higher APE (as high as 0.75% at the boundary and 1.7% at one of the intermediate grids) in *Q* balance. The corresponding parity plots for the forward problem are not included here for brevity. It is to be noted that for all types of error characterizations, the NN w/o energy constraints is basically a collection of individual lumped models (in this case, a collection of 4 individual models, one for each grid), whereas the ECNN is a single model with continuous connections expressed as additional constraints along the discretization elements in the spatial coordinates.

##### 5.1.2.2 Constant Bias with Gaussian Noise

**Figure 11:**
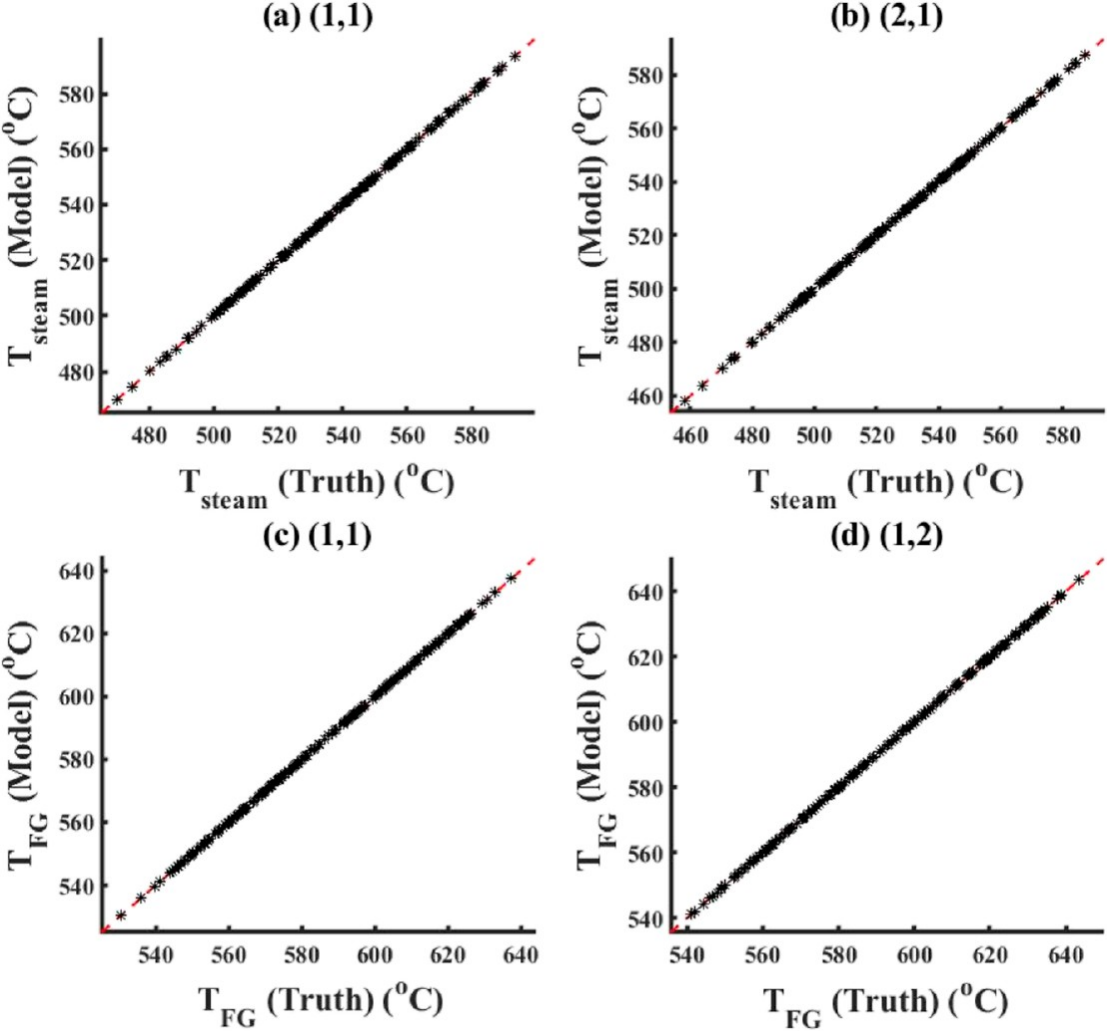
Results from ECNN for the simulation data of *T*_St,out_ and *T*_FG,out_ at intermediate discretization grids (noise in the measurement data represented by eq 4).

Here, a constant bias equal to 5 °C is added to each output variable along with an additional Gaussian noise with μ = 0 and σ^2^ = 0.5. The training results obtained from the distributed ECNN for this case have not been included here for brevity. Figures 11 and S17 in Supporting Information S.3 show the model results vs truth for the ECNN and NN w/o energy constraints, respectively, for simulation data, at the individual discretized grid points within the distributed system.

**Figure 12:**
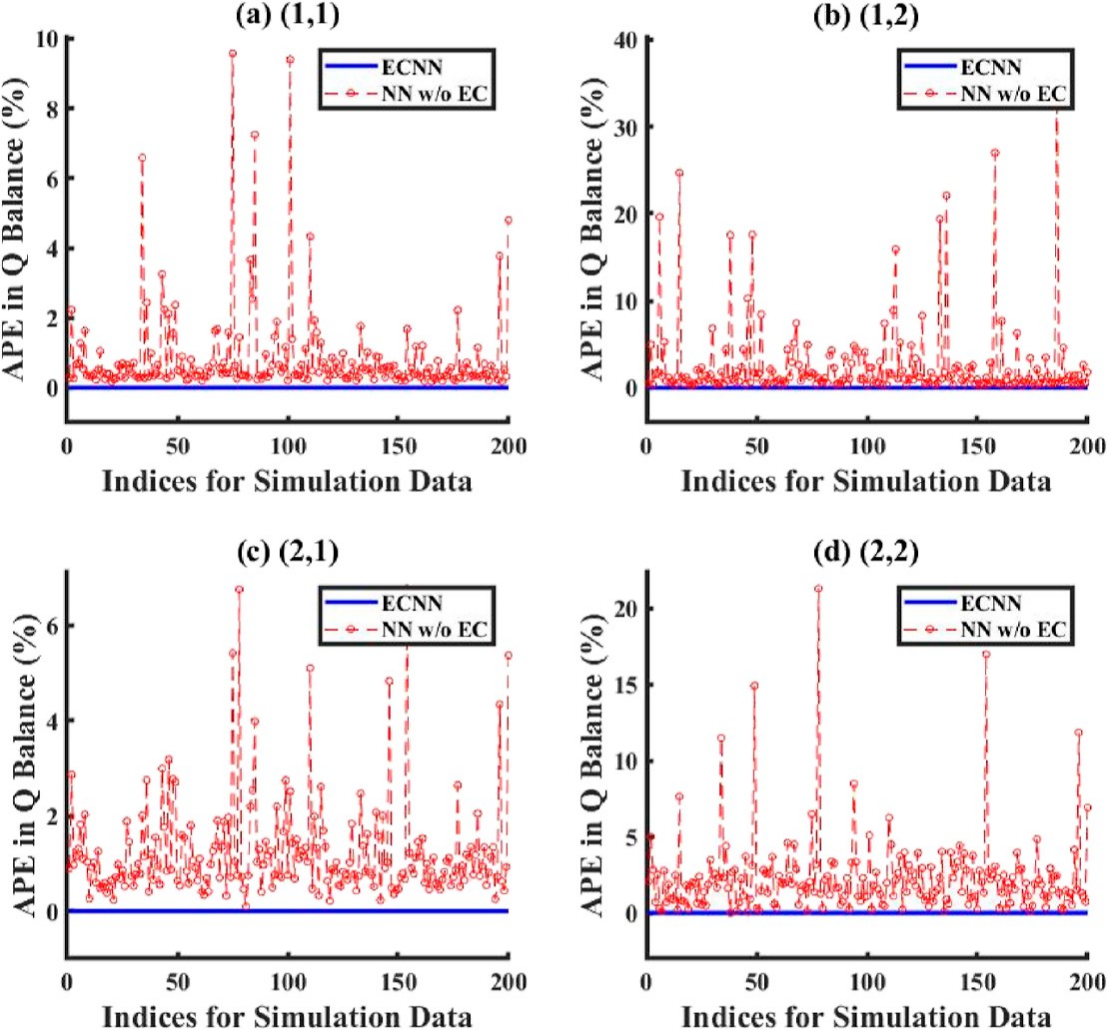
Comparison of error between ECNN and NN w/o energy constraints for energy balance at intermediate grids during simulation (noise in the measurement data represented by eq 4).

While the outlet flue gas and steam temperatures obtained from the NN w/o energy constraints show a significantly higher error (around 0.8% RMSE, which is equivalent to around 5 °C) with respect to the true data on an average, the ECNN output variables show an excellent match with the system truth, recording an average error of around 0.01% RMSE. Furthermore, the ECNN also exactly satisfies energy conservation constraints both at the boundary and intermediate grid points, whereas the unconstrained NN shows maximum APE in energy balance as high as 5.5% at the boundary and 32% at one of the intermediate grids. The comparison of error between ECNN and NN w/o energy constraints for energy balance at the intermediate discretized grid points in the spatial coordinates can be seen in Figure 12, whereas that at the system boundaries has been included under Figure S18 in Supporting Information S.3.

All results discussed so far have been obtained by assuming that all local measurements are available during the construction of optimal data-driven ECNN models due to optimal placement of sensors in the system. However, in many practical applications, it is highly unlikely that all local states will be measured with reasonable accuracy. Therefore, the value/quality incorporated by such local measurements during the development of a distributed data-driven model has been analyzed by performing sensitivity studies. Two types of analyses have been performed where only a fraction of all available local measurements are considered during the optimal model synthesis and model simulation. The first approach is based on “sampling”, where multiple subsets of all available local measurements at intermediate discretization grids are extracted and considered for model development, in addition to all boundary conditions. This approach also assumes that measurements are available at all intermediate locations but not for all steady-state (or dynamic) observation indices as compared to the boundary measurements. On the contrary, the second approach is based on “availability” of sensor (measurement) data at the individual discretization elements in the spatial coordinates. Unlike the sampling approach, this analysis assumes that local measurements are available for all steady-state (or dynamic) indices but not at all locations. For both sensitivity analyses, the minimum % RMSE calculated between the ECNN model outputs and true data among all possible combinations of data selection is given by eq 25, where *k*_*n*_ denotes the size of simulation data, *n* denotes the total number of output variables, and ***C*** denotes the set of all possible combinations for each sensitivity analysis under consideration. It is also to be noted that a finite number of combinations exist for the second approach (i.e., when the local measurements are not available at one or more discretization grids during model development). The results presented below are for the local measurement data for which min % RMSE is obtained for a given availability of % local measurements.
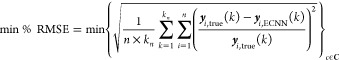
25

**Figure 13:**
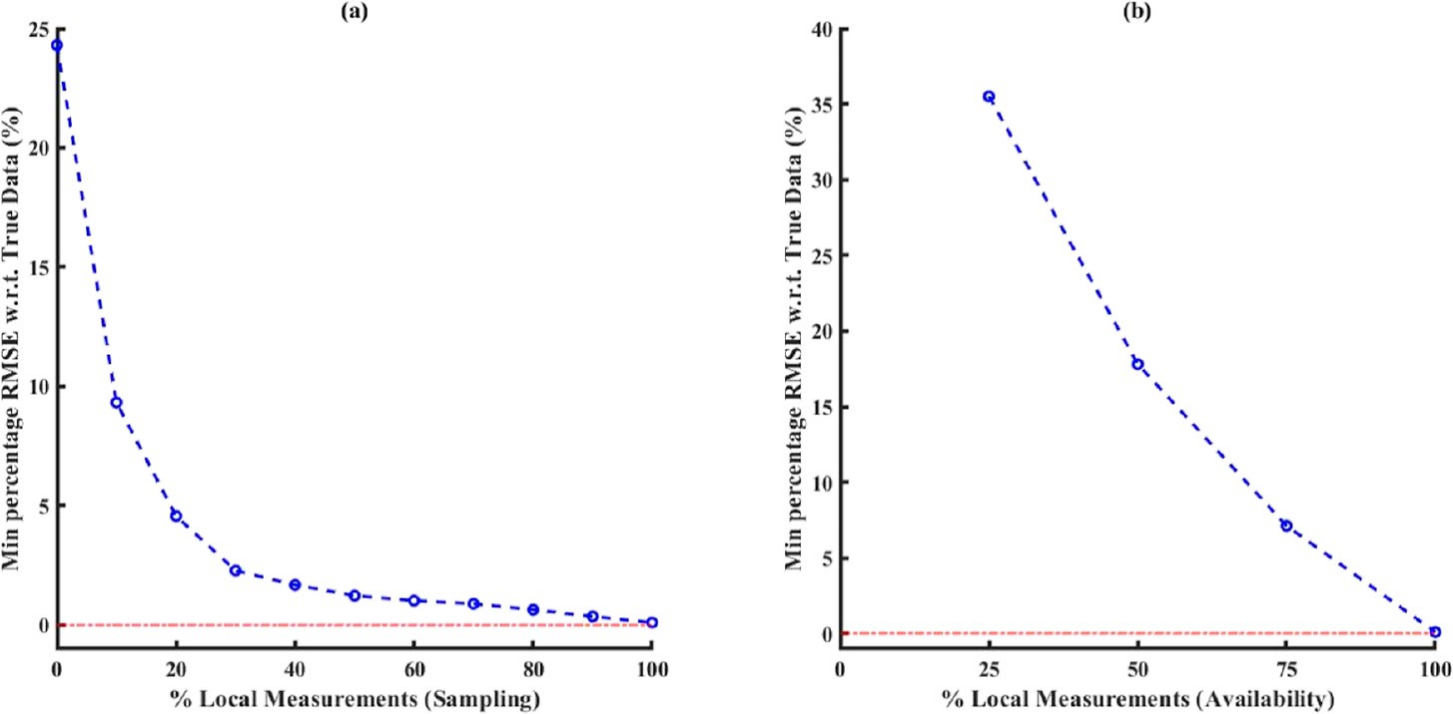
Comparison of minimum % RMSE for simulation data using ECNN from sensitivity analyses based on (a) sampling and (b) availability of local measurements (noise in the measurement data represented by eq 4).

Figure 13 shows the minimum percentage RMSE for simulation data calculated between ECNN outputs and system truth corresponding to the two sensitivity analyses based on availability of local measurements. It can be clearly observed that the minimum RMSE monotonously decreases as more and more local information is made available during optimal model synthesis. Obviously, this type of sensitivity analyses can be performed for the other case studies during both steady-state and dynamic implementations as well under all different types of error characterizations. However, for brevity, the results for all other case studies have been discussed for the case when all local measurements are available during ECNN/MECNN training and simulation (forward problem).

##### 5.1.2.3 Random Bias with Gaussian Noise

For this case, a random bias within the range of −2 to +8 °C is considered along with an additional Gaussian noise with μ = 0 and σ^2^ = 0.5. However, as discussed before, not considering a separate noise (error) model in the training problem may lead to a lack of identifiability, and hence the algorithm may converge at a solution, which although ensures the exact satisfaction of energy balance constraints may not be close to the true data. Without the consideration of any error model, it has been observed that although the results obtained from the ECNN exactly satisfied the energy conservation laws at both boundary as well as intermediate discretization grid points, the predictions of *T*_FG,out_ and *T*_St,out_ from the optimal ECNN model showed average percentage RMSE values of 2.1 and 2.8% during training and simulation, respectively, with respect to the system truth. Corresponding plots have not been included here for brevity. Both the linear and quadratic noise models yielded superior results as compared to the no-noise model case. However, the linear noise model resulted in the minimum value of the corrected AIC_c_ during optimal model synthesis (training) as compared to the quadratic model, thus leading to a lesser percentage RMSE during model simulation, as shown in Table 1.

**Table 1:** Comparison in Average % RMSE Values Calculated with Respect to True Data for ECNN Predictions among Linear vs Quadratic vs No-Noise Model Considered to Address Random Bias in Data

	avg % RMSE between ECNN outputs and true data		
type of noise models in ECNN	training data	simulation data	AIC_c_ during model training
no noise/error model	2.1%	2.8%	2368
linear model	0.08%	0.1%	1615
quadratic model	0.05%	0.4%	1784

**Figure 14:**
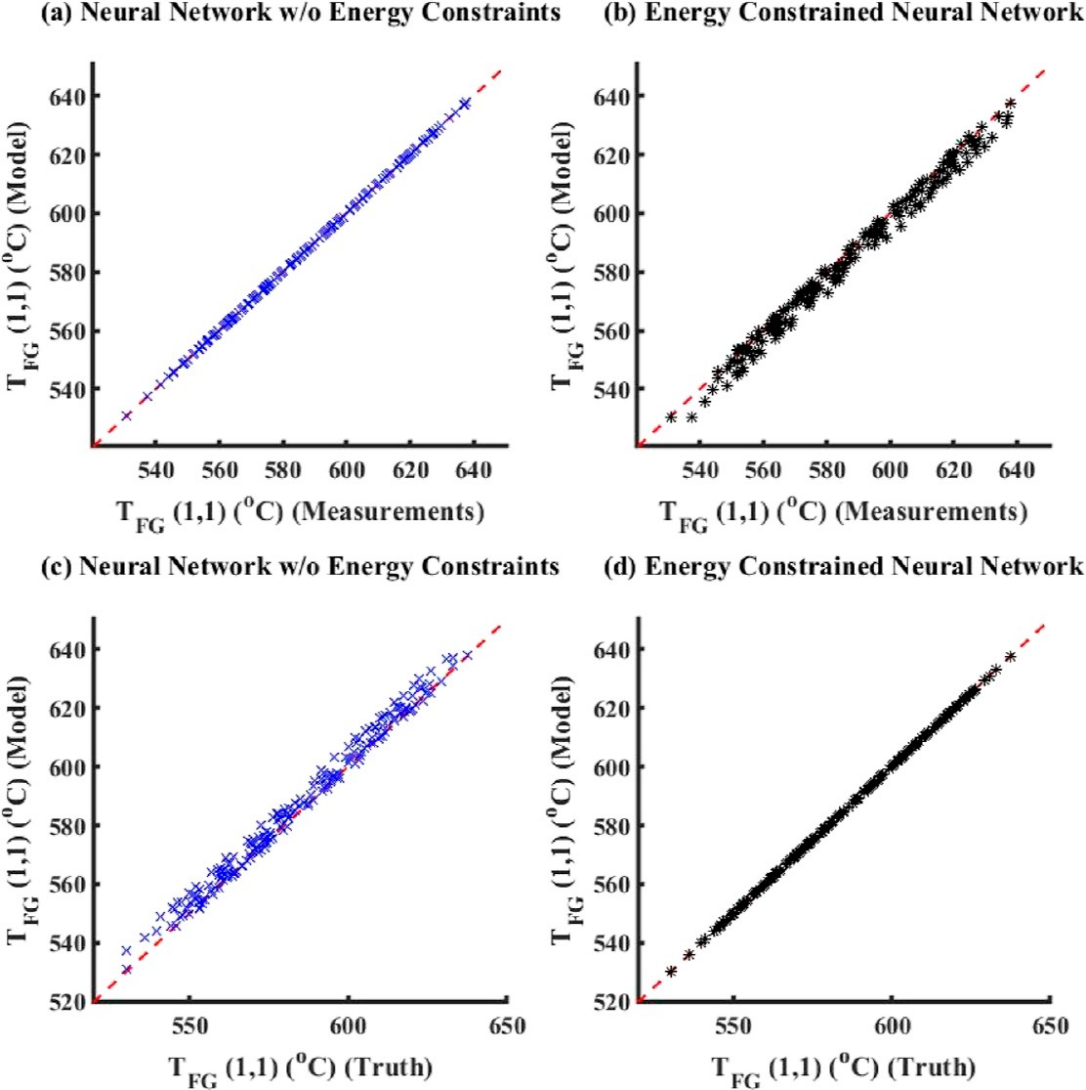
Comparison of results between ECNN and NN w/o energy constraints for the simulation data of *T*_FG,out_ at grid (1,1) (noise in the measurement data represented by eq 5).

Figure 14 shows a comparison between model results vs truth and model results vs measurements for ECNN and NN w/o energy constraints for simulation data of *T*_FG,out_ at grid (1,1) while similar results for *T*_St,out_ at the same location have been included under Figure S19 in Supporting Information S.3. As before, the NN without energy constraints yields an excellent match with respect to the measurement data represented by around 0.05 and 0.06% RMSE for flue gas and steam, respectively, but poorer results with respect to true data (average of around 0.71% RMSE). Reverse trends can be seen in the results for the ECNN. Figure S20 in Supporting Information S.3 shows that the ECNN exactly satisfies energy conservation at all grid points, whereas the unconstrained NN (w/o energy constraints) shows an APE in energy balance as high as 20%.

Figure S21 in Supporting Information S.3 shows that the ECNN exactly satisfies the energy conservation laws also at the system boundaries. Other parity plots and comparison results are not included here for brevity. Those results also show similar performance comparisons between ECNN and NN without energy constraints as compared with the figures presented here.

### 5.2 Case Study 2: Non-Isothermal van de Vusse Reactor System

Unlike Case Study 1 in which mass conservation of the system was trivially satisfied, this case study considers the imposition of both mass and energy balance constraints simultaneously during training and forward problems. The corresponding constrained neural network models will hence be referred to as MECNN, instead of ECNN. Furthermore, all training and simulation parity plots for this case study will be shown with respect to two of the output variables considered, namely the outlet concentration of A (*C*_A_) and the temperature (*T*) of the product stream. Results for the other outputs also show similar trends but have not been included here for brevity. For this case study, the APE in energy balance is defined as
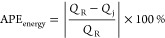
26where *Q*_R_ and *Q*_j_ refer to the total heat lost by the reactor mixture and that gained by the jacketing fluid (cooling water), respectively. The specific enthalpy terms involved in the energy balance constraint (as given by eq 17) have been appropriately expressed in terms of specific heat capacity of the reactor mixture and the heat of reaction calculated from the respective heats of formation of the reactants and products.

#### 5.2.1 No Noise

Parity plots and mass-energy balance error plots for the training data are shown in Figures S22 and S23, respectively, in Supporting Information S.3, whereas those for the simulation data have been included in Figures S24 and S25 in Supporting Information S.3.

#### 5.2.2 Constant Bias with Gaussian Noise

**Figure 15:**
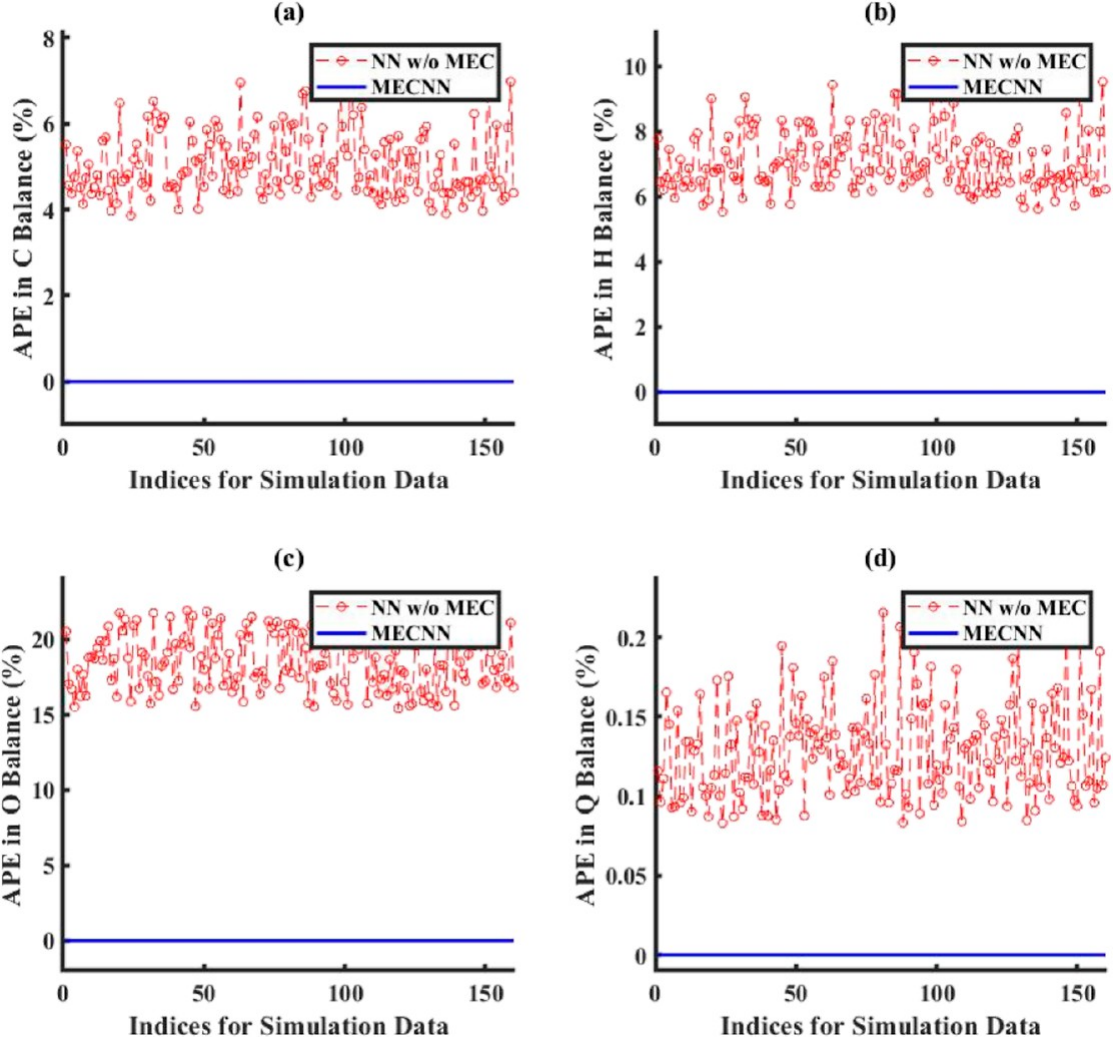
Comparison between MECNN and NN w/o mass-energy constraints in terms of violating (a) carbon (C), (b) hydrogen (H), (c) oxygen (O), and (d) energy (*Q*) balance constraints during simulation (noise in the measurement data represented by eq 4).

For this case, constant biases equal to 5% of the mean of the true concentrations and around 3.5 °C for each temperature variable are added to the respective outputs along with an additional Gaussian noise with μ = 0 and σ^2^ = 0.1. Parity plots for training data have not been included here for the sake of brevity. Figure S26 in Supporting Information S.3 shows that MECNN exactly satisfies the mass and energy conservation constraints at steady-state for training data, whereas the unconstrained NN shows significantly higher APE values both for mass and energy balance constraints. Figures S27 and S28 in Supporting Information S.3 compare the model results vs truth and measurements for MECNN and unconstrained NN for simulation data of the outlet concentration of component A in product stream (*C*_A_) and reactor outlet temperature (*T*). The unconstrained NN yields an excellent match (around 0.03% RMSE) with the measurements but results in biased estimates compared with the system truth, thus leading to around 5.3% RMSE with respect to the truth. On the contrary, MECNN accurately captures the system truth (approximately 0.01% RMSE) but shows a clear mismatch with the measurements leading to around 4.9% RMSE. Furthermore, the MECNN exactly satisfies both mass and energy balance constraints during the forward problem as well, as seen in Figure 15, whereas the NN w/o those constraints show the maximum APE in C, H, O and the energy balance of 7.4, 10.1, 21.8, and 0.22%, respectively.

#### 5.2.3 Random Bias with Gaussian Noise

**Figure 16:**
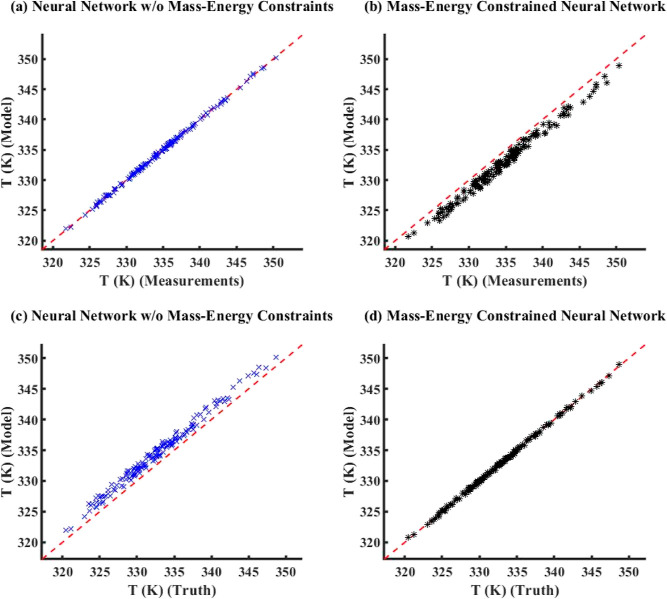
Comparison of results between MECNN and NN w/o mass-energy constraints for simulation data of outlet reactor temperature (*T*) (noise in measurement data represented by eq 5).

For this case study, a random bias within ranges of 1–8% of the true concentrations and between 1 and 4 °C for each temperature variable along with an additional Gaussian noise distribution (μ = 0, σ^2^ = 0.2) is considered. Moreover, similar to Case Study 1, the linear form of the error model yielded superior results as compared to the quadratic form as well as when a no-noise/error model is considered during the training problem. Corresponding results have not been included here for the sake of brevity. Figure 16 compares MECNN and NN w/o mass-energy constraints for simulation data of the reactor temperature (*T*), whereas the corresponding training results have been included in Figure S29 in Supporting Information S.3. RMSE calculated between the network models and training/simulation data have been summarized in Table 2. Corresponding mass and energy balance error plots for the training and forward problems have been included in Figures S30 and S31 in Supporting Information S.3.

**Table 2:** Comparison in RMSE Values between MECNN and NN w/o Mass-Energy Constraints with Respect to Measurements and True Data for Outlet Reactor Temperature (*T*) during Training and Simulation

	percentage RMSE with respect to measurements for outlet reactor temperature (*T*)		percentage RMSE with respect to true data for outlet reactor temperature (*T*)	
type of network	training data	simulation data	training data	simulation data
MECNN	0.55%	0.77%	0.03%	0.06%
NN w/o mass-energy constraints	0.04%	0.05%	0.66%	0.81%

### 5.3 Case Study 3: Electrically Heated Tubular Reactor for Cracking of Acetone

While the nonisothermal van de Vusse reactor system (Case Study 2) involves the generation of heat within the system, the third case study under consideration involves a distributed plug flow reactor where one form of energy (i.e., electric energy) gets transformed to another (i.e., heat energy). The external electric heater (EH) supplies heat energy to the system necessary for the endothermic cracking reaction of acetone to yield the desired products. Since the system involves both mass and energy transfers, both mass and energy conservation constraints need to be imposed simultaneously during formulation of training/forward problems. Accurately modeling such a type of heat transfer is relatively more challenging since the distribution is unknown for the external heat duty. Therefore, local measurements from multiple locations in the distributed system become necessary for ensuring the exact conservation of mass and energy of the reactor system at individual discretization elements as well as at the system boundaries. Similar sensitivity analyses can be performed for this case study as compared to the distributed superheater/reheater system (Case Study 1) in terms of variation in performance of optimal MECNNs with respect to spatiotemporal availability of local measurements. However, for brevity, all steady-state and dynamic results discussed in this paper for this case study correspond to the case when all local measurements are available during model development. Furthermore, only the training/simulation plots for two of the output variables, i.e., *C*_A_ and *T*_out_ have been included here. The corresponding results for the rest of the outputs show similar performances but have not been discussed in this paper for brevity. The APE in energy balance is defined as
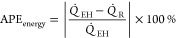
27where  and  refer to the heat duty of the external electric heater and the heat flux across the tubular reactor, respectively, for each specific discretized element (as well as the entire system). The heat flux across the reactor is calculated using the specific enthalpy balance equations involving thermo-physical properties such as specific heat capacities, standard heats of formations, etc., of the reaction species involved in the process.

#### 5.3.1 No Noise

Parity plots and mass-energy balance error plots for the training data are shown in Figures S32 and S33, respectively, in Supporting Information S.3, whereas those for the simulation data have been included in Figures S34 and S35 in Supporting Information S.3.

#### 5.3.2 Constant Bias with Gaussian Noise

For this case, constant biases equal to 7.5% of the mean of the true concentrations and around 5 °C for each temperature variable are added to the respective outputs along with an additional Gaussian noise with μ = 0 and σ^2^ = 0.25. The parity plots showing training results have not been included in this paper. Figure S36 in Supporting Information S.3 shows that MECNN exactly satisfies mass and energy balances at steady-state for training data, whereas the unconstrained NN shows significantly higher APE values for the same.

**Figure 17:**
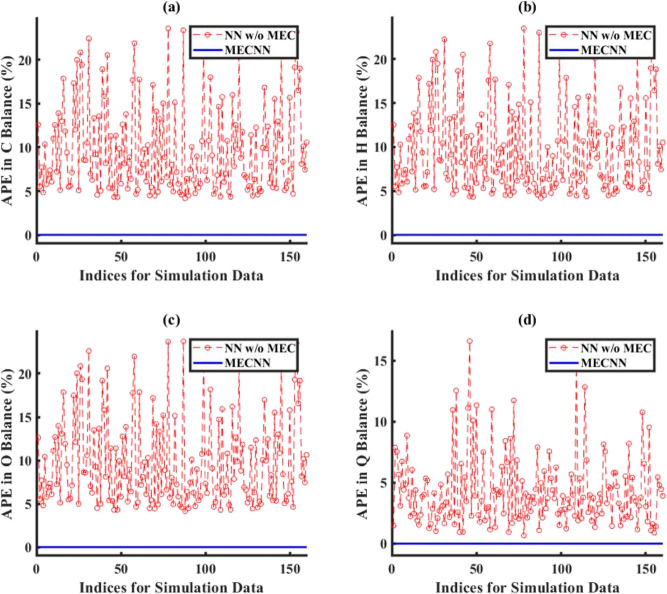
Comparison between MECNN and NN w/o mass-energy constraints in terms of violating (a) carbon (C), (b) hydrogen (H), (c) oxygen (O), and (d) energy (*Q*) balance constraints at the first discretized element during simulation (noise in the measurement data represented by eq 4).

Figures S37 and S38 in Supporting Information S.3 compare the model results vs truth and measurements for MECNN and unconstrained NN for simulation data of outlet concentration of component A (*C*_A_) and reactor outlet temperature (*T*) at the boundary of the first discretized element. The unconstrained NN yields an excellent match (represented by around 0.04% RMSE) with the measurements but results in biased estimates compared to the system truth, thus leading to around 7.6% RMSE with respect to the true data. On the contrary, MECNN accurately captures the system truth (characterized by approximately 0.01% RMSE) but shows a clear mismatch with the measurements leading to around 7.4% RMSE. Furthermore, the MECNN exactly satisfies both mass and energy balance constraints during the forward problem as well, as seen in Figure 17, whereas the NN w/o those constraints show APE in C, H, and O and energy balances as high as 24, 23, 25, and 17%, respectively.

#### 5.3.3 Random Bias with Gaussian Noise

**Figure 18:**
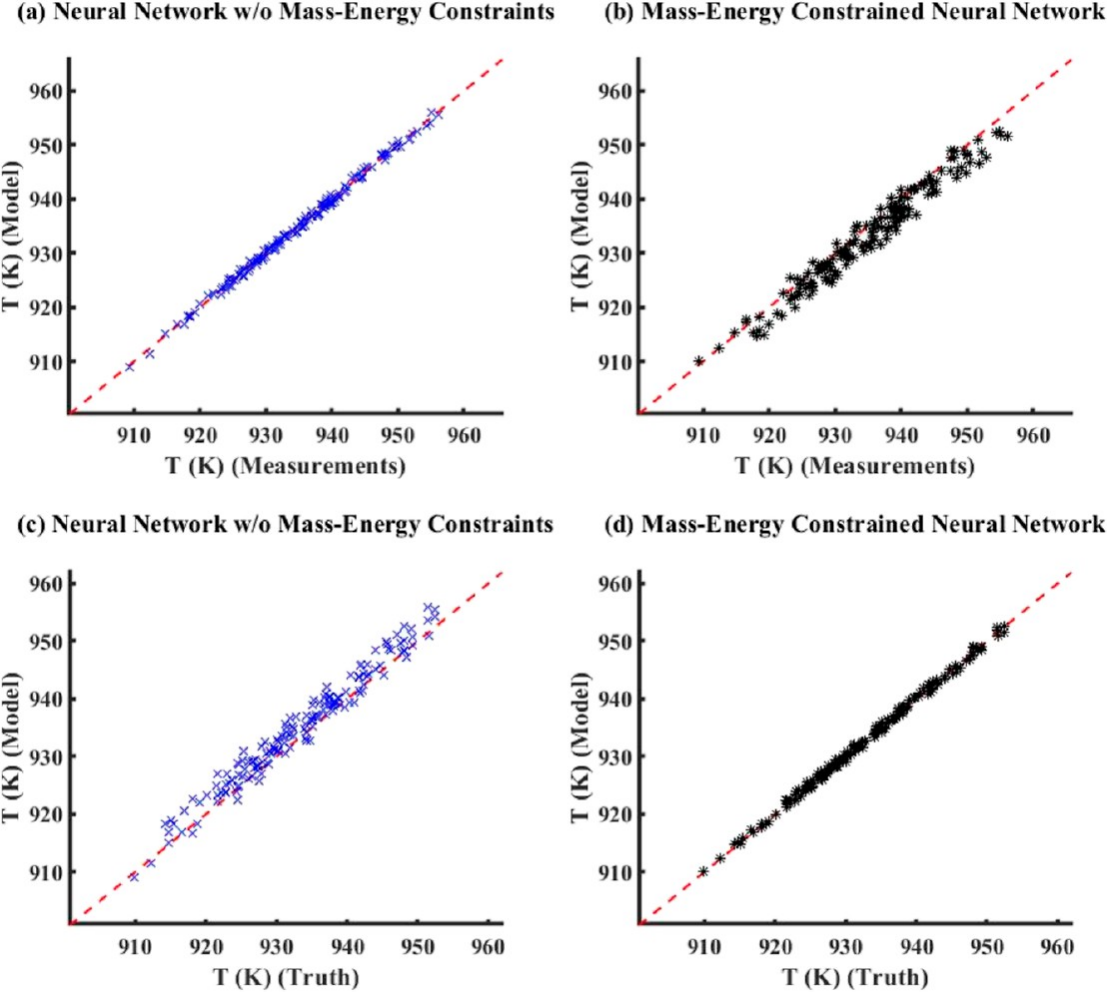
Comparison of results between MECNN and NN w/o mass-energy constraints for the simulation data of outlet reactor temperature (*T*) at the system boundary (noise in the measurement data represented by eq 5).

Here, a random bias within ranges from −10 to 25% for the true concentrations and between −2 and 6 °C for each temperature variable along with an additional Gaussian noise distribution (μ = 0, σ^2^ = 0.25) is considered. It has also been observed that the linear form of the error model yielded superior results as compared to the quadratic form as well as when a no-noise/error model is considered during the training problem. Corresponding results have not been included here for brevity. Figure 18 compares MECNN and NN w/o mass-energy constraints for simulation data of the reactor temperature (*T*) at the system boundary, whereas the corresponding results for *C*_A_ have been included in Figure S39 in Supporting Information S.3.

Similar trends in performance have been observed where the MECNN accurately captures the system truth (recording less than 0.02% RMSE) even when trained against noisy biased measurements, while reverse trends are seen for the unconstrained NN model. The corresponding mass and energy balance error plots for the training and forward problems have been included under Figures S40 and S41 in Supporting Information S.3, respectively.

## 6 Results and Discussion: Dynamic Modeling

The results discussed in this section do not include the trivial case of error characterization (eq 3), i.e., when no noise/bias is added to true data to generate training data. However, the other two types of characterizations consisting of a time-invariant (eq 4) and a time-varying (eq 5) bias in the presence of an additional Gaussian noise distribution of known variance have been analyzed for dynamic modeling using MECNNs/ECNNs. Furthermore, since all case study examples considered in this paper, except the nonisothermal van de Vusse reactor system (Case Study 2), are spatially distributed systems, the corresponding holdup information is considered to be unavailable, thus leading to applying the mass and energy conservation equations only on the steady-state zones while developing dynamic models for these systems. The unconstrained NN models for all cases of dynamic modeling implementations of MECNN/ECNN have been represented either by the respective standalone hybrid series/parallel all-nonlinear static-dynamic networks having the same architecture as those used in MECNNs/ECNNs or by one of the most widely used state-of-the-art network models for transient prediction denoted by LSTM-type RNNs.^56^

### 6.1 Case Study 1: Adiabatic Superheater/Reheater System

For developing both lumped and distributed dynamic models of the adiabatic superheater/reheater system, it is considered that system holdup information is not available.

#### 6.1.1 Lumped Parameter Model

##### 6.1.1.1 Time-Invariant Bias with Gaussian Noise

**Figure 19:**
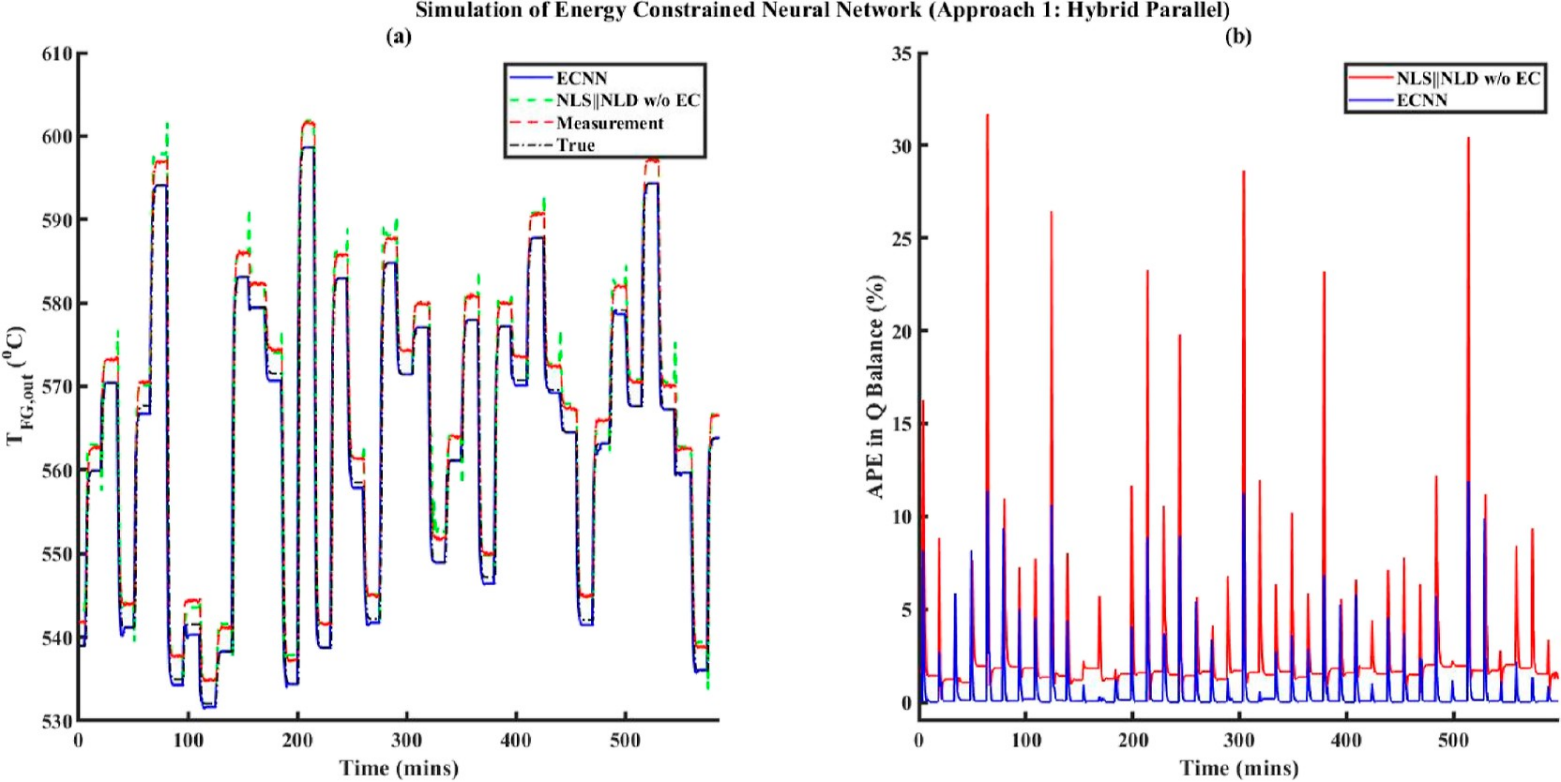
Comparison of results between hybrid parallel ECNN and NLS||NLD w/o energy constraints (a) for simulation data of *T*_FG,out_ and (b) in terms of violating energy (*Q*) balance constraints during simulation (noise in the measurement data represented by eq 4).

A time-invariant bias equaling 5 °C has been added to each model output variable to generate biased dynamic training data for the superheater system, along with an additional Gaussian noise distribution with μ = 0 and σ^2^ = 0.25. The results obtained from the training problem have not been included here for brevity. Figure 19a shows the comparison between the model results vs truth and model results vs measurements for the hybrid parallel ECNN and standalone NLS||NLD model without energy constraints for dynamic simulation data of *T*_FG,out_. Similar results for simulation data of *T*_St,out_ have been included under Figure S42 in Supporting Information S.4. From Figure 19a, it can be observed that the hybrid parallel ECNN model yields accurate results for the simulation data of *T*_FG,out_, including the transients, even though the presence of bias in training data is clear. The outlet predictions of *T*_FG,out_ obtained from the ECNN show only around 0.05% RMSE with respect to the system truth while recording a significantly larger error (represented by around 1.1% RMSE) when compared with the measurements. On the contrary, the NLS||NLD network w/o energy constraints shows an excellent match with the dynamic measurements (characterized by approximately 0.2% RMSE) but violates the true data, leading to around 1.2% RMSE with respect to the system truth. Furthermore, Figure 19b shows that the ECNN exactly satisfies the energy balance constraints at steady-state for simulation data while still showing some errors during transients due to unavailability of system holdup information. However, the unconstrained hybrid parallel (NLS||NLD) network violates energy conservation leading to as high as 32% energy balance error for the simulation results.

It is crucial to guarantee the sufficiency of steady-state zones in the overall dynamic-time series training data from the perspective of uniquely identifying network parameters. The hybrid series ECNN also yielded results similar to those of the hybrid parallel ECNN model when subjected to the same training data consisting of the time-invariant bias with Gaussian noise. However, between the two variants of hybrid series ECNNs, i.e., NLS–NLD and NLD–NLS models, the NLD–NLS type of hybrid series ECNN outperformed the other in terms of lesser computational expense for similar prediction accuracy. Figure S43 in Supporting Information S.4 shows the comparison of hybrid series (NLD–NLS) ECNN vs LSTM-type RNN (i.e., NN w/o energy constraints) for simulation data of *T*_St,out_. It has been observed that although both models are trained against the same noisy biased measurements, the dynamic ECNN accurately captures the system truth, resulting in around 0.05% RMSE with respect to the true data, whereas the LSTM-type RNN violates the energy conservation and converges farther from the true data leading to an approximate RMSE of 1.16% with the same. The main purpose of comparing the dynamic ECNN model with the LSTM-type RNN is basically to evaluate the performance of one of the most widely used RNN models such as the LSTM network in the absence of energy balance constraints. The other results obtained from hybrid series ECNN have not been included here for brevity. It is also to be noted that the hybrid parallel all-nonlinear static-dynamic networks had also been similarly implemented through this approach (i.e., considering the entire time-series data without partitioning into steady-state and dynamic zones), but it resulted in the same/similar outputs obtained from the optimal model but at the cost of a significantly higher computational expense. Therefore, since the sequential training algorithm for the hybrid parallel network readily fits into the previous approach proposed, data partitioning is preferable while implementing the hybrid parallel ECNNs for dynamic modeling.

##### 6.1.1.2 Time-Varying Bias with Gaussian Noise

**Figure 20:**
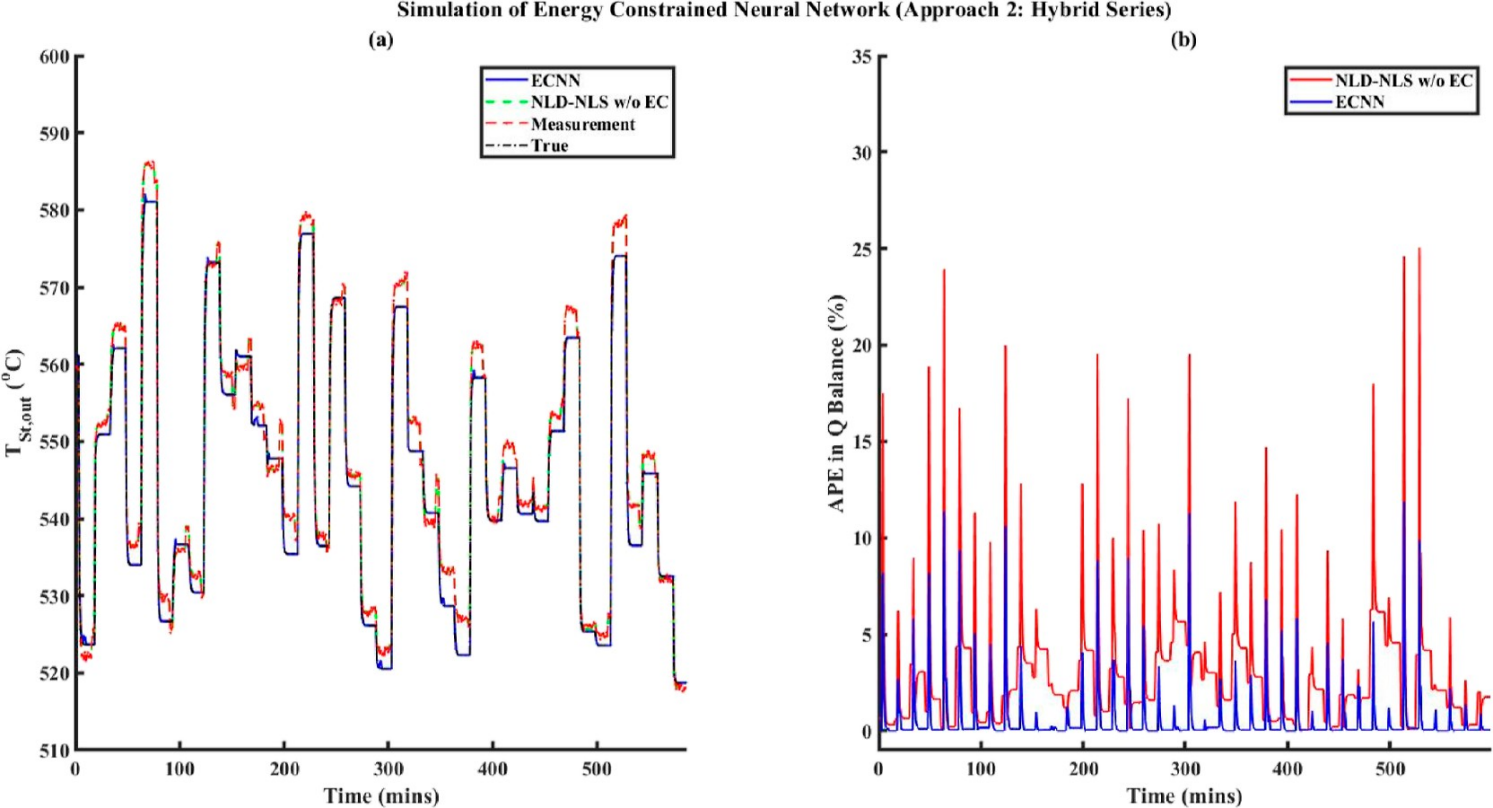
Comparison of results between hybrid series (NLD–NLS) ECNN and NLD–NLS w/o energy constraints (a) for simulation data of *T*_St,out_ and (b) in terms of violating energy (*Q*) balance constraints during simulation (noise in the measurement data represented by eq 5).

In addition to the time-invariant bias, a time-varying bias within the range of −2.5 to 6.5 °C along with an additional Gaussian noise has also been considered to generate training data for the lumped parameter model of the superheater/reheater system. As before, the results obtained from the training problem are not included for brevity. Figure 20a compares the results obtained from the hybrid series (NLD–NLS)-type ECNN to those from an unconstrained NN model having the same architecture (i.e., all-nonlinear hybrid NLD–NLS network model) for the simulation data of *T*_St,out_. The hybrid series ECNN model yields accurate results for the simulation data including the transients, leading to an overall RMSE of around 0.03% with respect to the true data. On the contrary, the NLD–NLS model without energy constraints violates the energy conservation laws (about 0.75% RMSE with respect to the true data), albeit showing an excellent match with the measurements (approximately 0.05% RMSE). The maximum APE in energy balance for the unconstrained NLD–NLS model can also be seen to be as high as 26% while the ECNN exactly satisfies the energy conservation constraints under steady-state operation, though there exists some mismatch during the transients due to lack of holdup information, thus leading to a maximum instantaneous error of around 12%, as can be seen in Figure 20b. A linear noise model has been considered for development of the dynamic model for the lumped system under time-varying bias to address the lack of identifiability since the linear model form for the error (noise) model showed superior performance in terms of prediction accuracy and optimal AIC_c_ values as compared to the cases when no error model or a quadratic error model has been considered, although all three cases eventually led to the exact satisfaction of energy balance constraints at steady-state during the training problem. For example, when no error model was considered during optimal model synthesis, the flue gas and steam outlet temperatures predicted by the optimal ECNNs converged far away from the system truth, thus recording around 0.92 and 1.14% RMSE, respectively, with the corresponding true data.

The hybrid parallel ECNN, along with the incorporated linear noise model, also yielded similar results for this case of error characterization for the lumped superheater/reheater system. Figure S44 in Supporting Information S.4 compares the hybrid parallel ECNN and the unconstrained NLS || NLD model for simulation data of *T*_FG,out_. It is to be noted that for all case studies, the development of hybrid series ECNN/MECNN models corresponds to a significantly higher computational expense as compared to the hybrid parallel ECNN/MECNN models while solving the training problem since the former class of networks incorporates the entire time-series data in the equality-constrained parameter estimation problem. The other results obtained from the hybrid series and parallel ECNNs where the measured data are corrupted with a time-varying bias have not been included here for the sake of conciseness.

#### 6.1.2 Distributed Model

##### 6.1.2.1 Time-Invariant Bias with Gaussian Noise

**Figure 21:**
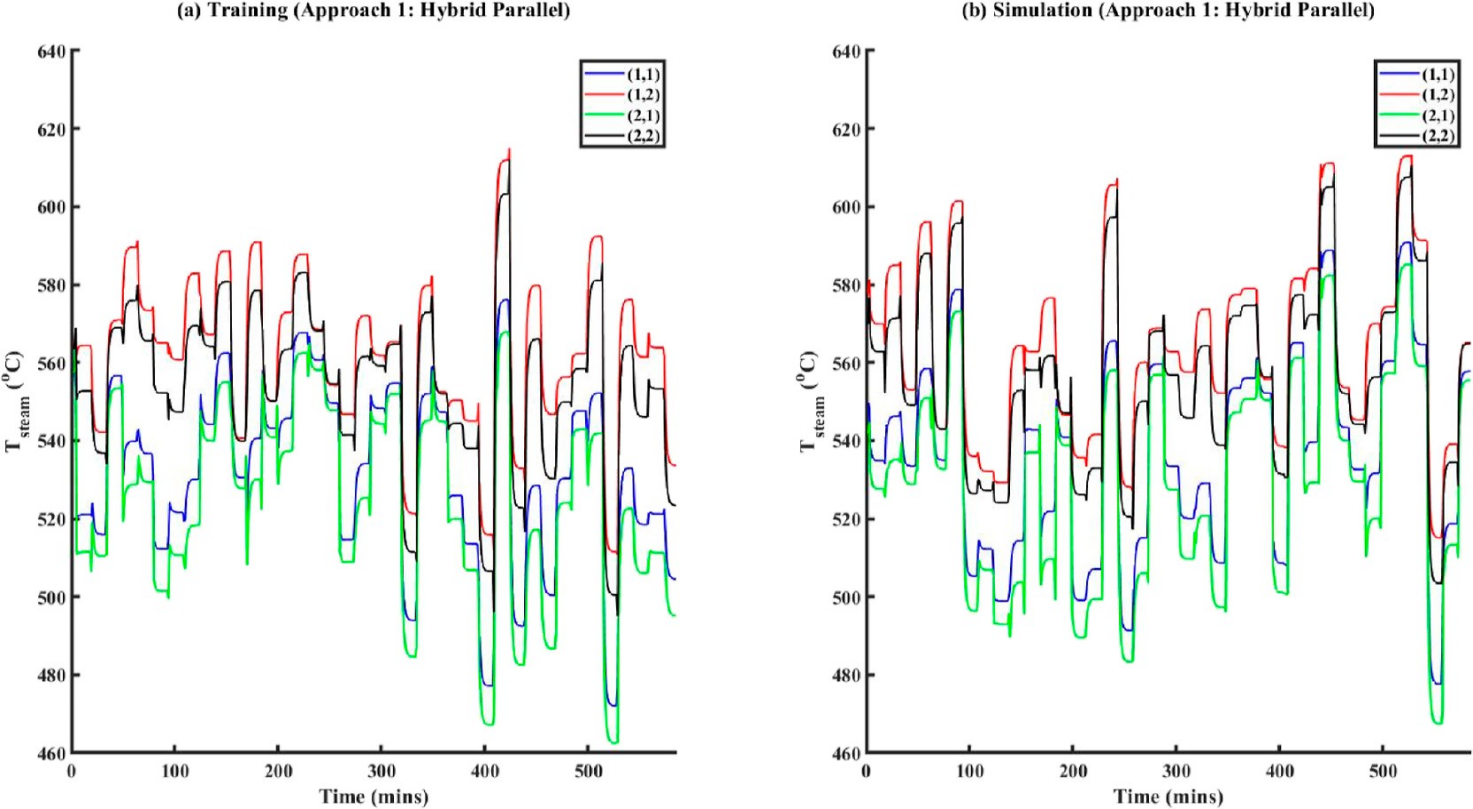
Results from hybrid parallel ECNN for temporal *T*_St,out_ distribution in spatial coordinates during (a) training and (b) simulation (noise in measurement data represented by eq 4).

Similar to the lumped parameter model example, a time-invariant bias equaling to around 5 °C has been added to each output temperature variable to generate biased dynamic training data for the distributed superheater system, along with an additional Gaussian noise distribution with μ = 0 and σ^2^ = 0.25. The principal motivation for developing a distributed model as opposed to a lumped parameter model is to gain insights into predicting the temperature distributions at intermediate discretization grids within the superheater/reheater system. For all results shown in this section, the neural networks without energy constraints are represented by the networks with the same series/parallel architectures as the hybrid ECNN model, which have been trained against the measurement data in the absence of any energy balance constraints. Figures S45 and S46 in Supporting Information S.4 compare the hybrid parallel ECNN with the NLS||NLD model without energy constraints for the simulation data of *T*_FG,out_ and *T*_St,out_ at one of the intermediate grids represented as (1,2). As before, the hybrid parallel ECNN yields accurate results with respect to the system truth including the transients even though the presence of the bias in the data is clear, thus leading to around 0.08 and 0.73% RMSE, respectively, with true data and measured data. Reverse trend is observed for the NLS||NLD model without energy constraints, which record 0.74 and 0.12% RMSE when compared to the true data and measurements, respectively. Furthermore, the distributed model also ensures that the energy conservation laws are exactly satisfied not only at the system boundaries but also at all intermediate discretization grids under consideration. Figure S45b in Supporting Information S.4 shows that the hybrid parallel ECNN exactly satisfies the energy conservation constraint at steady-state (the instantaneous spikes on the error plot for ECNN are due to unavailability of holdup information during transients), while the unconstrained network shows maximum APE in *Q* balance at grid (1,2) as high as 12%. The comparison of the constrained and unconstrained models in terms of error in energy balance evaluated at the system boundary has been included under Figure S47 in Supporting Information S.4. Since this case study considers a spatially distributed system, the holdup information cannot be derived from system boundary conditions, and hence, no guarantee can be provided on exactly conserving the overall mass of the system during transients. The distributed ECNN model for the superheater/reheater system also provides the temporal temperature distribution in the spatial coordinates within the system during both training and forward (simulation) problems, as shown in Figure 21.

As expected, with respect to the schematic of the distributed model given in Figure 5b, the outlet steam temperature at (1,1) is consistently higher than that at (2,1) even though *T*_St,in_ for both these grids is the same since the (1,1) grid/block has the highest heat transfer gradient when the flue gas first comes in contact with steam and the gradient gradually decreases at subsequent discretized element(s) in the horizontal direction. Accordingly, the same trend is also observed for *T*_St,out_ at (1,2) and (2,2). The hybrid series ECNNs have also been implemented for this case study, and the results thus obtained show similar accuracy as compared to the hybrid parallel ECNN. However, unlike the lumped parameter model, between the two variants of hybrid series ECNNs, the NLS–NLD type of model outperforms the NLD–NLS network in terms of achieving the trade-off between prediction accuracy and computational expense based on the relative differences in model architecture and respective sequential training algorithms presented in one of our previous publications.^2^Figures S48 and S49 in Supporting Information S.4 compare model results vs truth and model results vs measurements for the hybrid series (NLS–NLD) ECNN and unconstrained NLS–NLD model for simulation data of *T*_FG,out_ and *T*_St,out_, respectively, at the intermediate discretization element represented by (1,1). Other training/simulation results at different locations in the spatial coordinates for both hybrid series and parallel ECNNs for this type of error characterization have not been discussed in this paper for brevity.

##### 6.1.2.2 Time-Varying Bias with Gaussian Noise

**Figure 22:**
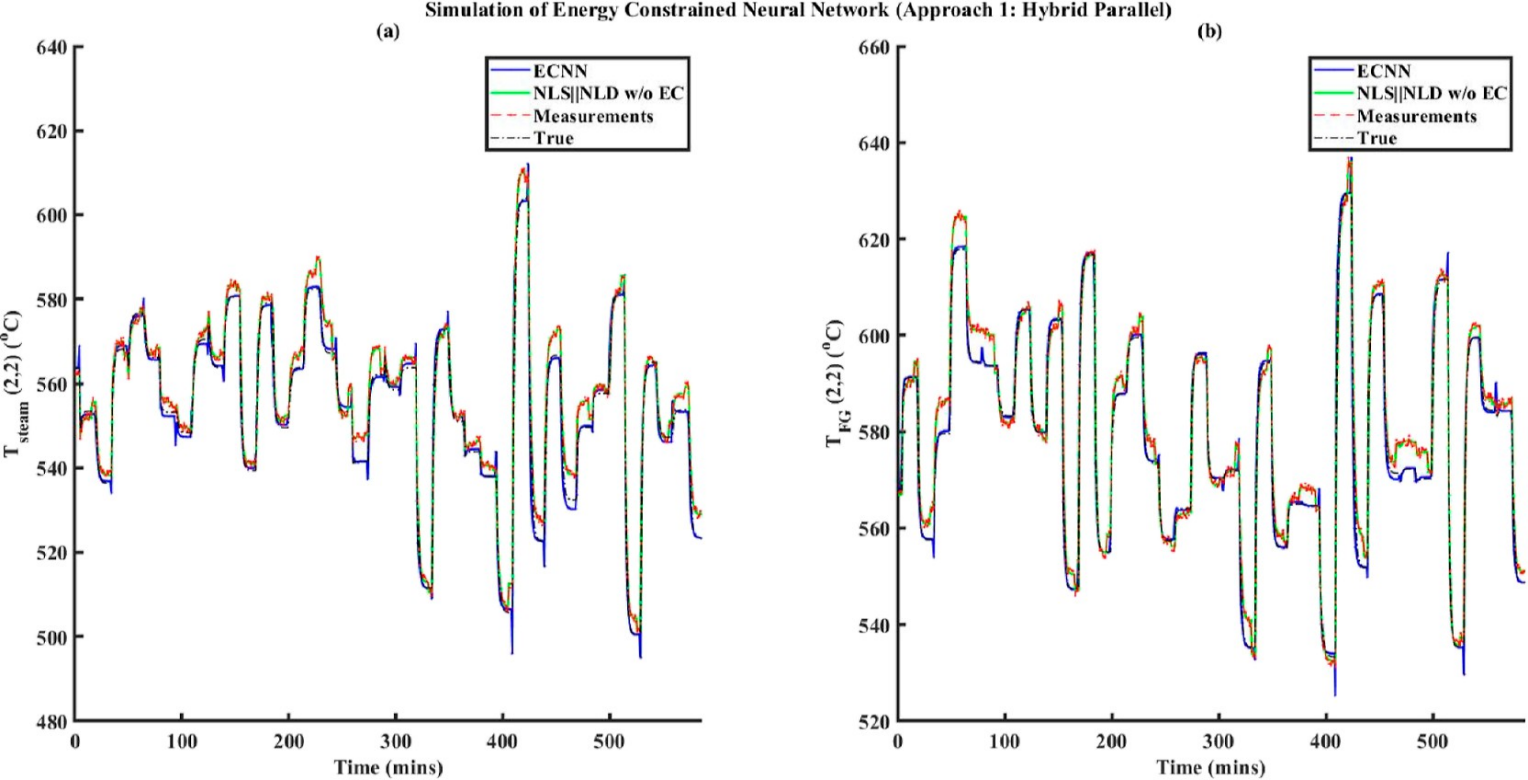
Comparison of results between hybrid parallel ECNN and NLS||NLD w/o energy constraints for simulation data of (a) *T*_St,out_ and (b) *T*_FG,out_ at grid (2,2) (noise in the measurement data represented by eq 5).

When the measurements are corrupted with a time-varying bias (as discussed in eq 5), it may lead to a lack of identifiability unless an additional noise model is considered to ensure that the optimal ECNN models converge close to the system truth during both training and forward problems. In this case, a time-varying bias within the range of −3 °C to +9 °C is considered along with an additional Gaussian noise with μ = 0 and σ^2^ = 0.5. Figures 22 and S50 in Supporting Information S.4 compare the hybrid parallel and series ECNNs with their respective unconstrained model forms for simulation data of *T*_St,out_ and *T*_FG,out_ at intermediate discretization elements referred to as (2,2) and (2,1), respectively.

As before, the hybrid parallel and series ECNN models yield accurate results for the system truth, leading to percentage RMSE values ranging between 0.08 and 0.14% with respect to the true data. On the contrary, the unconstrained models show excellent match with the measurements (resulting in around 0.09–0.12% RMSE) but violate the energy conservation of the system, thus recording higher percentage RMSE values (approximately 0.88–1.04% RMSE) with respect to the system truth. The dynamic ECNNs have also been shown to exactly satisfy energy conservation at steady-states (with slight instantaneous errors during transients), while the unconstrained models record significantly higher APE values in energy balance during both forward and training problems. Corresponding plots are not included for brevity. Similar to the random bias type of error characterization for steady-state modeling, a linear noise model has been considered for dynamic modeling under time-varying biases to address the lack of identifiability. As shown in Table 3 (with respect to the hybrid parallel ECNN model), the linear model showed superior performance as compared to the case when no error model or a quadratic model form for the error model has been considered, albeit the exact satisfaction of energy balance constraints at steady-state was achieved for both the cases. From Table 3, it is observed that if the error (noise) model had not been considered, ECNN predictions yield RMSE values of 1.22 and 1.08% for simulation data of *T*_St,out_ and *T*_FG,out_, respectively, while still exactly satisfying energy conservation at steady-state, i.e., the solution of training problem did not converge close to the truth.

**Table 3:** Comparison in % RMSE Values for *T*_St,out_ and *T*_FG,out_, and APE in Energy Balance for Simulation Data between Linear vs Quadratic vs No-Noise Model Considered in Hybrid Parallel ECNN to Address Time-Varying Bias in Measurements

	percentage RMSE between ECNN and true data during simulation for		APE in energy balance for simulation data		
type of noise model in ECNN	*T*_St,out_	*T*_FG,st_	mean APE	max instantaneous APE	AIC_c_ during training
no noise/error model	1.22%	1.08%	1.82%	21.85%	2988
linear model	0.10%	0.09%	0.41%	8.28%	2068
quadratic model	0.53%	0.65%	0.46%	12.44%	2279

### 6.2 Case Study 2: Non-Isothermal van de Vusse Reactor System

For the results presented in Sections 6.2.1 and 6.2.2, it is assumed that holdup information is not available, while results presented in Section 6.2.3 assume the availability of holdup measurements. For this case study as well, the neural networks without mass and energy constraints are represented by the networks with the same series/parallel architectures as the hybrid MECNN models, which have been trained against the measurement data in the absence of any mass-energy balance constraints.

#### 6.2.1 Time-Invariant Bias with Gaussian Noise (Holdup Measurement Not Available)

**Figure 23:**
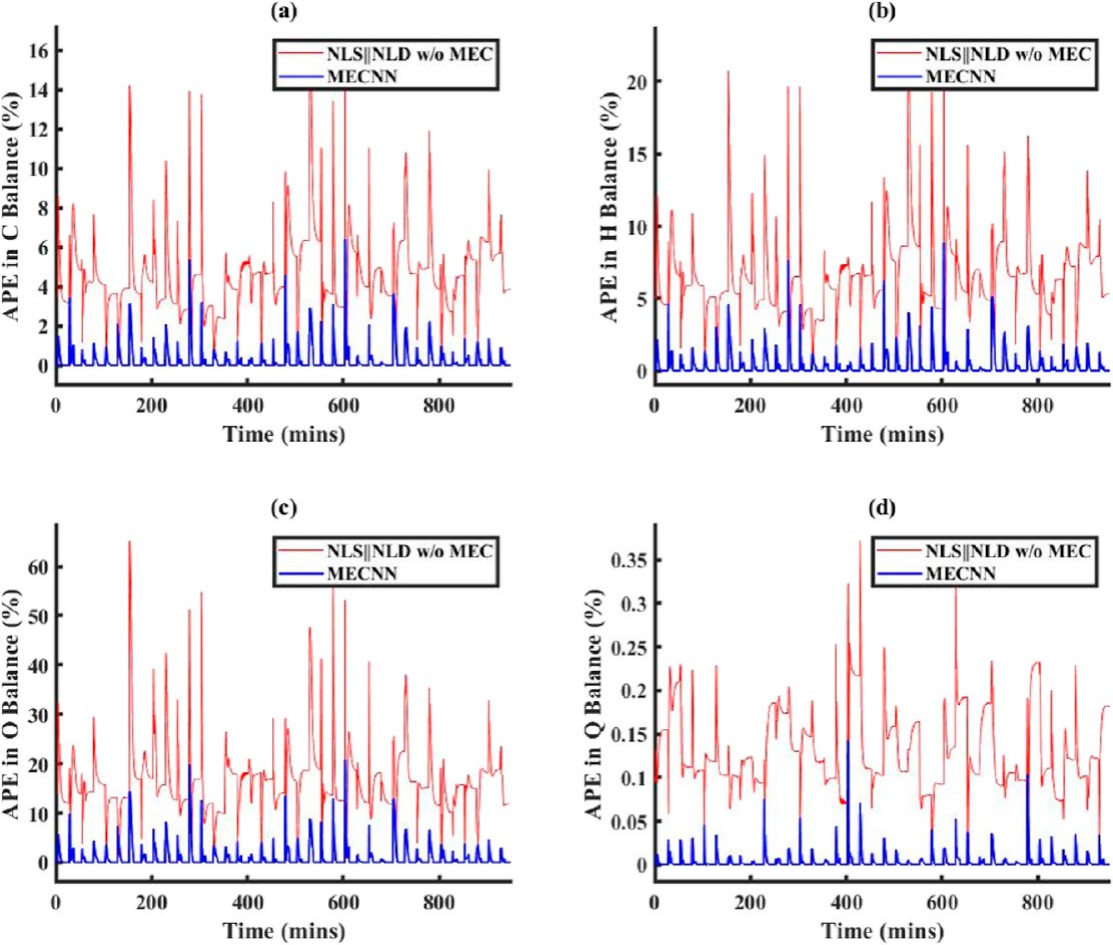
Comparison between hybrid parallel MECNN and NLS||NLD model w/o mass-energy constraints in terms of violating (a) carbon (C), (b) hydrogen (H), (c) oxygen (O), and (d) energy (*Q*) balance constraints during simulation (noise in the measurement data represented by eq 4).

For this case, time-invariant biases equal to 5% of the mean of the true output concentrations and around 4 °C for each output temperature variable are added to the respective outputs along with an additional Gaussian noise with μ = 0 and σ^2^ = 0.1. Performance plots for training data have not been included here for the sake of brevity. Figure S51 in Supporting Information S.4 shows the comparison between the model results vs truth and measurements for the hybrid parallel MECNN and the unconstrained NLS||NLD network model, respectively, for the dynamic simulation data of *C*_A_. It can be observed that the MECNN model yields accurate predictions of the system truth represented by an approximate RMSE of 0.03%, whereas it shows significantly larger errors (around 4.8%) with the measured data. As expected, reverse trends can be seen for the standalone hybrid parallel network model without mass and energy constraints. Figure 23 shows that the MECNN exactly satisfies the mass and energy balance constraints at steady state, while the unconstrained network model records maximum APE in C, H, O, and *Q* balance as 15, 20, 60 and 0.38%, respectively. The hybrid series (NLS–NLD for this system) MECNN model has also been implemented for this case study example, and the training/simulation results obtained from the optimal hybrid series MECNN model show similar performance under the presence of a time-invariant bias in the training data. Figure S52 in Supporting Information S.4 compares model results vs truth and model results vs measurements for the hybrid series (NLS–NLD) MECNN and unconstrained NLS–NLD model for simulation data of reactor temperature (*T*). Other training/simulation results for both hybrid series and parallel MECNNs for this type of error characterization have not been discussed in this paper for brevity.

#### 6.2.2 Time-Varying Bias with Gaussian Noise (Holdup Measurement Not Available)

**Figure 24:**
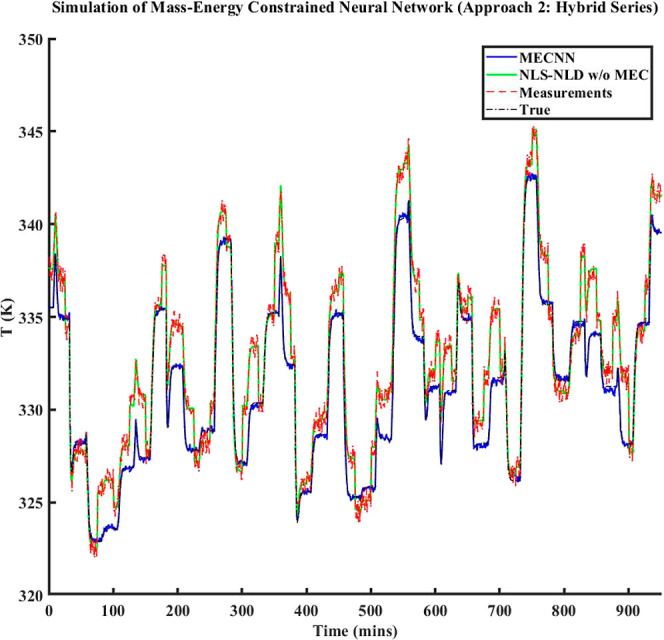
Comparison of results between hybrid series MECNN and NLS–NLD w/o mass-energy constraints for simulation data of reactor outlet temperature (*T*) (noise in the measurement data represented by eq 5).

Similar to previous cases, different parametric forms of a noise (error) model, such as linear, quadratic, etc. have been considered during optimal model synthesis when the measured data are corrupted with a time-varying bias. In this case, a time-varying bias within the range of −4 to +6% of the true outlet concentrations has been added to the concentration variables and that within −1 to 6 °C has been incorporated into the true temperature variables to generate training data, in the presence of an additional Gaussian noise distribution with μ = 0 and σ^2^ = 0.1. The linear error model again yielded superior results in terms of percentage RMSE with respect to the true data as well as the minimum AIC_c_ value evaluated during model training, when compared to the cases involving a quadratic error model or no separate error model at all. Figure 24 shows the comparison between the results obtained from hybrid series (NLS–NLD) MECNN versus unconstrained network for simulation data of reactor outlet temperature (*T*).

While the unconstrained hybrid NLS–NLD model shows an excellent match with the measurements (represented by around 0.09% RMSE), the results thus obtained violate the mass and energy conservation of the system, leading to approximately 2.5% RMSE with the true data. On the other hand, the hybrid series MECNN shows a significant mismatch with the measured data (characterized by around 2.8% RMSE) but accurately captures the system truth, recording an overall percentage RMSE of just 0.04%. Similar results for simulation data of *C*_A_ obtained from the hybrid parallel MECNN model have been included under Figure S53 in Supporting Information S.4. The comparison between the constrained and unconstrained hybrid series MECNN models in terms of violating the mass (atom) and energy conservation laws of the system can be seen in Figure S54 in Supporting Information S.4. The MECNN exactly satisfies the C, H, O, and energy balances at steady-state while showing some mismatch during transients. However, the unconstrained NLS–NLD model shows APE in the mass and energy conservation equations as high as 22, 30, 60, and 0.5%.

#### 6.2.3 Time-Invariant Bias with Gaussian Noise (Holdup Measurement Available)

In all results presented so far for dynamic modeling implementations of hybrid series and parallel MECNN/ECNN models, it can be seen that even though the hybrid series and parallel MECNN/ECNN models show excellent matches with the true dynamic profiles of model outputs, imposing the mass and energy conservation laws only during the steady-state zones is not sufficient to exactly satisfy such constraints during the transients in the system because it only uses a subset of input/output boundary conditions during both training and forward problem formulations. Since this case study example is essentially a CSTR, the concentrations of the reaction species and the instantaneous temperature of the reaction mixture inside the reactor at any given point of time can be assumed to be exactly equal to the outlet concentrations and temperature of the same at that time instant. For the specific implementation with dynamic hybrid series (NLS–NLD) MECNN models for the case when holdup information is available, the inlet feed flow rate and inlet concentrations/temperatures of all reaction species (A, B, C, and D) in the feed stream as well as those across the cooling jacket have been fixed at constant values. However, the outlet volumetric flow rate of the product stream changes with time due to the dynamic variation in the reactor volume (V) of the CSTR system. Furthermore, the availability of holdup information/measurements eliminates the need for partitioning the entire time-series data into steady-state and dynamic zones since the mass balance constraints can now be applied on all data-points. Therefore, only the hybrid series type of MECNN models have been used for modeling this case with the reactor volume (*V*) as model input and the outlet concentrations of all reaction species (i.e., *C*_A_, *C*_B_, *C*_C_, and *C*_D_) as well as the outlet temperatures of the reaction product (*T*) and jacketing fluid (*T*_*j*_) as model outputs. Furthermore, this type of analysis can be performed for any type of error characterization considered during model development. For the sake of brevity, we have not included the corresponding results obtained for the case where the measurements are corrupted with a time-varying bias.

**Figure 25:**
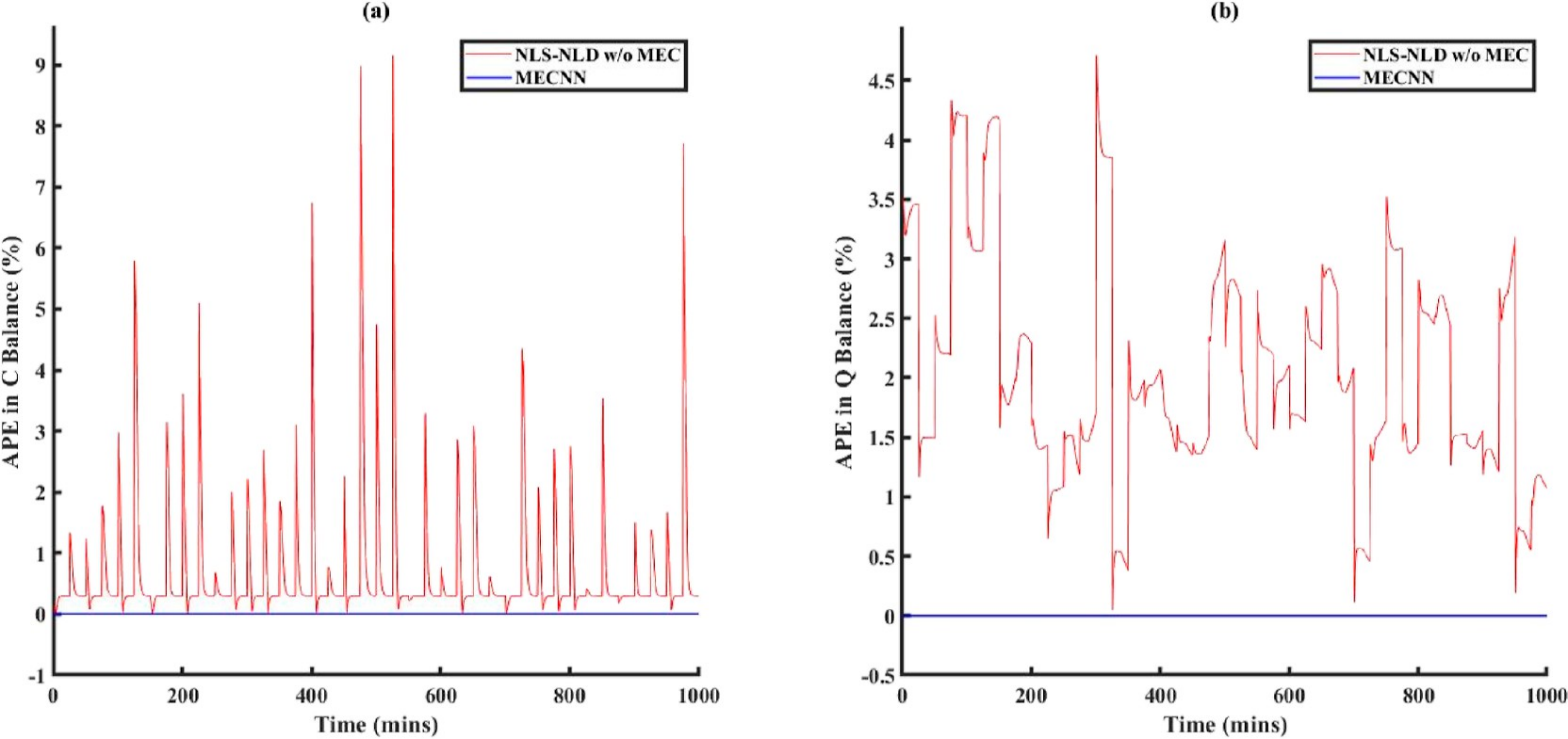
Comparison of results between hybrid series (NLS–NLD) MECNN and unconstrained NLS–NLD in terms of violating (a) carbon (C) and (b) energy (*Q*) balance constraints during simulation when holdup information is available (noise in measurement data represented by eq 4).

Figure S55 in Supporting Information S.4 shows the simulation results for *T* when a time-invariant bias equal to around 5 °C had been added to the true temperature values to generate noisy/biased training data, in the presence of an additional Gaussian noise with zero mean and known variance (σ^2^ = 0.25). As before, MECNN accurately captures the system truth (with around 0.04% RMSE) even when trained against data that violate the same. However, unlike previous cases, the MECNN exactly satisfies the mass and energy conservation even during the transients due to the imposition of mass and energy balance constraints at every time step of the dynamic training/simulation data. The unconstrained NLS–NLD model, for this case, shows significantly higher APEs in C, and energy balances with maximum errors of 9 and 4.5%, respectively, as shown in Figure 25.

### 6.3 Case Study 3: Electrically Heated Tubular Reactor for Cracking of Acetone

For the third case study, it is assumed that the holdup measurements are not available since the system is distributed. Therefore, the mass and energy conservation constraints can be applied only at steady-state zones in the overall dynamic time-series data. Furthermore, similar to previous examples, the unconstrained neural networks are represented by the NN models with the same series/parallel architectures as the optimal hybrid MECNN models.

#### 6.3.1 Time-Invariant Bias with Gaussian Noise

**Figure 26:**
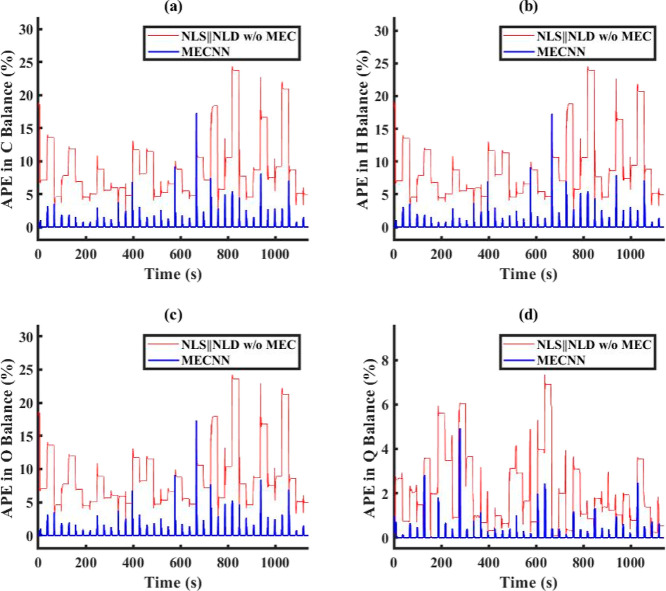
Comparison between hybrid parallel MECNN and NLS||NLD model w/o mass-energy constraints in terms of violating (a) C, (b) H, (c) O, and (d) energy (*Q*) balance constraints at system boundary during simulation (noise in the measurement data represented by eq 4).

The same measure of time-invariant (constant) bias and the Gaussian noise distribution as compared to the corresponding steady-state MECNN implementation in this case study example is considered here. Performance plots comparing the hybrid series/parallel MECNN and the respective unconstrained network models for training data have not been included here for brevity. Figure 26 shows that the MECNN exactly satisfies the mass and energy balance constraints at steady state, while the unconstrained network model record maximum APE in C, H, and O and energy balance as high as 25, 24, 23, and 7.5%, respectively, during simulation.

However, since the holdup measurements are not available during forward/training problems, the optimal MECNN still shows some instantaneous errors during the transients. The hybrid series (NLS–NLD for this system) MECNN model has also been implemented for this case study example, and the training/simulation results obtained from the optimal hybrid series MECNN model show similar performance under the presence of a time-invariant bias in the measurements (training data).

Furthermore, since the system under consideration is distributed, the corresponding optimal MECNN model also provides the distribution of the output variables at intermediate discretization grids along the axial direction of the tubular reactor. Figure S56 in Supporting Information S.4 shows the temporal distribution of outlet concentration of A (*C*_A_) and outlet reactor temperature (*T*) in the spatial (axial) coordinates obtained from the hybrid series MECNN during model simulation. As expected, the concentration of the reactant, i.e., acetone (*C*_A_) decreases gradually along the axial coordinates of the plug flow reactor, leading to the reduction in the temperature of the reaction mixture. Similar results have been observed for the other output variables using both hybrid parallel and series MECNN models but have not been discussed in this paper for brevity.

#### 6.3.2 Time-Varying Bias with Gaussian Noise

Different parametric forms of a noise (error) model, such as linear, quadratic, etc. have been considered during training MECNNs for this case. A time-varying bias within the range of −10 to +25% of the true outlet concentrations has been added to the concentration variables and that within −2 to +6 °C has been incorporated into the true temperature variables to generate training data, in the presence of an additional Gaussian noise distribution with μ = 0 and σ^2^ = 0.25. The linear error model again yielded superior results in terms of percentage RMSE with respect to the true data as well as the minimum AIC_c_ value evaluated during model training when compared to the cases involving a quadratic error model as well as that involving no separate error model at all. Figure S57 in Supporting Information S.4 shows the temporal distribution of outlet concentration of A (*C*_A_) and outlet reactor temperature (*T*) in the spatial (axial) coordinates obtained from the hybrid parallel MECNN during model simulation subject to data corrupted by a time-varying bias. Other performance plots obtained during forward/training problems using hybrid parallel MECNNs have not been included here for brevity.

Figure S58 in Supporting Information S.4 shows the comparison between the results obtained from hybrid series (NLS–NLD) MECNN vs unconstrained network for simulation data of *C*_A_ at the system boundary. From Figure S4.17, it can be observed that while the unconstrained hybrid NLS–NLD model shows an excellent match with the measurements (around 0.9% RMSE), the results thus obtained violate the mass and energy conservation of the system leading to approximately 7.0% RMSE with the true data. On the other hand, the hybrid series MECNN shows a significant mismatch with the measured data (characterized by around 6.8% RMSE) but accurately captures the system truth recording an overall percentage RMSE of just 0.05%. Furthermore, the optimal MECNN also exactly satisfies the mass and energy conservation constraints at steady-state, as shown in Figure S59 in Supporting Information S.4, while the unconstrained NN model records significantly higher instantaneous constraint violations.

## 7 Conclusions

In this study, we developed algorithms for training and simulating steady-state and dynamic neural network models capable of exactly satisfying mass and energy conservation laws, even when the training data used for optimizing the networks violate these laws. Furthermore, we have also shown that if dynamic system holdup information is available, then these algorithms can also satisfy mass and energy conservation constraints during process dynamics. The MECNNs developed for modeling distributed systems have illustrated the importance of local measurements during the construction of optimal data-driven models. For all case studies presented, the steady-state and dynamic MECNNs consistently exhibited an RMSE of less than 0.5% when compared to the true data for both training and forward problems. On the contrary, the corresponding unconstrained network models resulted in significantly higher errors, leading to maximum values around 2.6, 0.9, 5.3, and 7.6% with respect to the truth for the lumped superheater, distributed superheater, nonisothermal van de Vusse reactor, and electrically heated tubular reactor, respectively. Additionally, the unconstrained networks also recorded notably higher percentage errors in mass (atom) and energy (enthalpy) balances.

The developed algorithms also address the potential issues arising from a lack of parametric identifiability in the presence of a time-varying bias in the data and compare different forms of noise models for optimal efficiency during training problem. It has been observed that the linear noise model considered in the training problem, when the training data are corrupted with random/time-varying bias, yields the most superior results consistently in terms of AIC_c_ values obtained during model training. The MECNN structures developed as part of this work can be applied not only for steady-state modeling but also for modeling dynamic process systems using noisy transient measurements. However, it is worth noting that for spatially distributed systems, exact satisfaction of mass and energy conservation laws may not be guaranteed during transients due to the unavailability of sufficient information about local system holdup.

Finally, it is important to clarify that while our proposed algorithms do not guarantee perfect predictions of the true data for any given training data set, they do ensure that mass and energy balance constraints are exactly satisfied during both training and forward (simulation) problems.

## Data Availability

The Python and MATLAB codes for steady-state and dynamic ECNNs (MECNNs) developed in this work can be found on GitHub at https://github.com/mukherjeeangan26/Steady_Dynamic_ECNN. Similar codes for steady-state and dynamic MCNNs can also be publicly accessed on GitHub at https://github.com/mukherjeeangan26/Steady_Dynamic_MCNN. Additionally, the MATLAB codes developed for training the unconstrained hybrid series and parallel all-nonlinear static-dynamic neural network models are also available on GitHub at https://github.com/mukherjeeangan26/Hybrid_StaticDynamic_NN_Models.

## Supporting Information Available

Additional structures/algorithms for MECNNs; additional details about the case study examples under consideration; additional training/simulation results for steady-state MECNNs; and additional training/simulation results for dynamic MECNNs (PDF)
