# Supplementary material for: Development of Steady-State and Dynamic Mass and Energy Constrained Neural Networks for Distributed Chemical Systems Using Noisy Transient Data
Source: Ind Eng Chem Res. 2024 Aug 2;63(32):14211–39. doi: 10.1021/acs.iecr.4c01429 (PMC11328135; doi:10.1021/acs.iecr.4c01429)
Supplement: Supplementary file 1 — ie4c01429_si_001.pdf [file ie4c01429_si_001.pdf]

Development of Steady-State and Dynamic Mass and Energy Constrained Neural Networks for Distributed Chemical Systems Using Noisy Transient Data

Angan Mukherjee, Debangsu Bhattacharyya*

Department of Chemical and Biomedical Engineering, West Virginia University, Morgantown, WV 26506, USA

*Corresponding author. Tel:+1-3042939335, Fax: +1-3042934139

E-mail address: Debangsu.Bhattacharyya@mail.wvu.edu

Supporting Information (SI)

The following items are provided in the supporting information document for brevity.

- **S.1:** Additional Structures / Algorithms for MECNNs

- **S.2:** Additional Details about the Case Study Examples under Consideration

- **S.3:** Additional Training / Simulation Results for Steady-State MECNNs

- **S.4:** Additional Training / Simulation Results for Dynamic MECNNs

S.1: Additional Structures / Algorithms for MECNNs

This section consists of additional network architectures and training / simulation algorithms considered during development of steady-state and dynamic MECNNs in this work.

S.1.1. Hybrid Series and Parallel All-Nonlinear Static-Dynamic Network Models

The nonlinear static (NLS) model, represented by MLFFNN and nonlinear dynamic (NLD) model, denoted by NARX-type RNN can be connected with each other, both in series or parallel configurations, to develop fully nonlinear hybrid series and parallel static-dynamic networks. For the series configuration, two different structures are possible – the MLFFNN followed by the NARX-type RNN, which is referred to as the NLS-NLD type model and the reverse arrangement, i.e., the NARX-type RNN followed by the MLFFNN, referred to as the NLD-NLS type model. The parallel configuration involves the MLFFNN and NARX-type RNN connected in parallel to each other, referred to as the NLS||NLD model. The hybrid series and parallel static-dynamic networks are shown in Fig. S1.

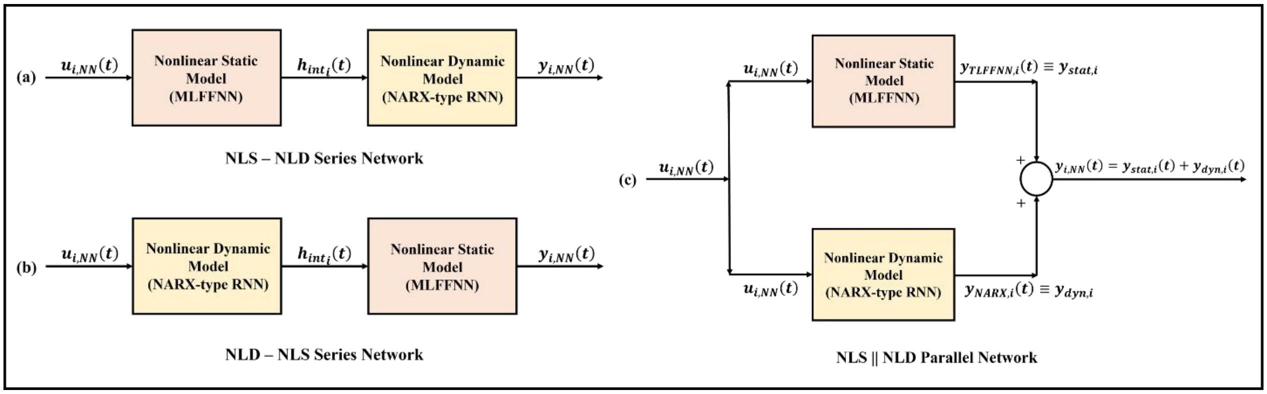

Fig. S1: Block-oriented architectures for (a) NLS – NLD (series), (b) NLD – NLS (series), and (c) NLS || NLD (parallel) types of hybrid static-dynamic networks

S.1.2. Training / Forward Problem Formulations for Hybrid Parallel and Series MECNNs

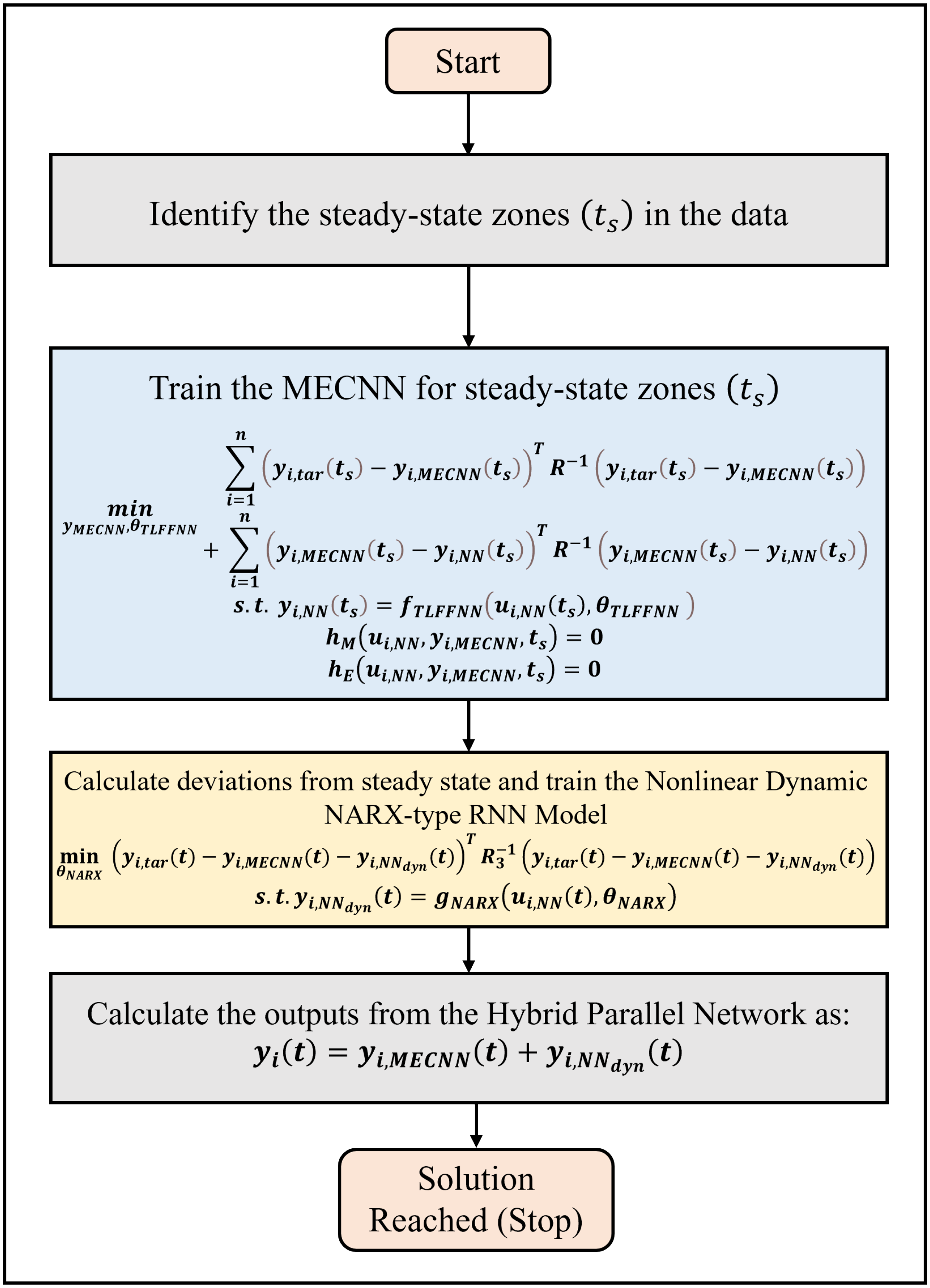

Fig. S2: Training Algorithm Flowchart of the Hybrid Parallel MECNN Model

S.2: Additional Details about the Case Study Examples under Consideration

This section consists of the schematics / system descriptions of some of the case studies considered in this work.

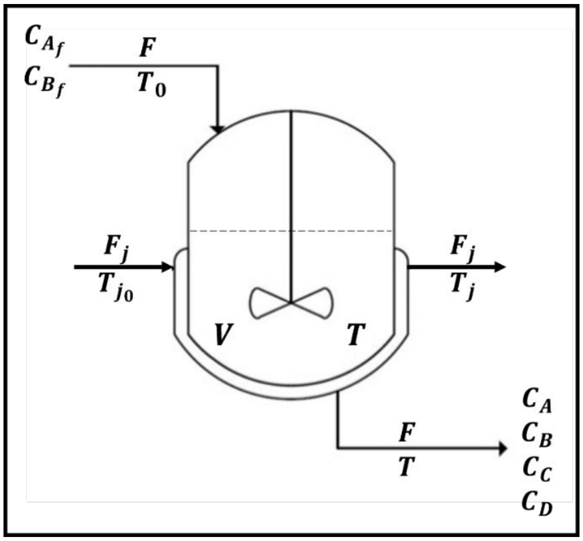

Fig. S3: Schematic of the Nonisothermal Van de Vusse Reactor for MECNN Implementation

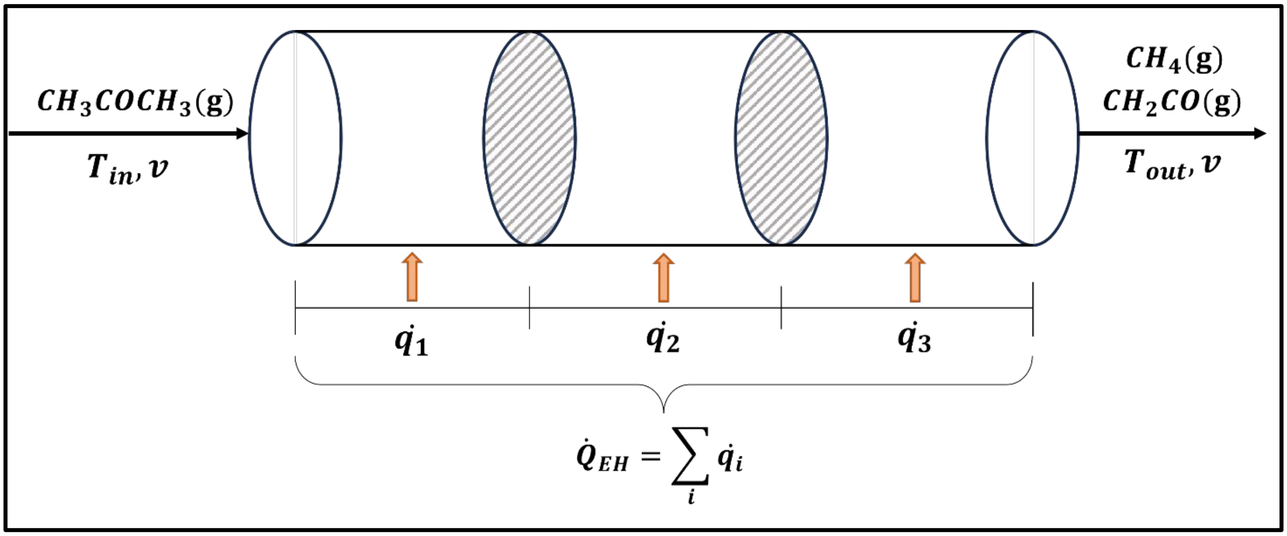

Fig. S4: Schematic of the Electrically Heater Tubular Reactor (for cracking of acetone) for MECNN Implementation

S.3: Additional Training / Simulation Results for Steady-State MECNNs

This section consists of additional training and simulation (forward problem) results obtained from steady-state implementations of MECNNs while modeling the three case study examples under different types of error characterizations. The results corresponding to both lumped and distributed models of the superheater / reheater system have been collectively compiled under Case Study 1.

In this section,

- **Figs. S5** through **S21** include additional steady-state results for Case Study 1.
- **Figs. S22** through **S31** include additional steady-state results for Case Study 2.
- **Figs. S32** through **S41** include additional steady-state results for Case Study 3.

S.3.1. Case Study 1: Adiabatic Superheater / Reheater System

In this section, **Figs. S5 through S11** show additional steady-state training / simulation results for the **lumped parameter model** of the superheater / reheater system, subject to the three different types of noise characterizations considered in this work. Similarly, Figs. **S12 through S21** show additional steady-state results for the **distributed** superheater / reheater system under different noise characterizations, in the same sequence as discussed in the paper.

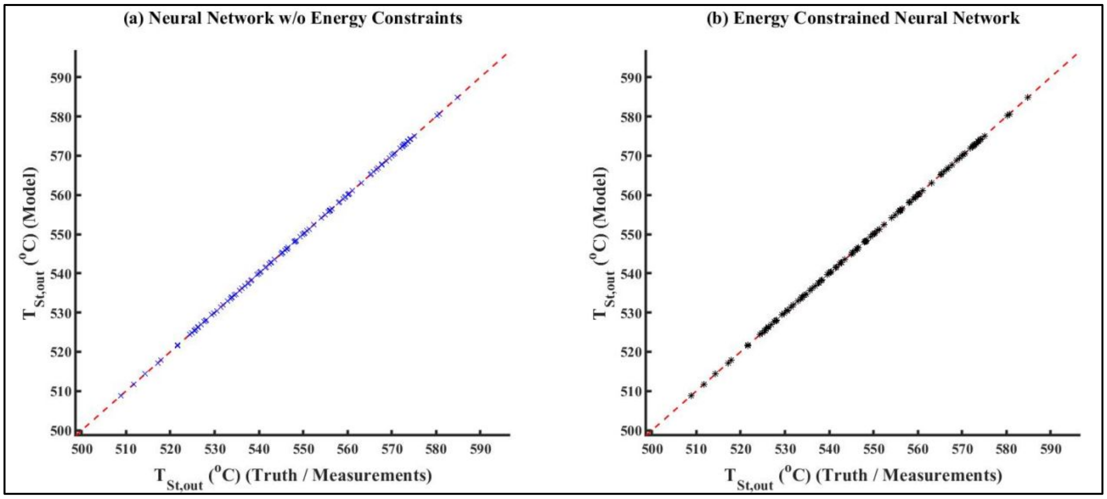

Fig. S5: Comparison of results between ECNN and NN w/o energy constraints for the simulation data of $T_{St,out}$ (noise in the measurement data represented by Eq. (3))

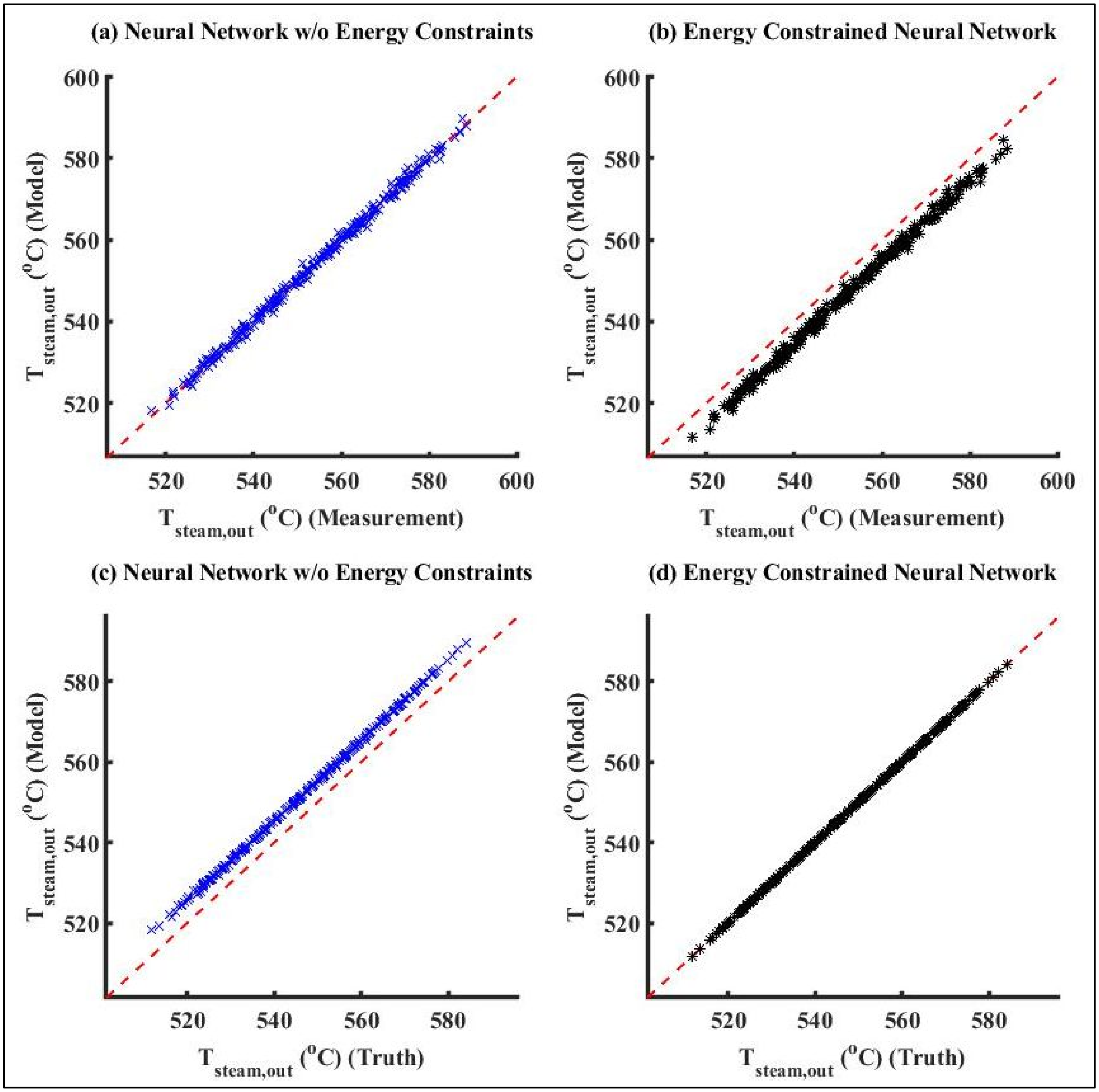

Fig. S6: Comparison of results between ECNN and NN w/o energy constraints for the training data of $T_{St,out}$ (noise in the measurement data represented by Eq. (4))

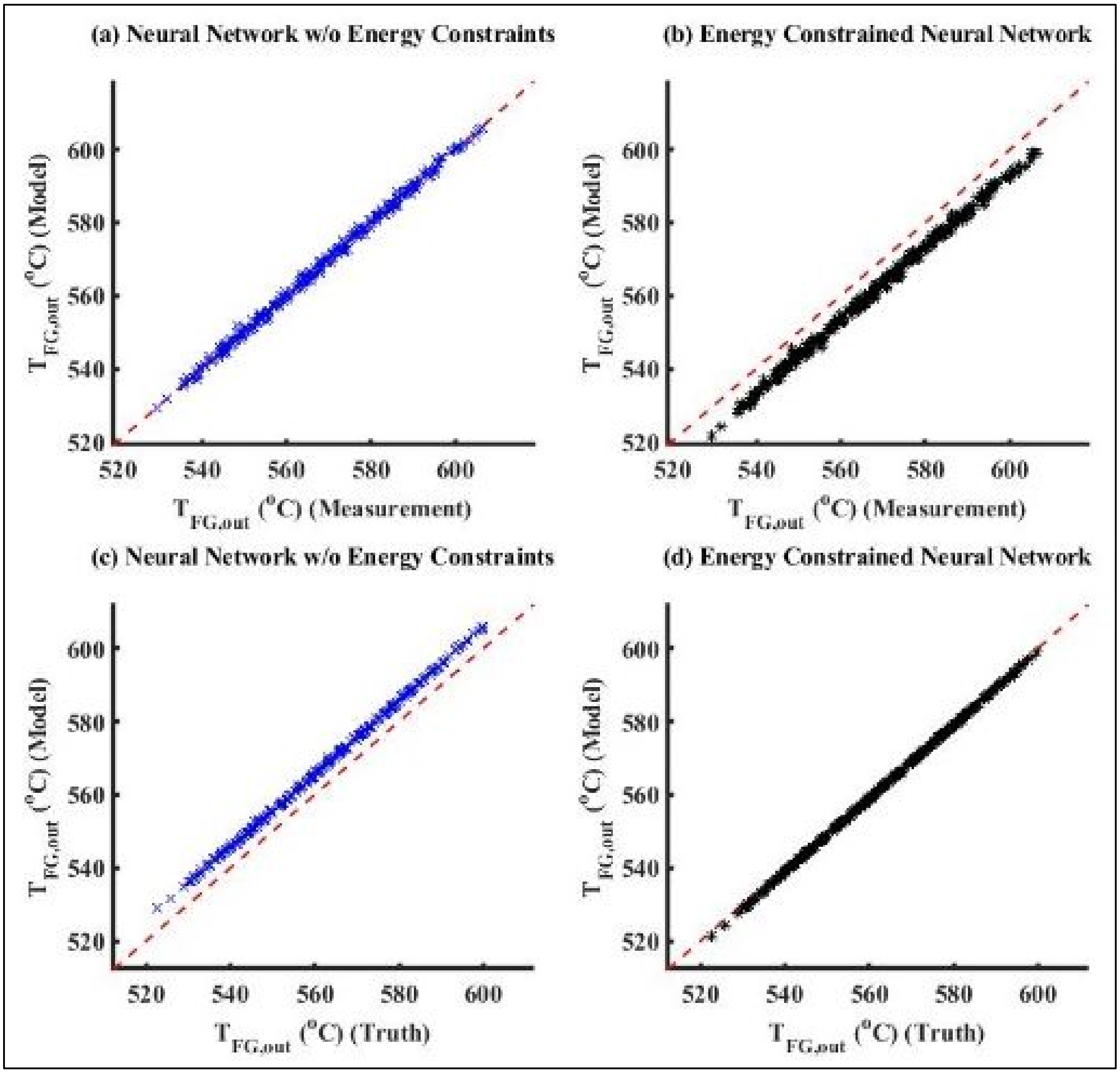

Fig. S7: Comparison of results between ECNN and NN w/o energy constraints for the training data of $T_{FG,out}$ (noise in the measurement data represented by Eq. (4))

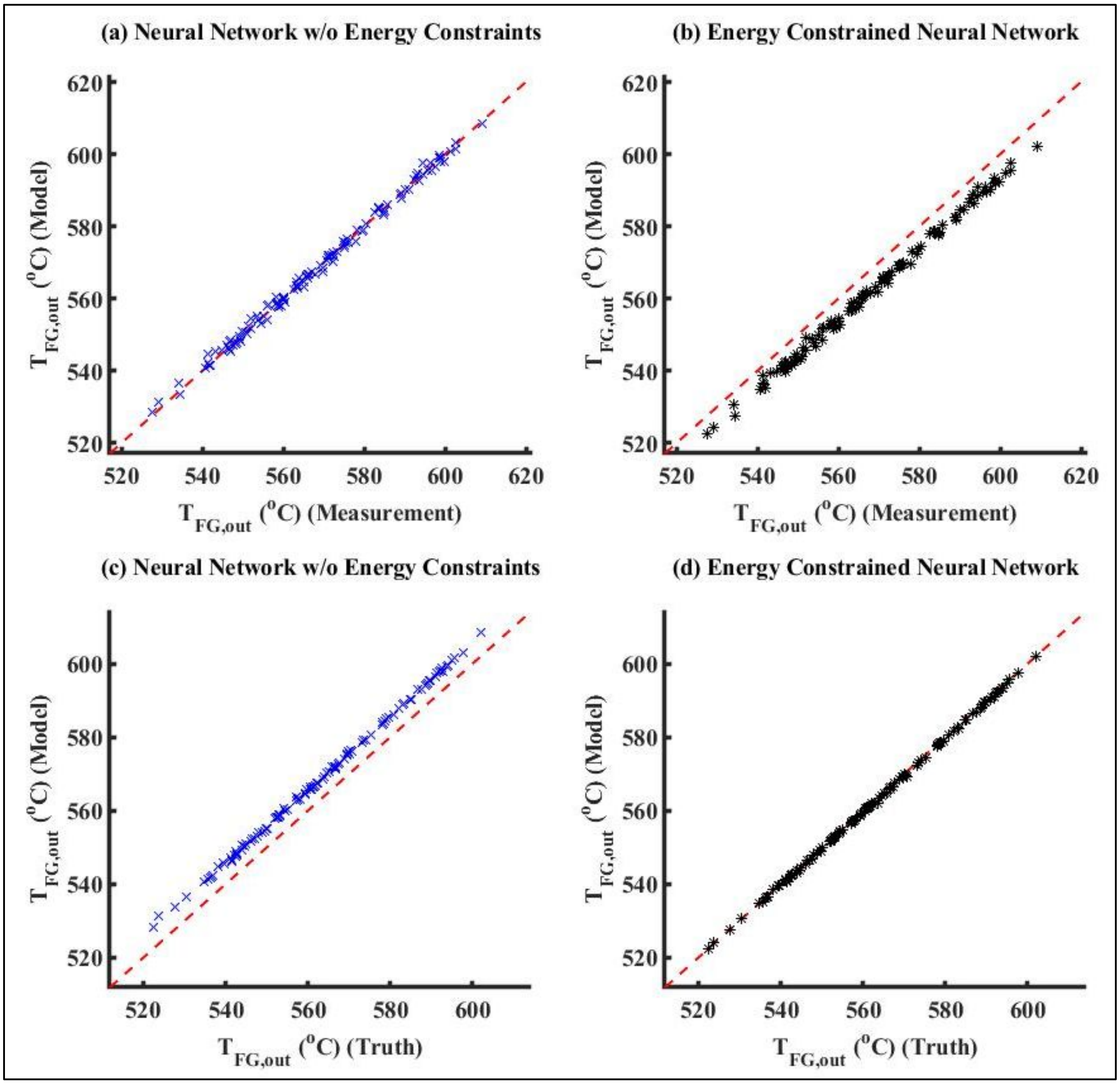

Fig. S8: Comparison of results between ECNN and NN w/o energy constraints for the simulation data of $T_{FG,out}$ (noise in the measurement data represented by Eq. (4))

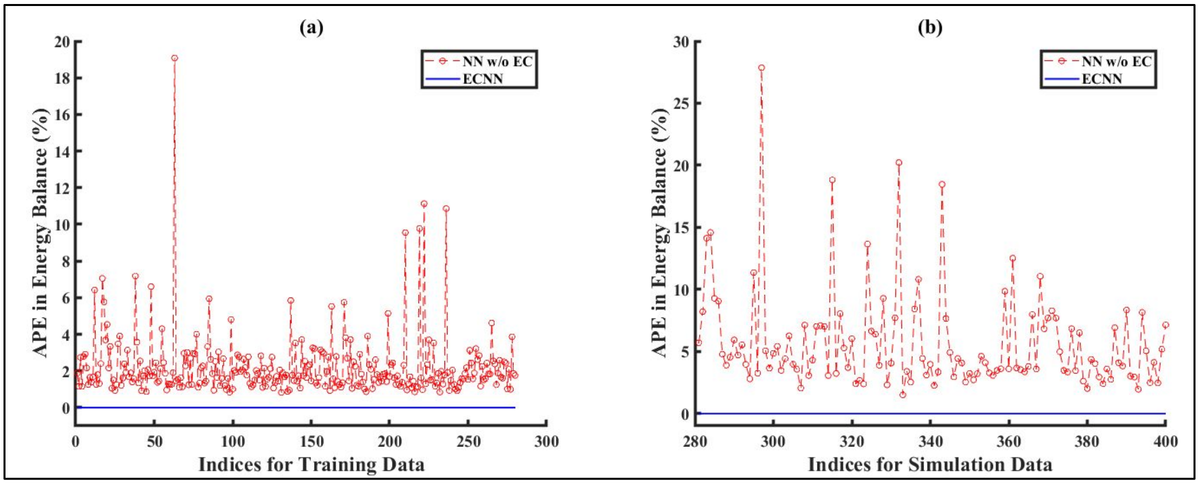

Fig. S9: Comparison of error between ECNN and NN w/o energy constraints for energy balance during (a) training and (b) simulation (noise in the measurement data represented by Eq. (4))

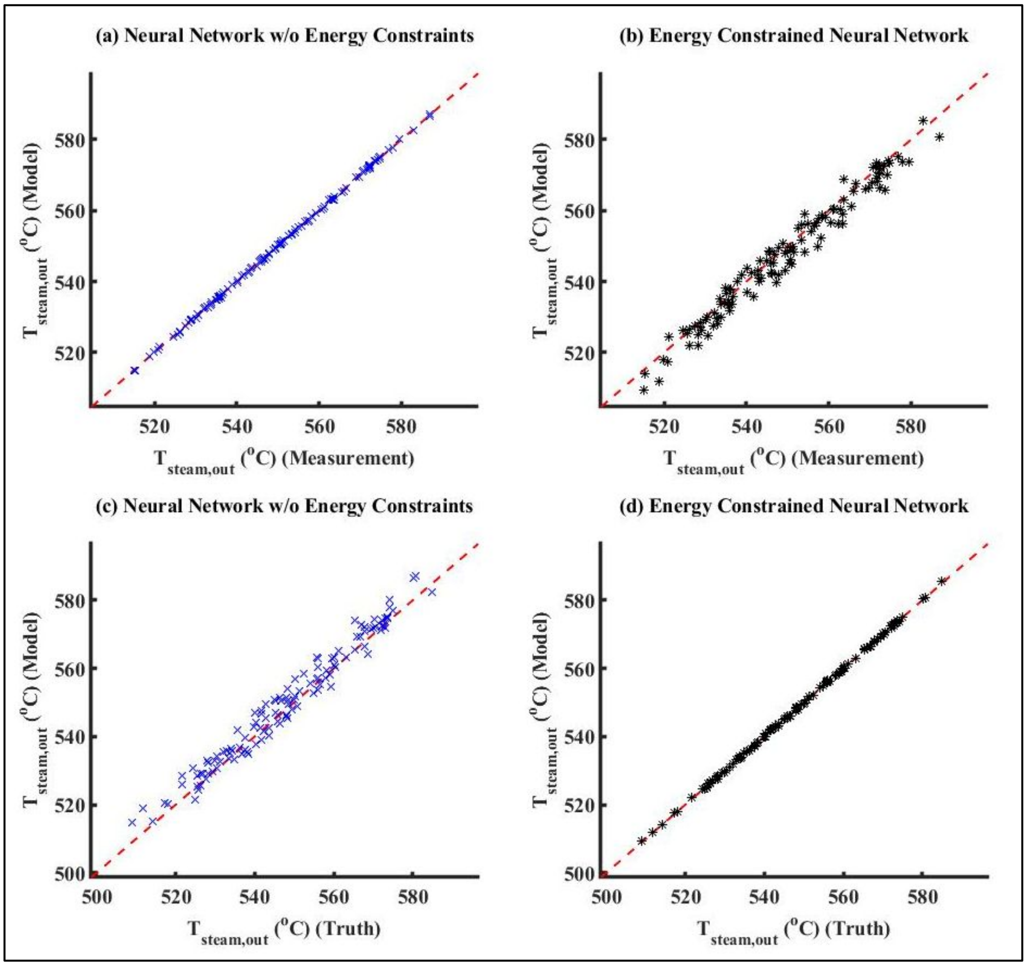

Fig. S10: Comparison of results between ECNN and NN w/o energy constraints for the simulation data of $T_{St,out}$ (noise in the measurement data represented by Eq. (5))

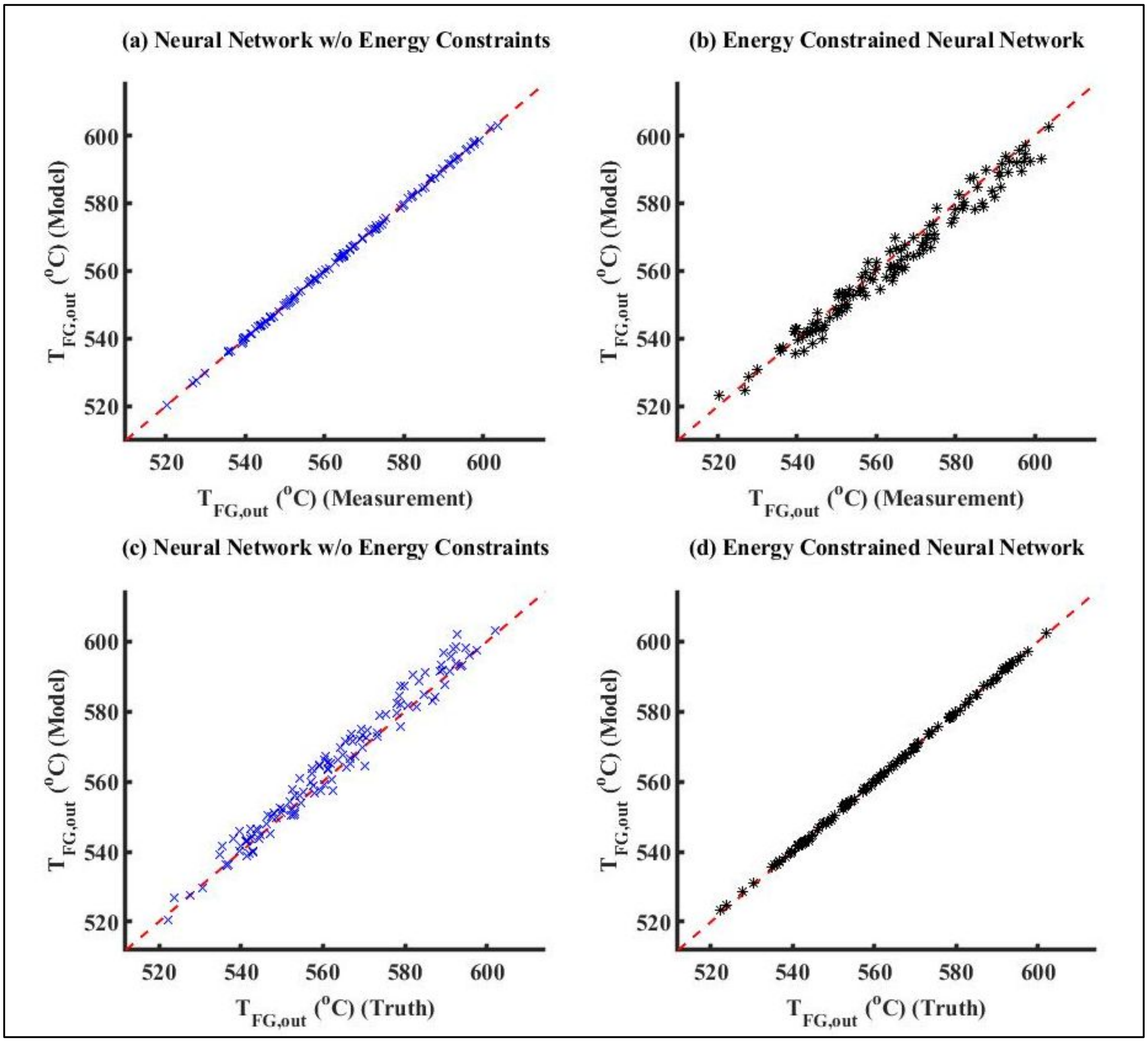

Fig. S11: Comparison of results between ECNN and NN w/o energy constraints for the simulation data of $T_{FG,out}$ (noise in the measurement data represented by Eq. (5))

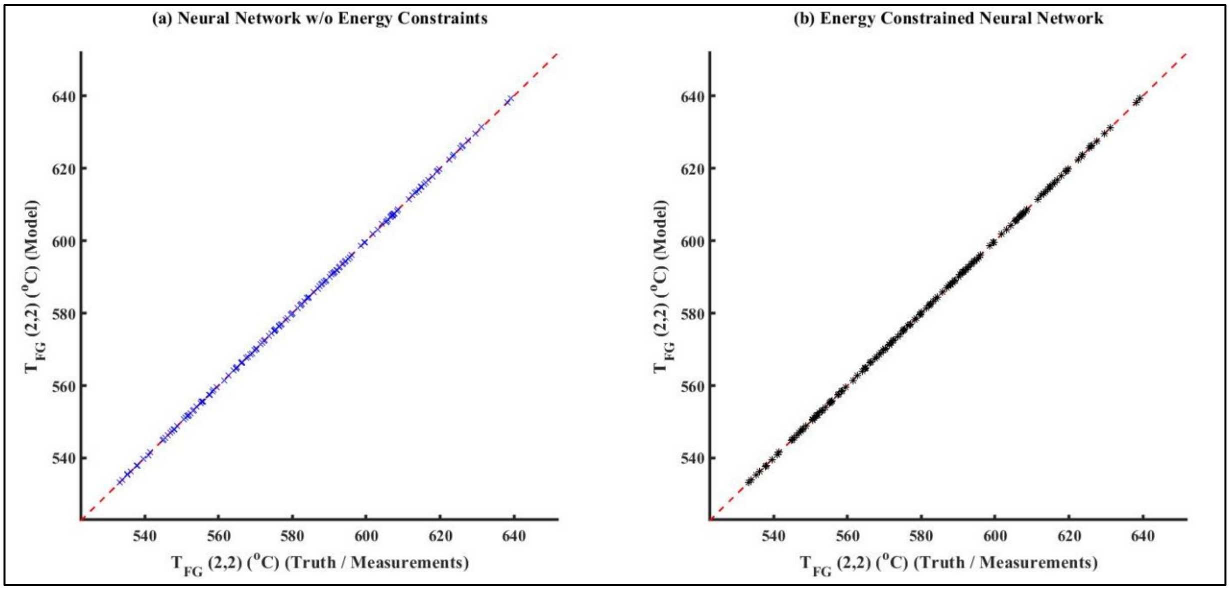

Fig. S12: Comparison of results between ECNN and NN w/o energy constraints for the training data of $T_{FG,out}$ at $(2,2)$ (noise in the measurement data represented by Eq. (3))

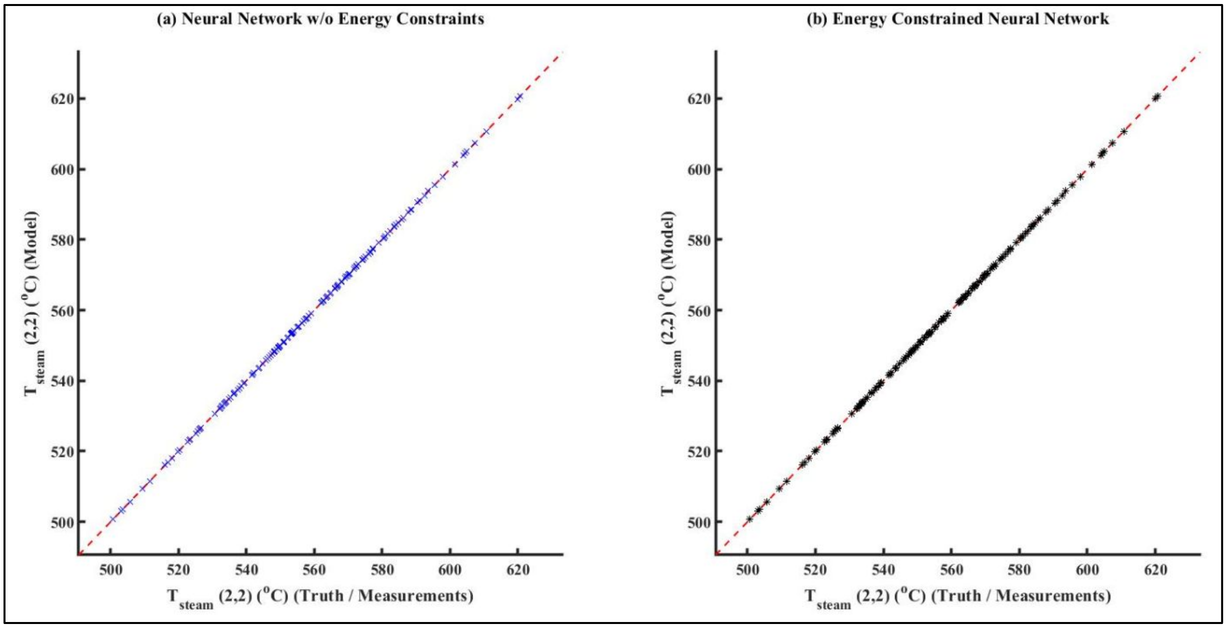

Fig. S13: Comparison of results between ECNN and NN w/o energy constraints for the training data of $T_{St,out}$ at $(2,2)$ (noise in the measurement data represented by Eq. (3))

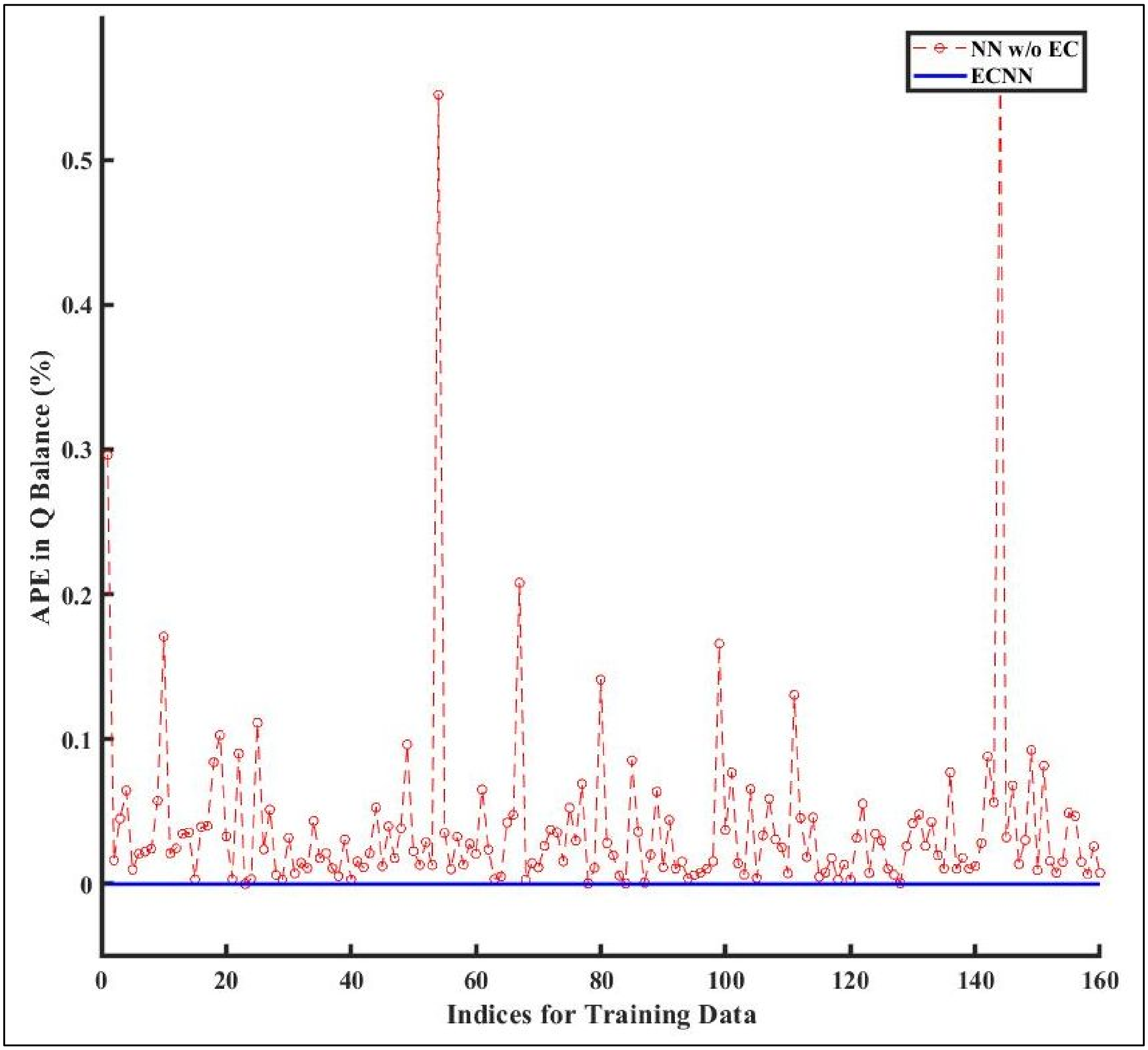

Fig. S14: Comparison of error between ECNN and NN w/o energy constraints for energy balance at system boundary during training (noise in the measurement data represented by Eq. (3))

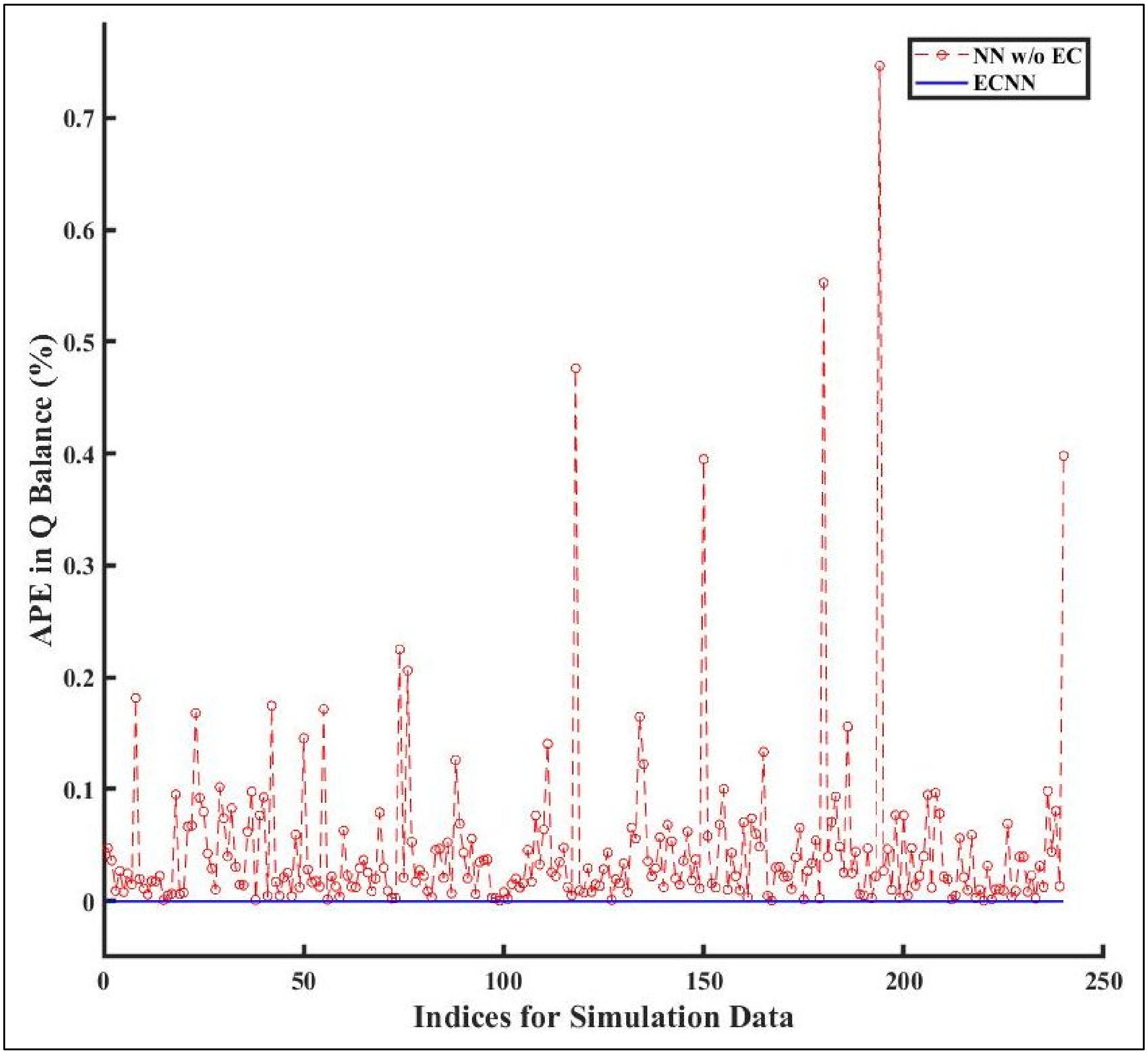

Fig. S15: Comparison of error between ECNN and NN w/o energy constraints for energy balance at system boundary during simulation (noise in the measurement data represented by Eq. (3))

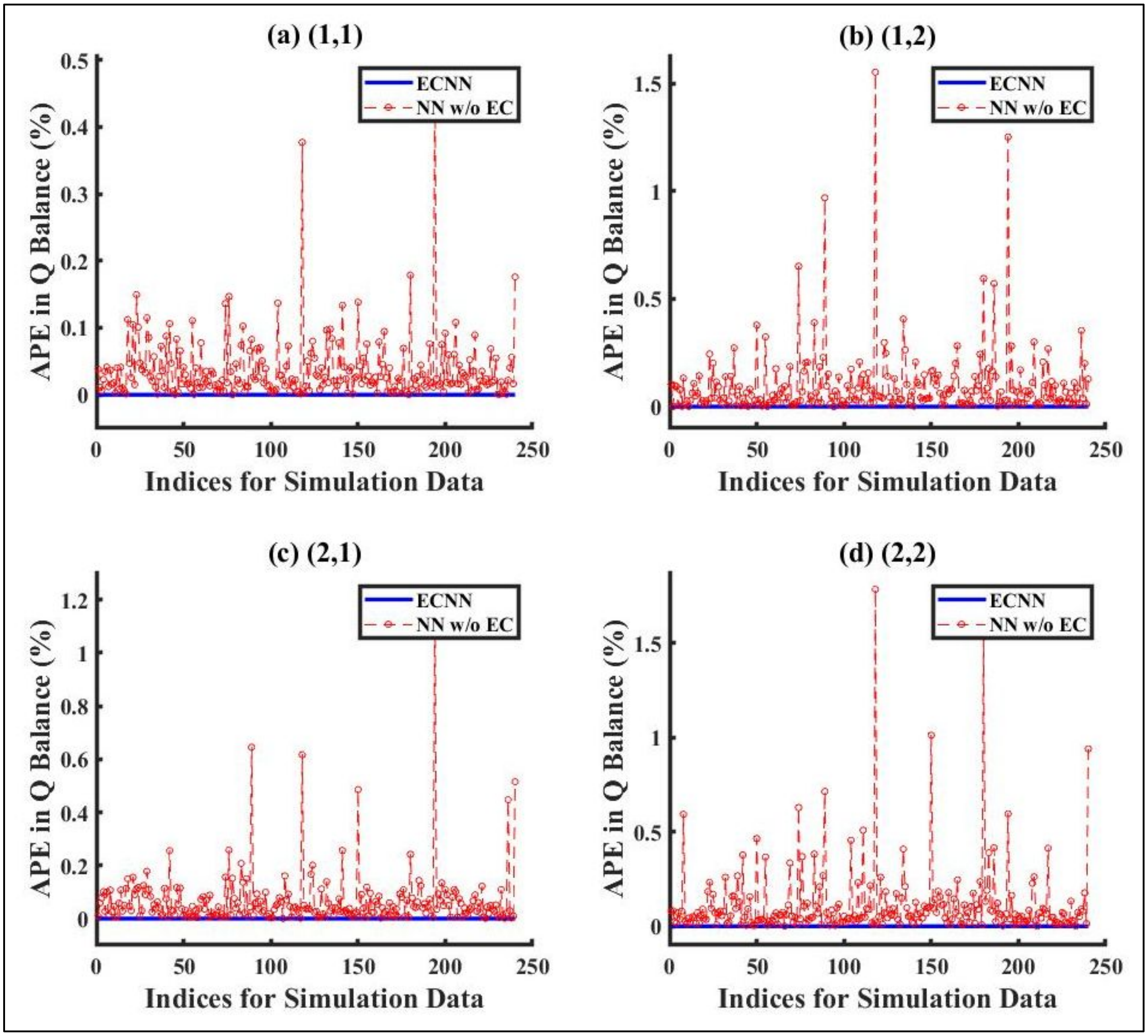

Fig. S16: Comparison of error between ECNN and NN w/o energy constraints for energy balance at individual grids during simulation (noise in the measurement data represented by Eq. (3))

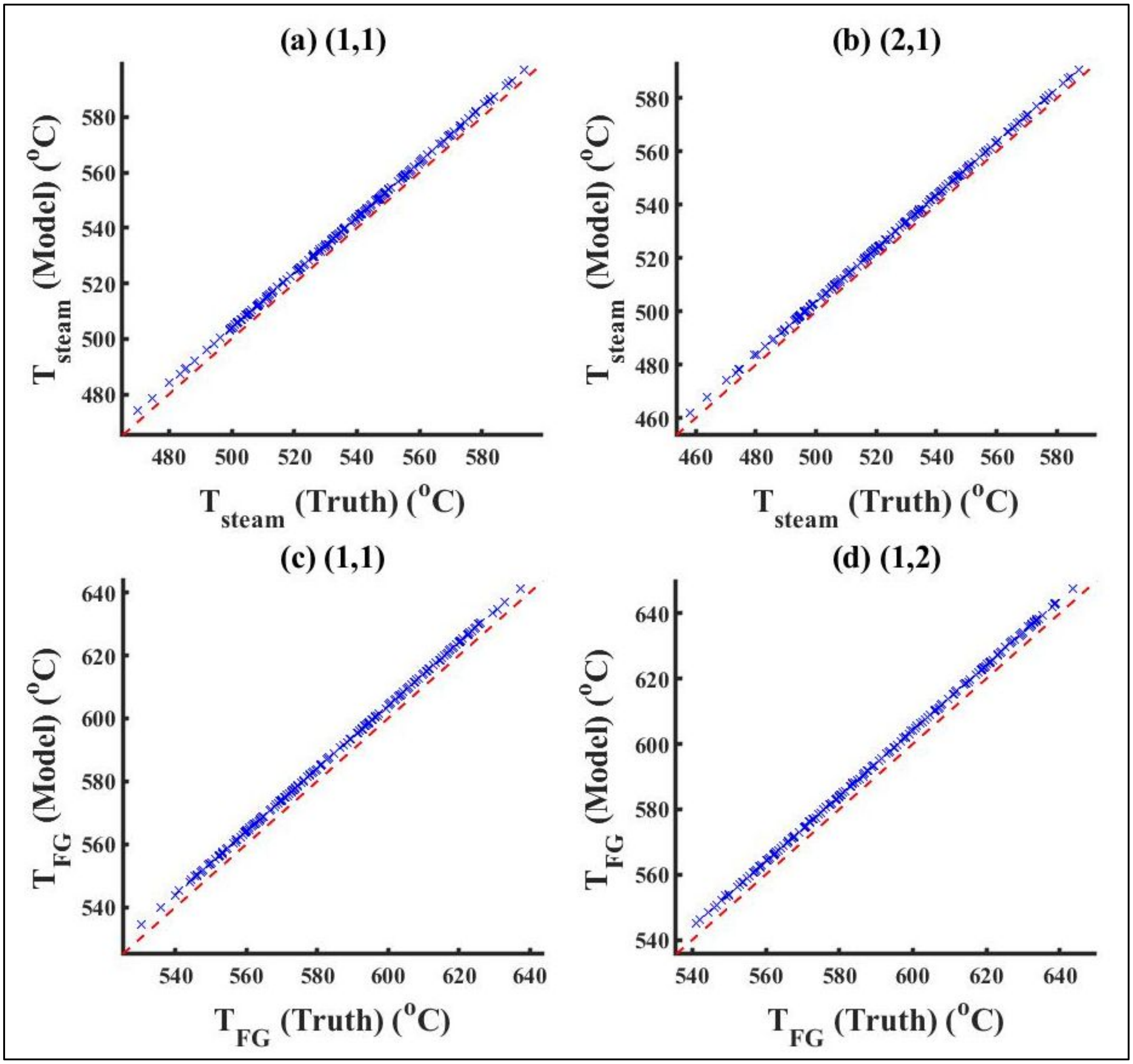

Fig. S17: Results from NN w/o energy constraints for the simulation data of $T_{St,out}$ and $T_{FG,out}$ at intermediate discretization grids (noise in the measurement data represented by Eq. (4))

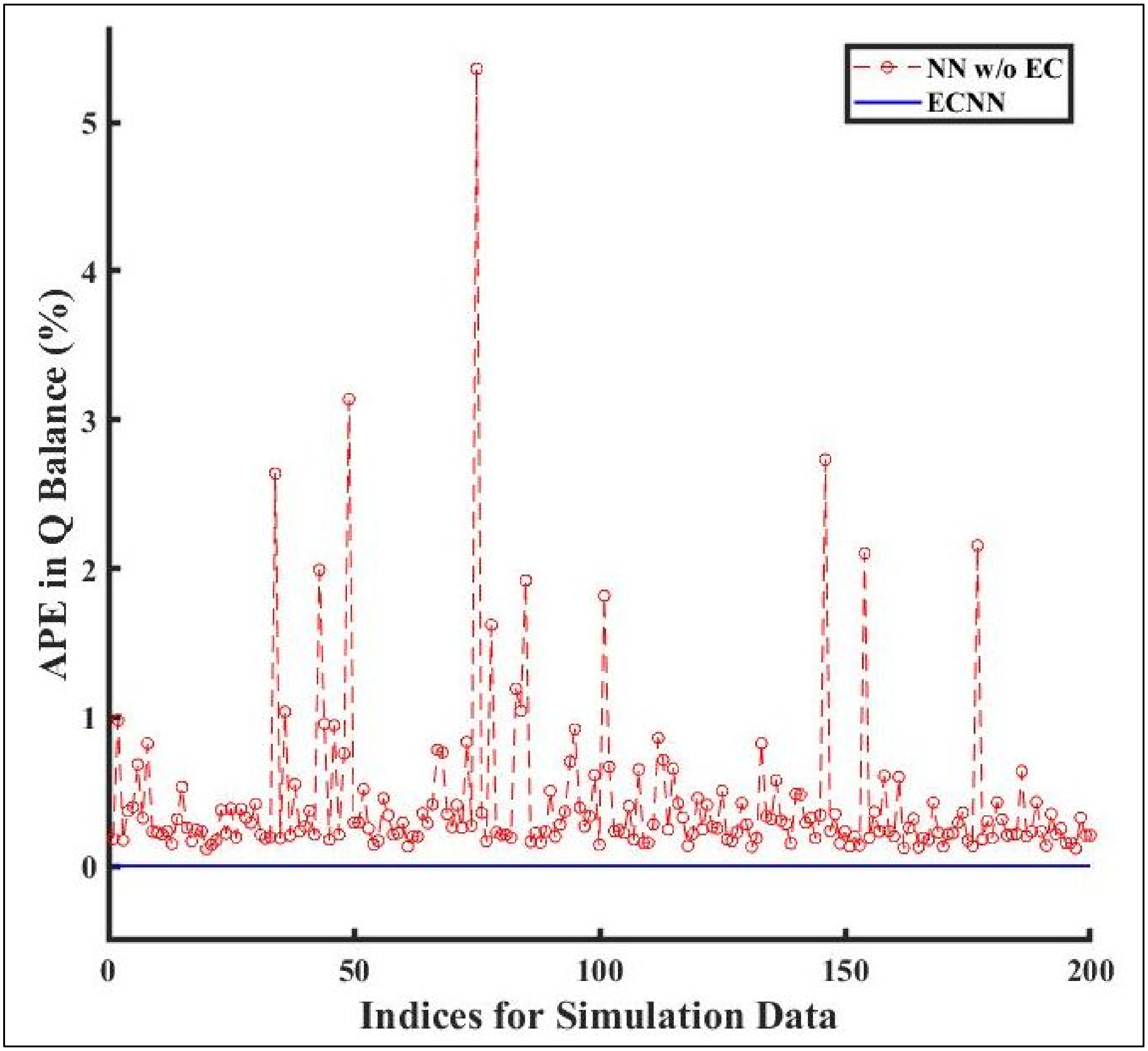

Fig. S18: Comparison of error between ECNN and NN w/o energy constraints for energy balance at system boundary during simulation (noise in the measurement data represented by Eq. (4))

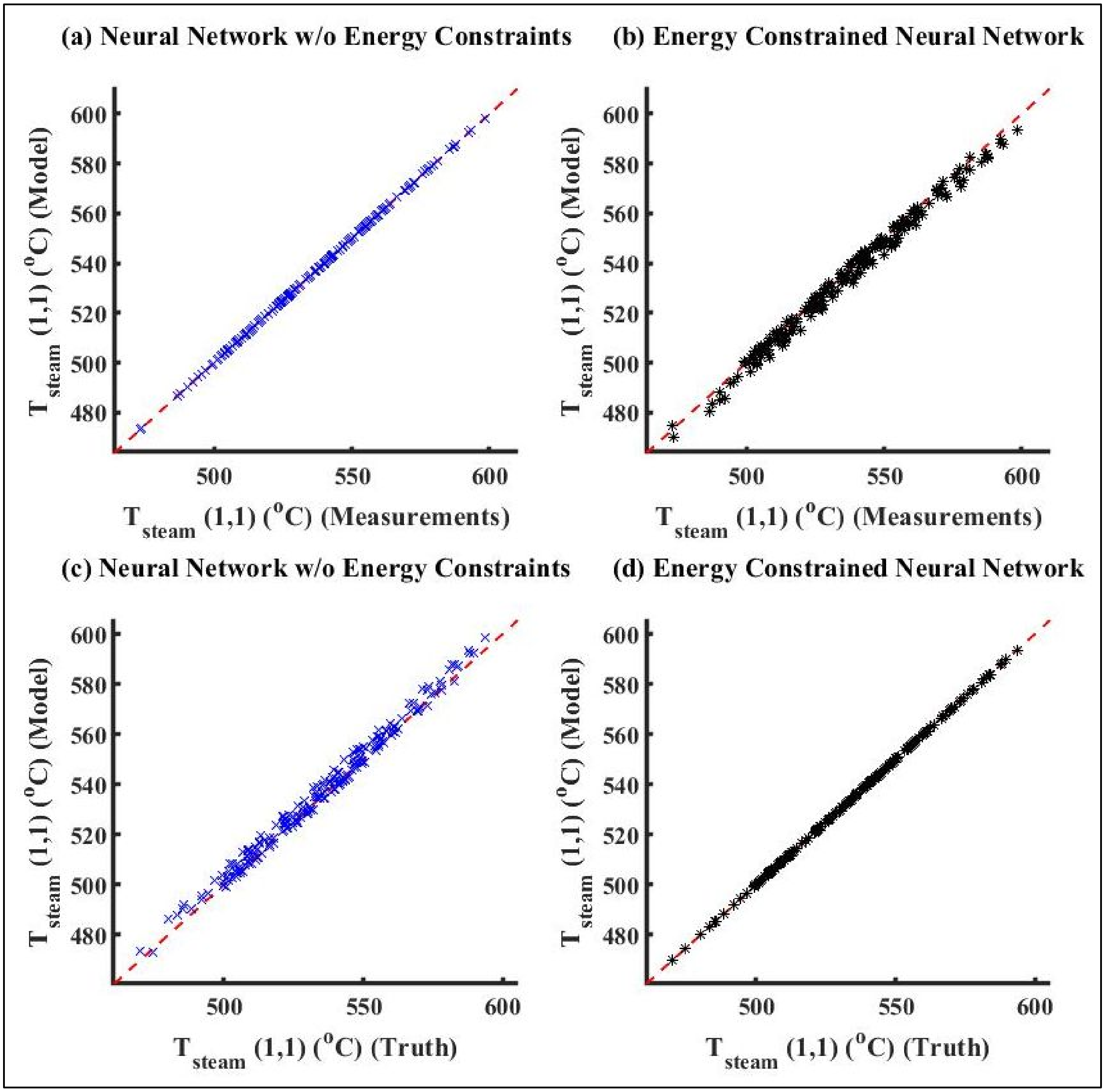

Fig. S19: Comparison of results between ECNN and NN w/o energy constraints for the simulation data of $T_{St,out}$ at grid (1,1) (noise in the measurement data represented by Eq. (5))

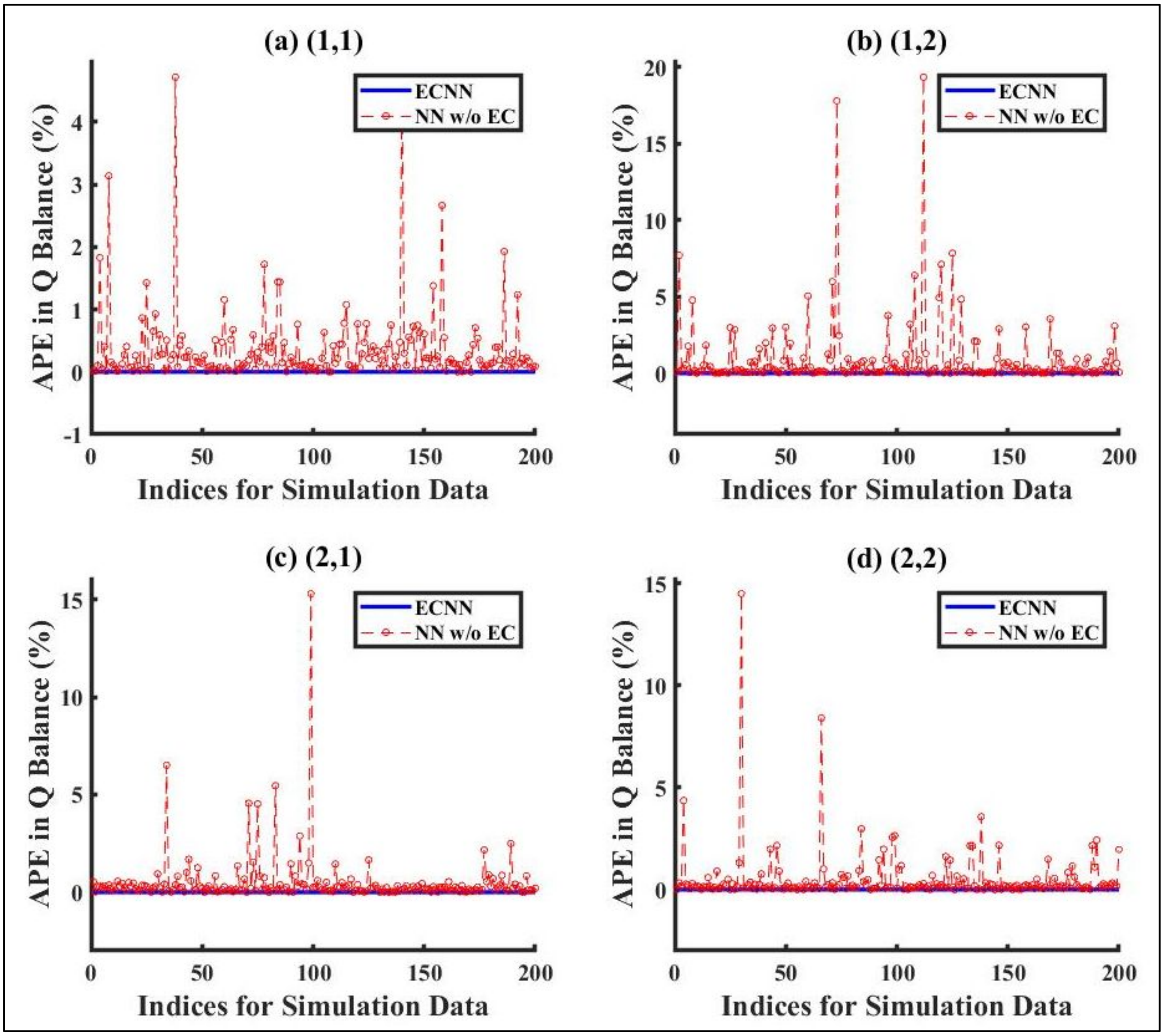

Fig. S20: Comparison of error between ECNN and NN w/o energy constraints for energy balance at intermediate grids during simulation (noise in the measurement data represented by Eq. (5))

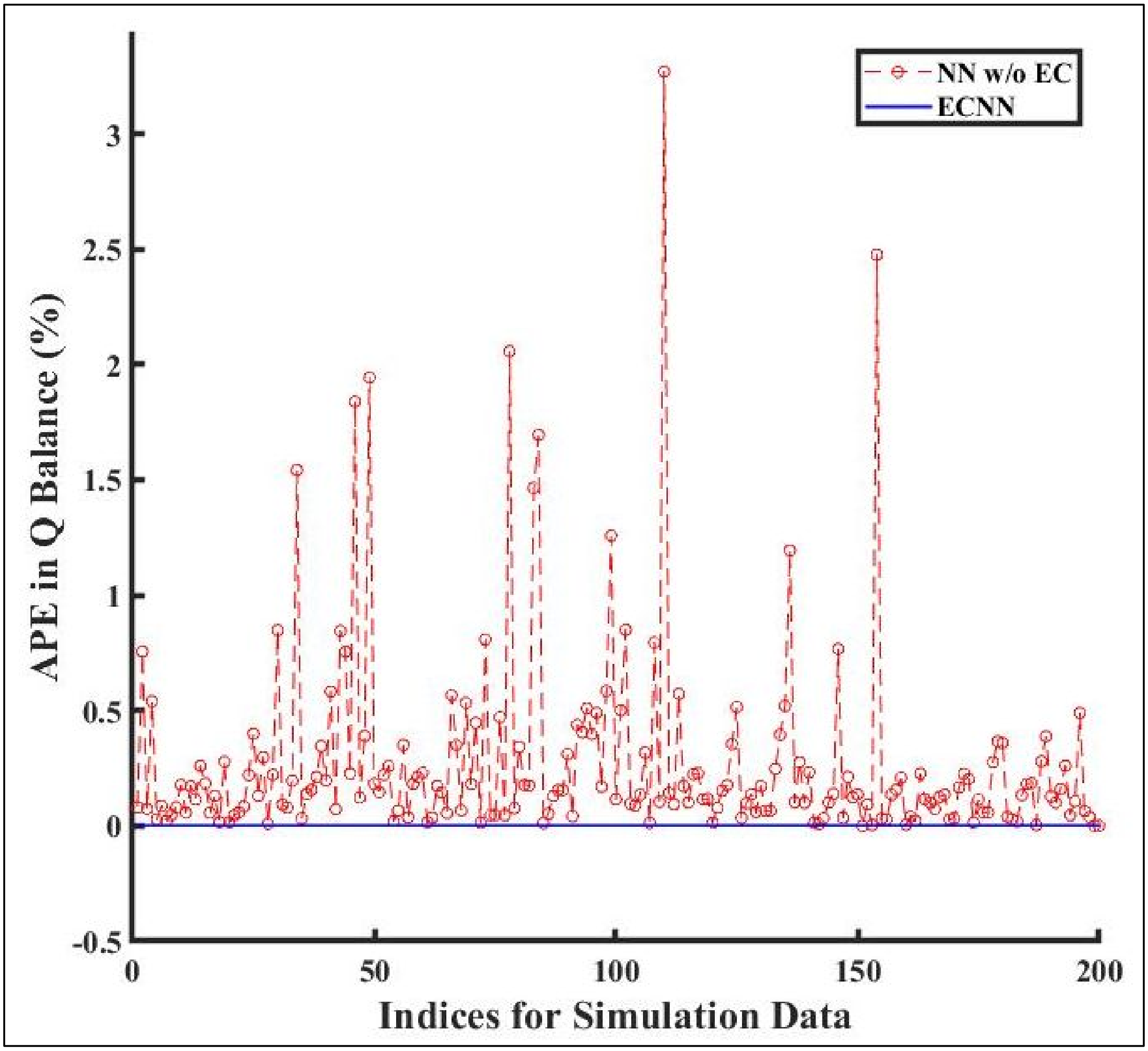

Fig. S21: Comparison of error between ECNN and NN w/o energy constraints for energy balance at system boundary during simulation (noise in the measurement data represented by Eq. (5))

S.3.2. Case Study 2: Non-Isothermal Van de Vusse Reactor System

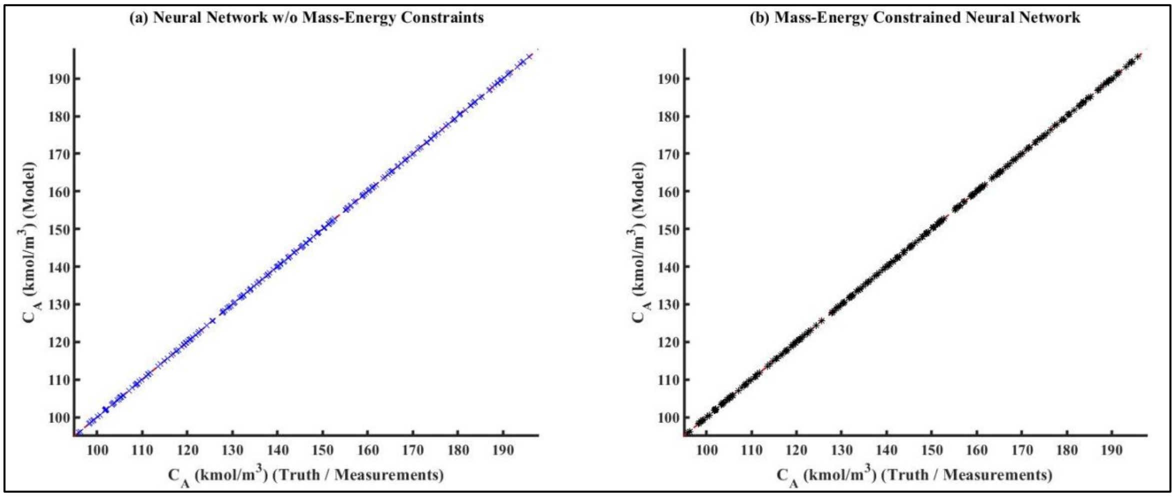

Fig. S22: Comparison of results between MECNN and NN w/o mass-energy constraints for the training data of C_A (noise in the measurement data represented by Eq. (3))

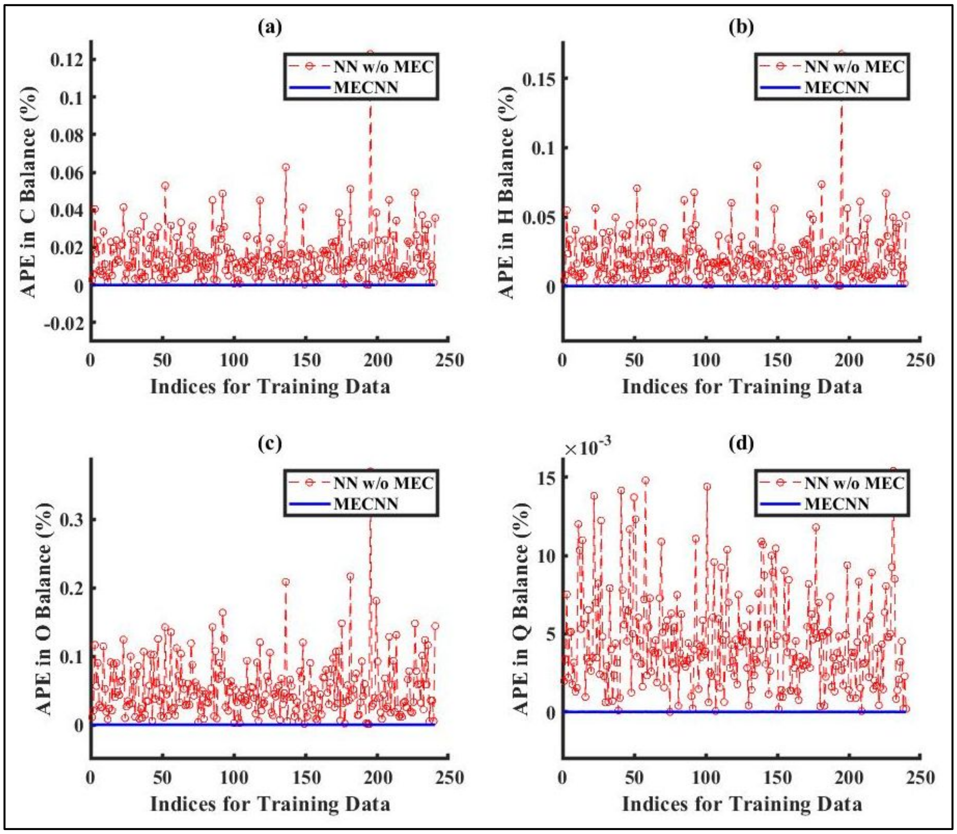

Fig. S23: Comparison between MECNN and NN w/o mass-energy constraints in terms of violating (a) carbon (C), (b) hydrogen (H), (c) oxygen (O) and (d) energy (Q) balance constraints during training (noise in the measurement data represented by Eq. (3))

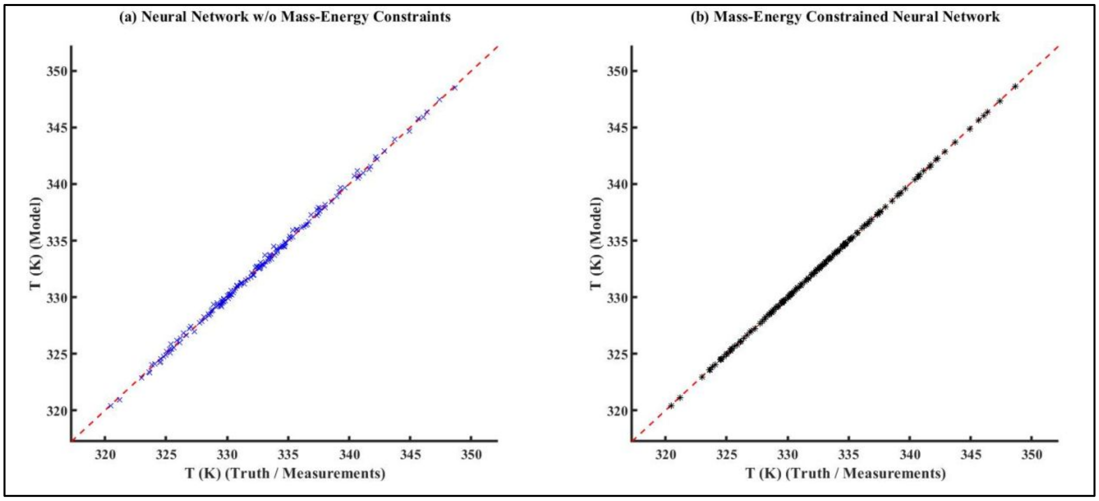

Fig. S24: Comparison of results between MECNN and NN w/o mass-energy constraints for the simulation data of T (noise in the measurement data represented by Eq. (3))

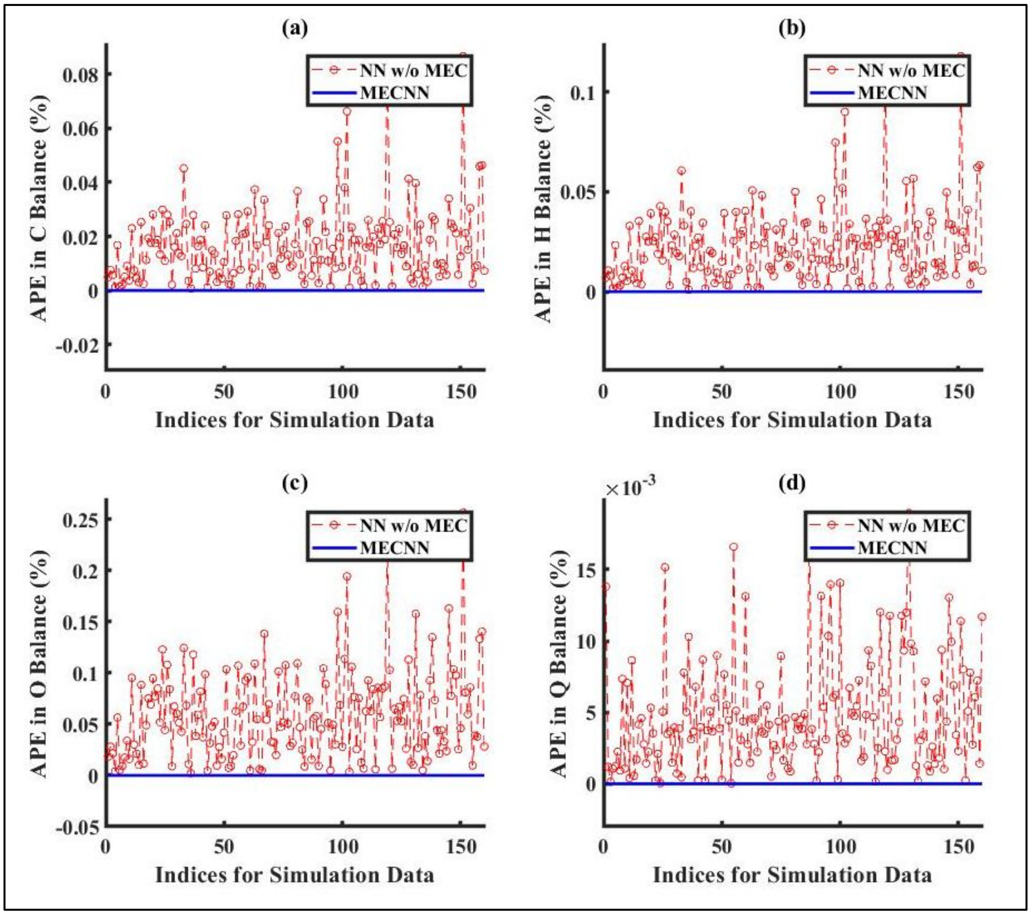

Fig. S25: Comparison between MECNN and NN w/o mass-energy constraints in terms of violating (a) carbon (C), (b) hydrogen (H), (c) oxygen (O) and (d) energy (Q) balance constraints during simulation (noise in the measurement data represented by Eq. (3))

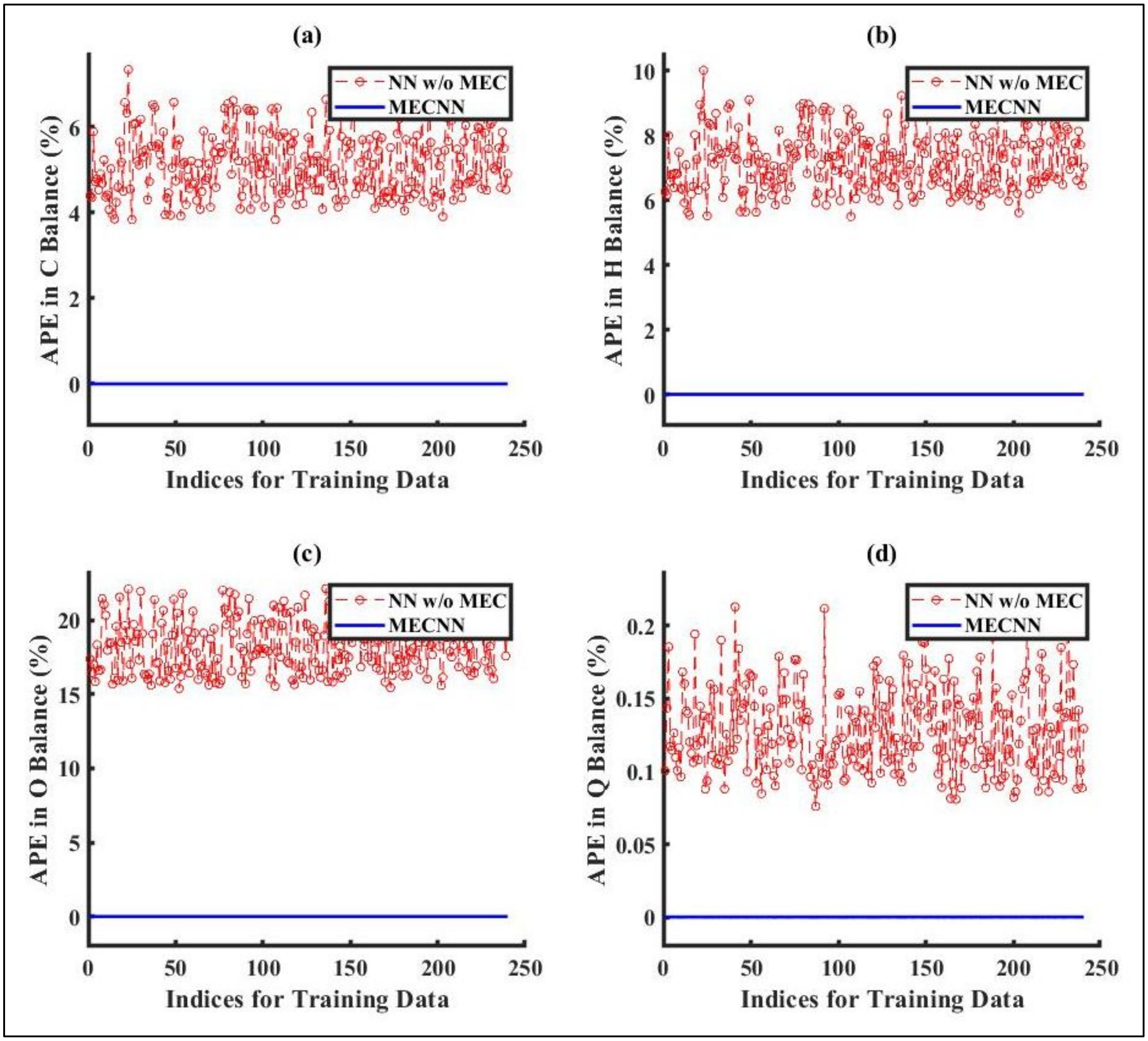

Fig. S26: Comparison between MECNN and NN w/o mass-energy constraints in terms of violating (a) carbon (C), (b) hydrogen (H), (c) oxygen (O) and (d) energy (Q) balance constraints during training (noise in the measurement data represented by Eq. (4))

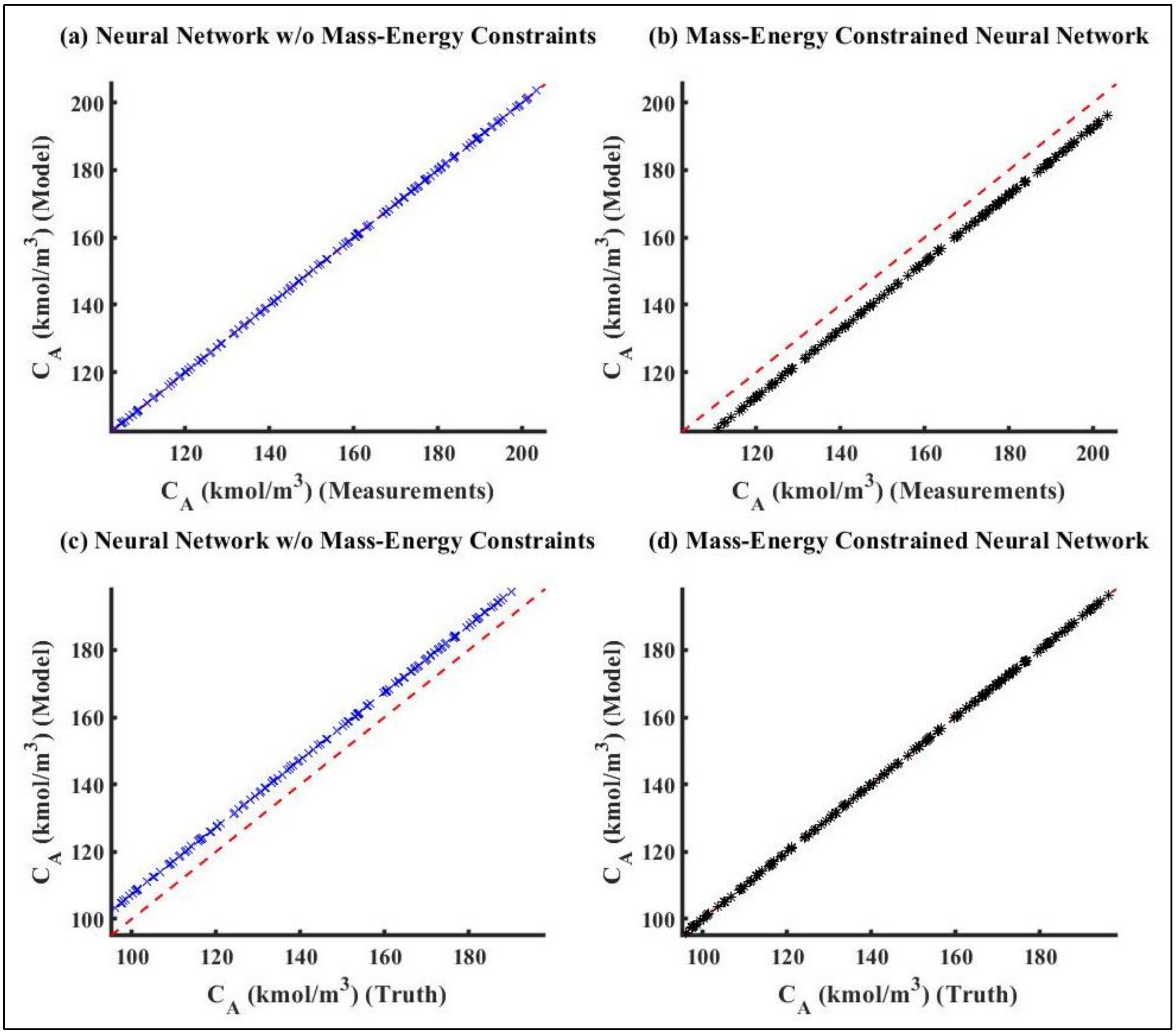

Fig. S27: Comparison of results between MECNN and NN w/o mass-energy constraints for the simulation data of outlet concentration of A (C_A) (noise in the measurement data represented by Eq. (4))

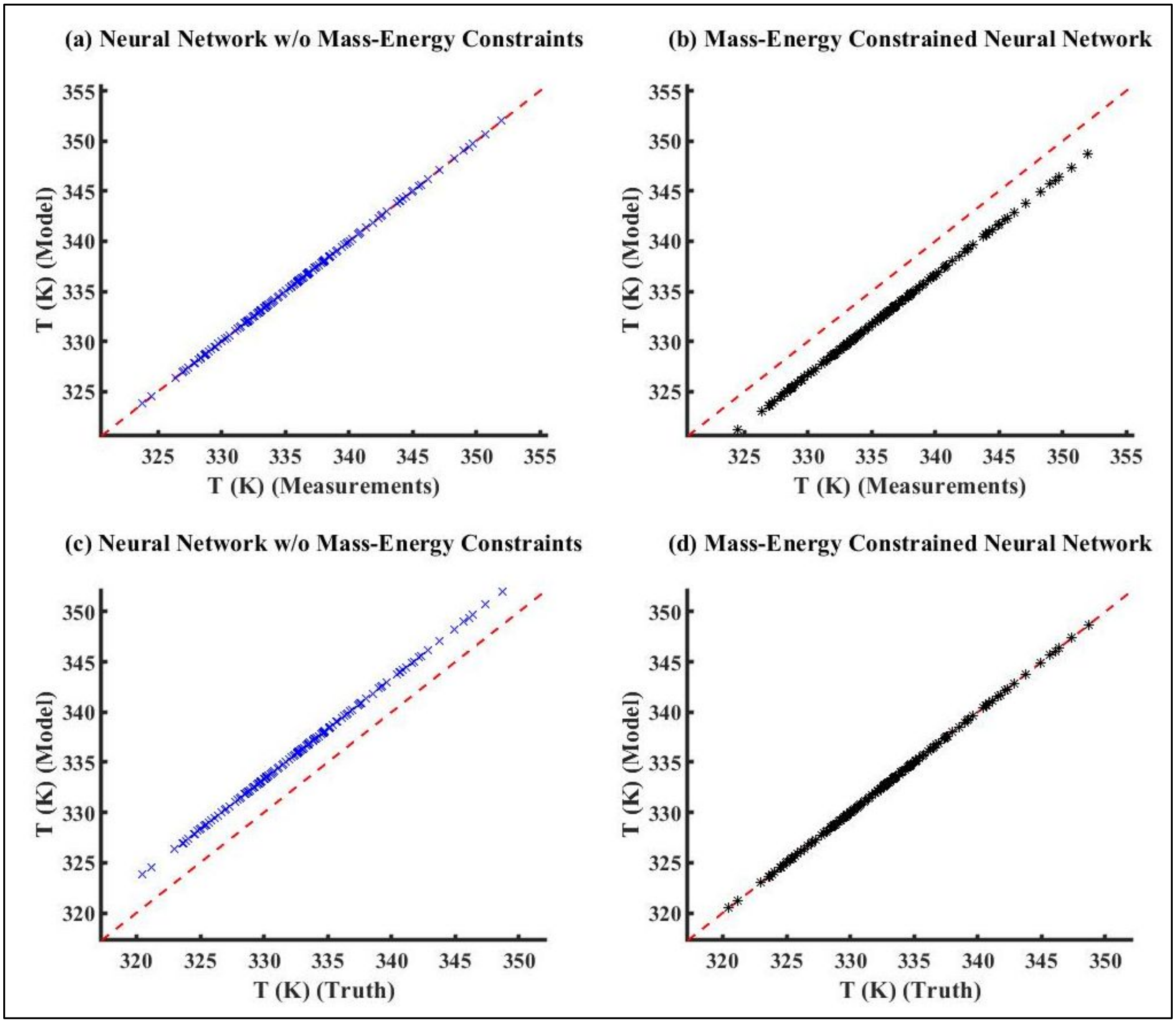

Fig. S28: Comparison of results between MECNN and NN w/o mass-energy constraints for the simulation data of outlet reactor temperature (T) (noise in the measurement data represented by Eq. (4))

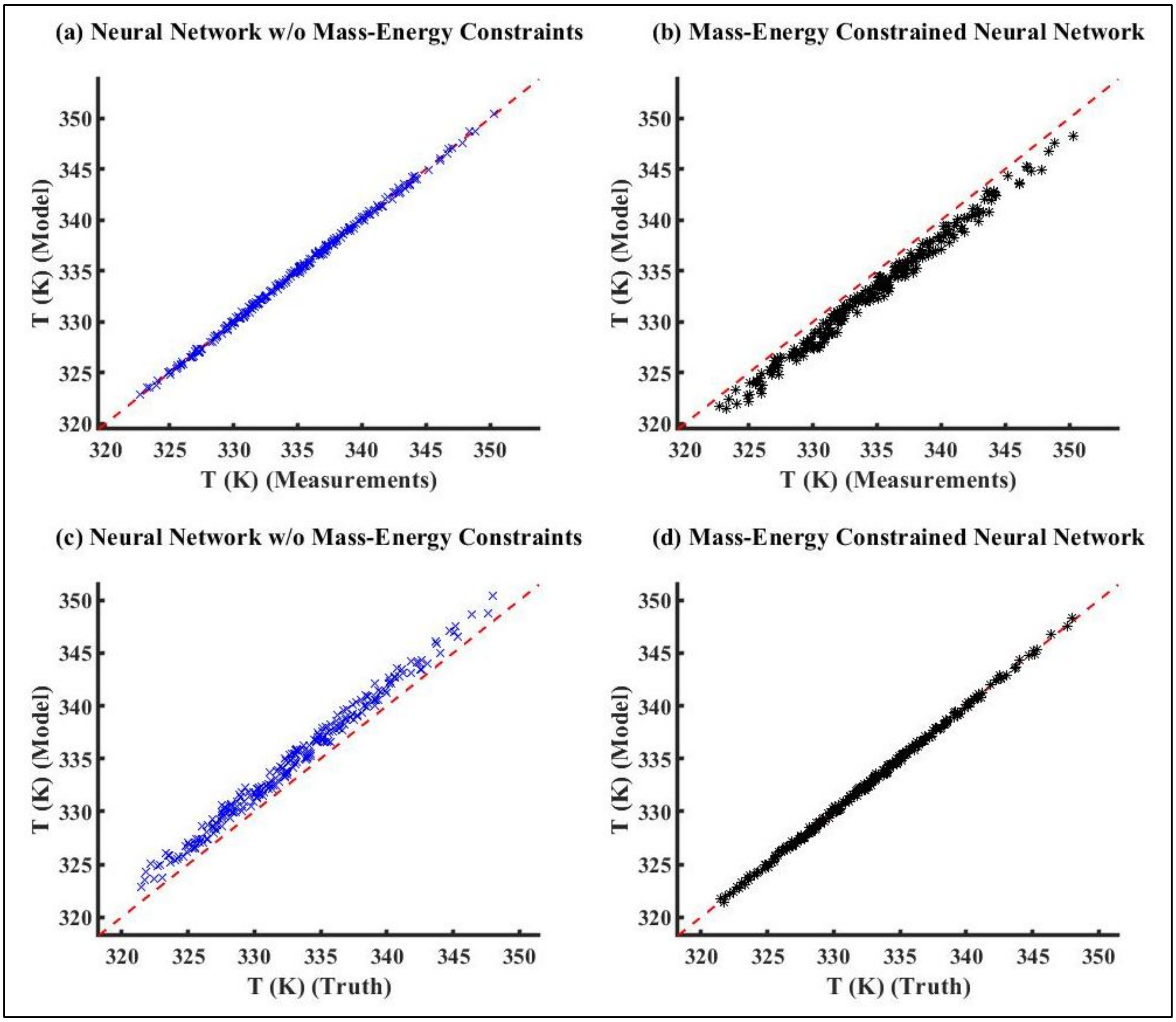

Fig. S29: Comparison of results between MECNN and NN w/o mass-energy constraints for the training data of outlet reactor temperature (T) (noise in the measurement data represented by Eq. (5))

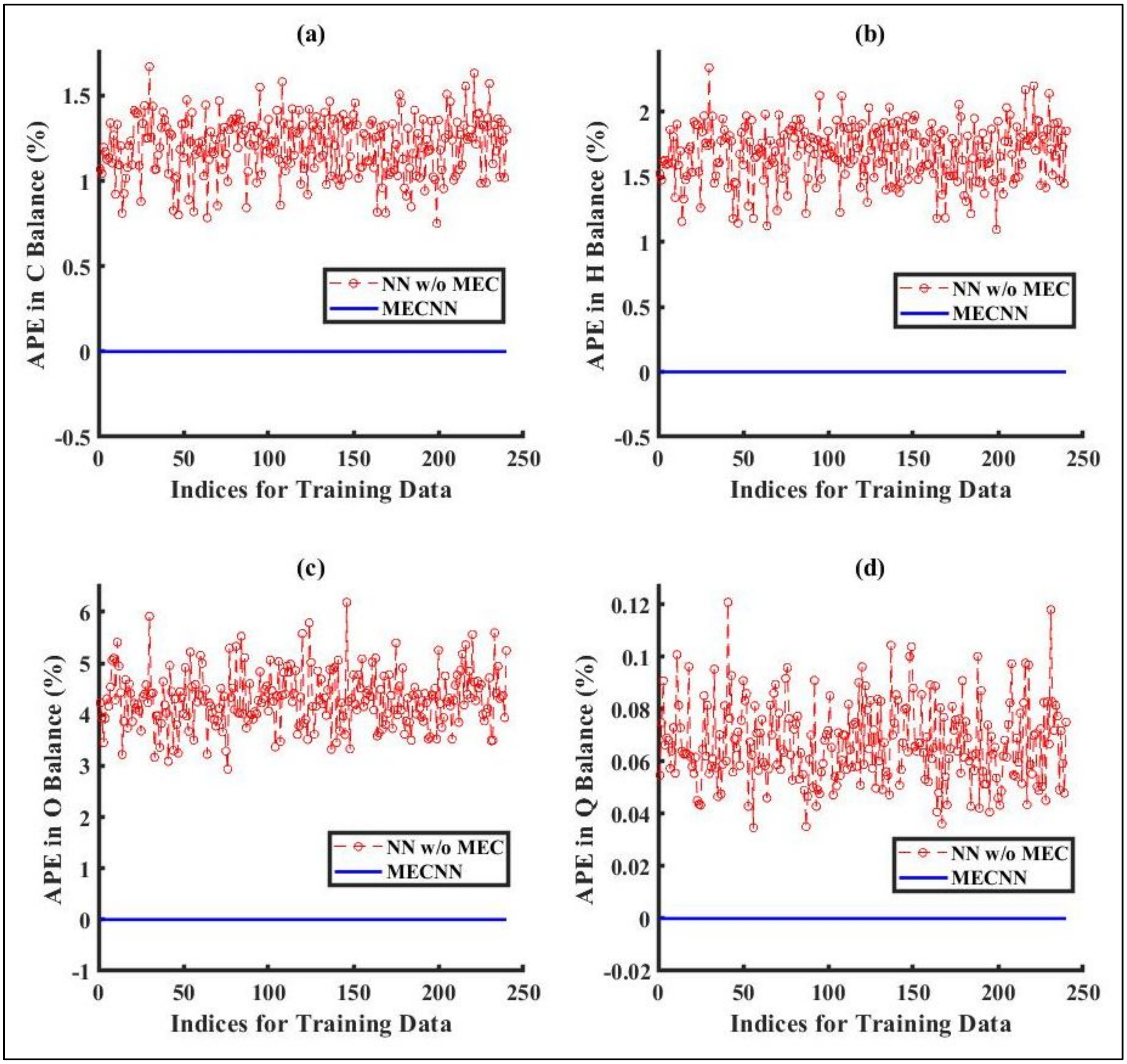

Fig. S30: Comparison between MECNN and NN w/o mass-energy constraints in terms of violating (a) carbon (C), (b) hydrogen (H), (c) oxygen (O) and (d) energy (Q) balance constraints during training (noise in the measurement data represented by Eq. (5))

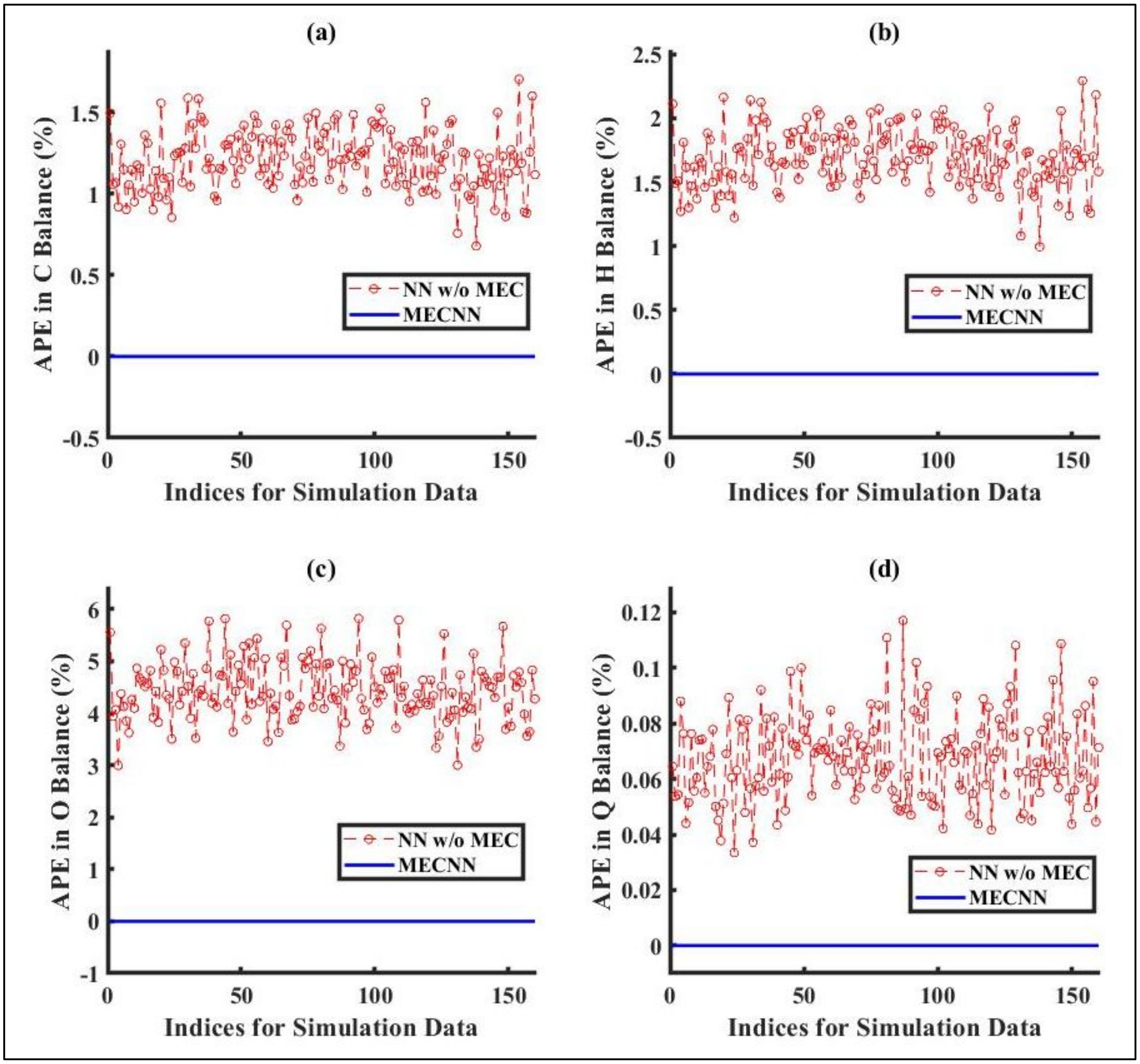

Fig. S31: Comparison between MECNN and NN w/o mass-energy constraints in terms of violating (a) carbon (C), (b) hydrogen (H), (c) oxygen (O) and (d) energy (Q) balance constraints during simulation (noise in the measurement data represented by Eq. (5))

C.3. Case Study 3: Electrically Heated Steam-Methane Reforming Reactor

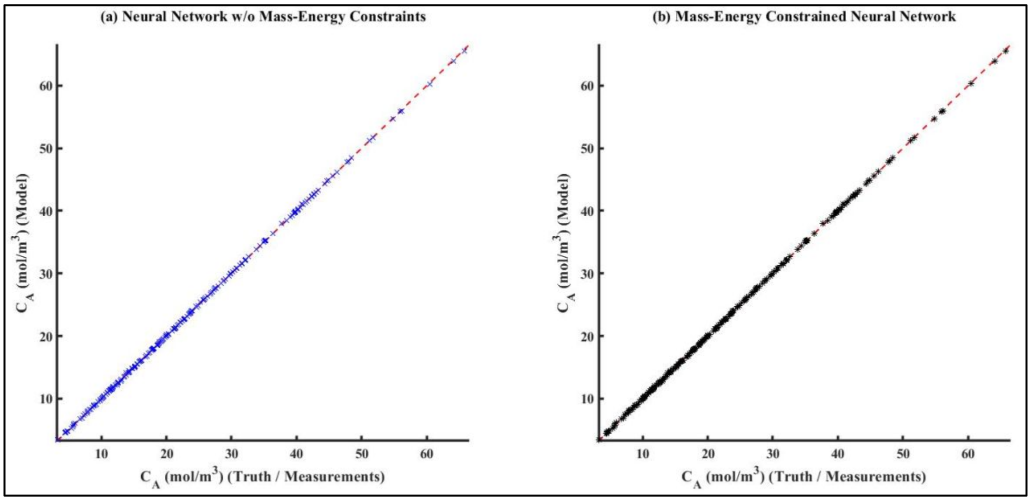

Fig. S32: Comparison of results between MECNN and NN w/o mass-energy constraints for the training data of C_A at system boundary (noise in the measurement data represented by Eq. (3))

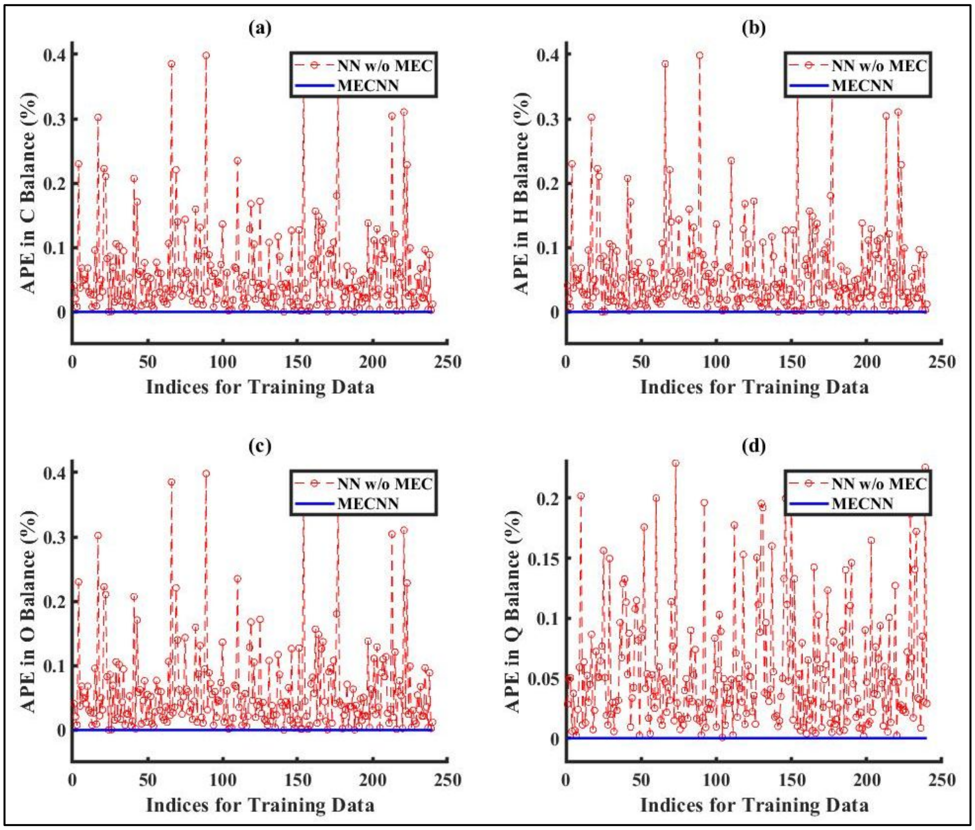

Fig. S33: Comparison between MECNN and NN w/o mass-energy constraints in terms of violating (a) carbon (C), (b) hydrogen (H), (c) oxygen (O) and (d) energy (Q) balance constraints at system boundary during training (noise in the measurement data represented by Eq. (3))

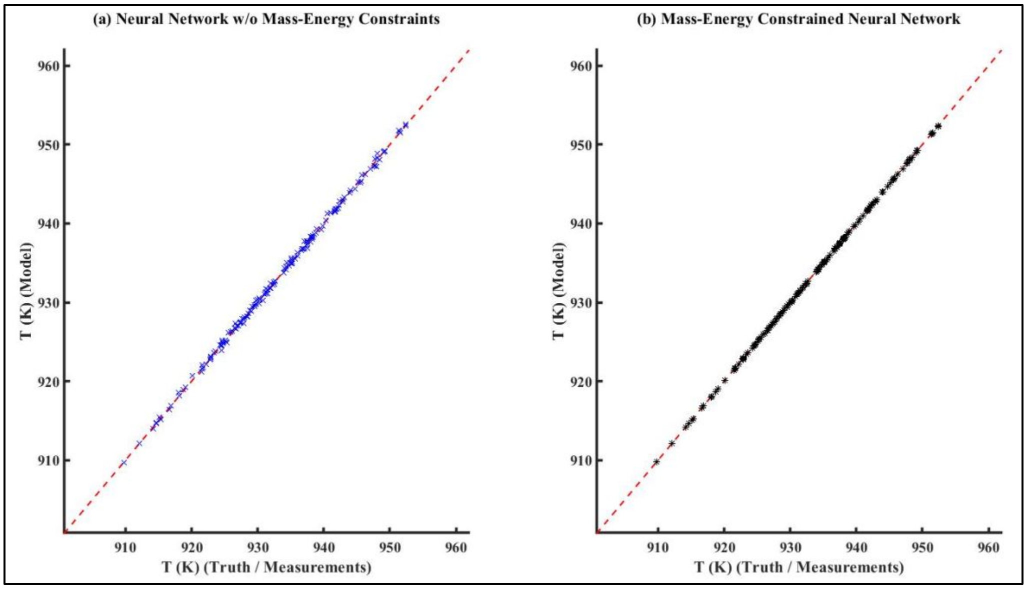

Fig. S34: Comparison of results between MECNN and NN w/o mass-energy constraints for the simulation data of T at system boundary (noise in the measurement data represented by Eq. (3))

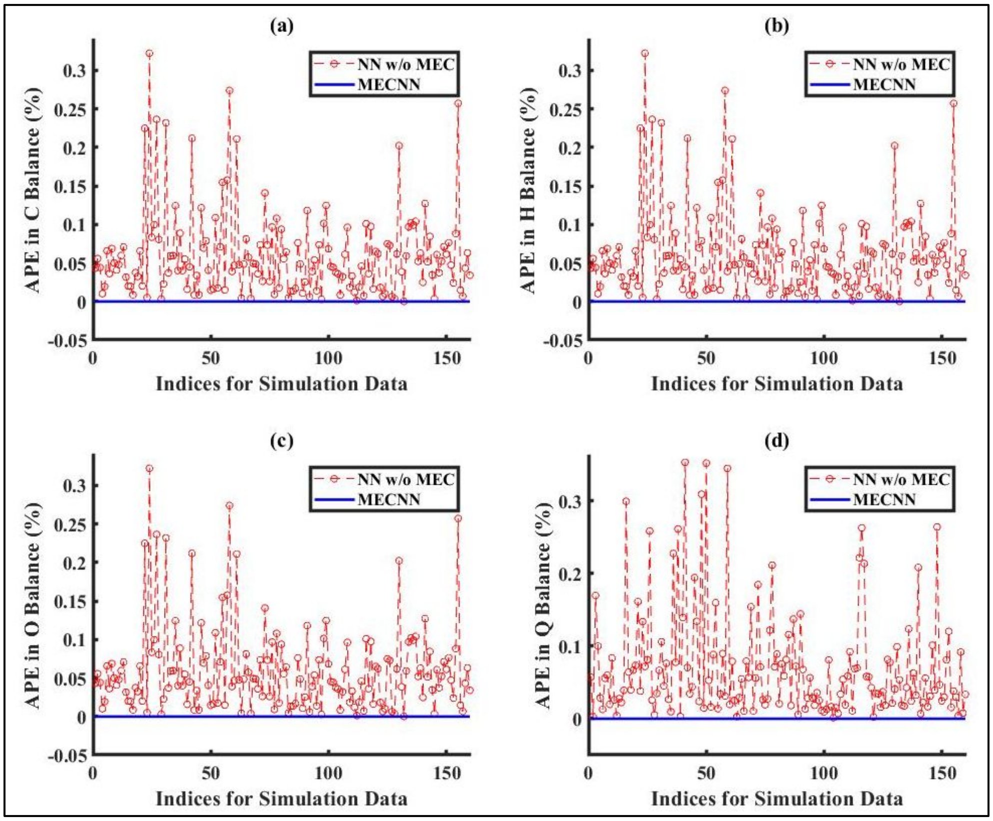

Fig. S35: Comparison between MECNN and NN w/o mass-energy constraints in terms of violating (a) carbon (C), (b) hydrogen (H), (c) oxygen (O) and (d) energy (Q) balance constraints at system boundary during simulation (noise in the measurement data represented by Eq. (3))

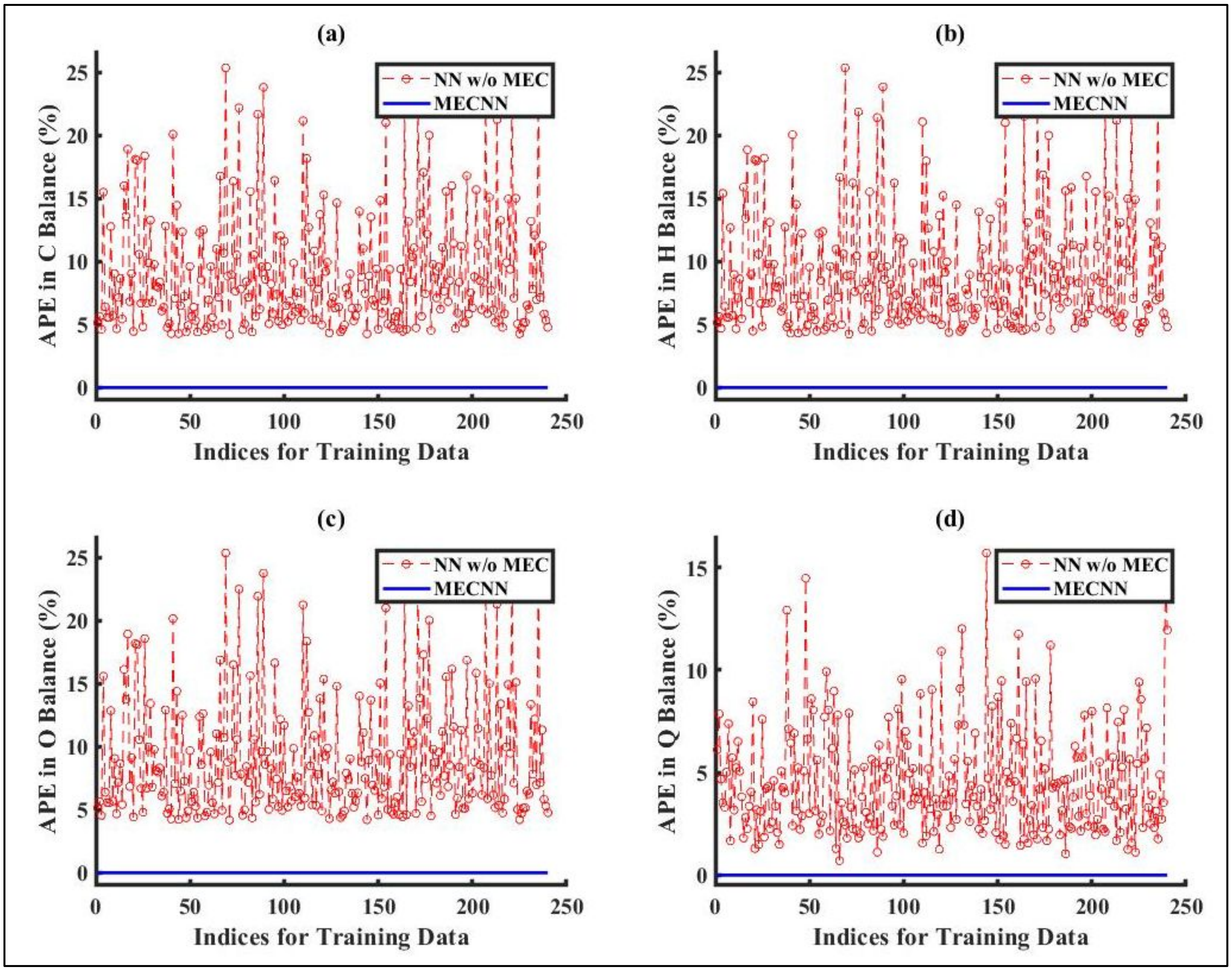

Fig. S36: Comparison between MECNN and NN w/o mass-energy constraints in terms of violating (a) carbon (C), (b) hydrogen (H), (c) oxygen (O) and (d) energy (Q) balance constraints at boundary of first discretization element during training (noise in the measurement data represented by Eq. (4))

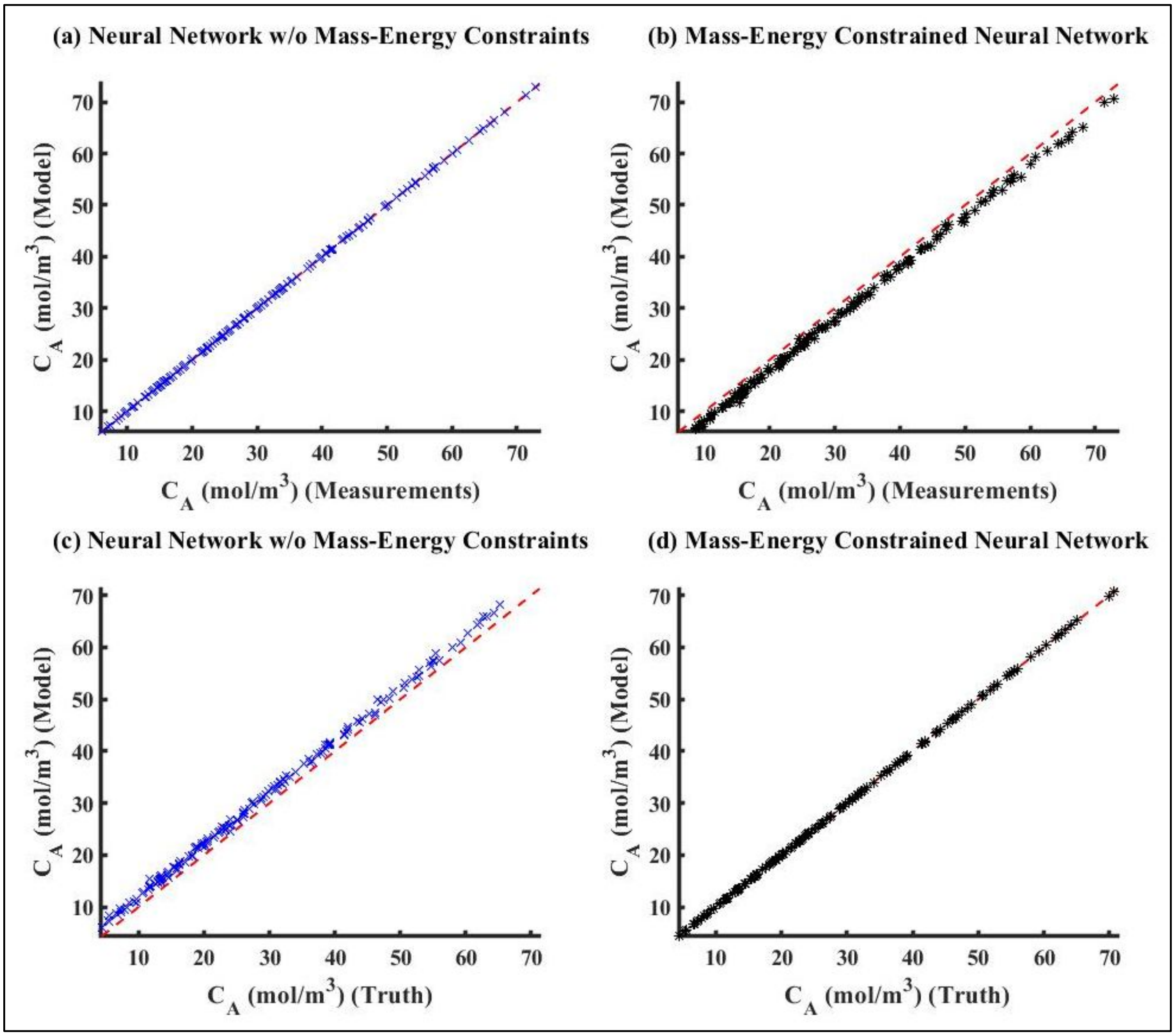

Fig. S37: Comparison of results between MECNN and NN w/o mass-energy constraints for the simulation data of C_A at boundary of first discretization element (noise in the measurement data represented by Eq. (4))

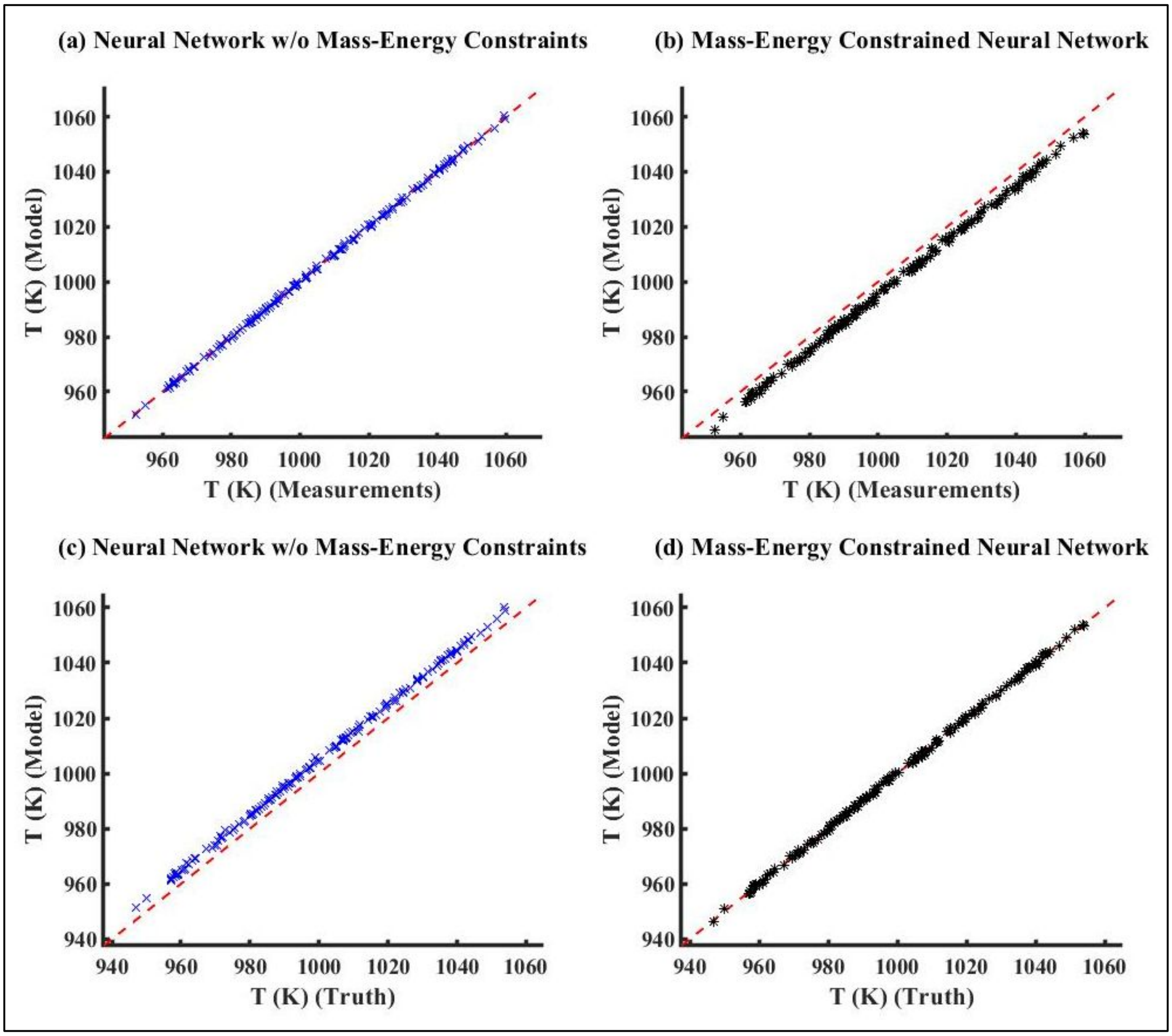

Fig. S38: Comparison of results between MECNN and NN w/o mass-energy constraints for the simulation data of T at boundary of first discretization element (noise in the measurement data represented by Eq. (4))

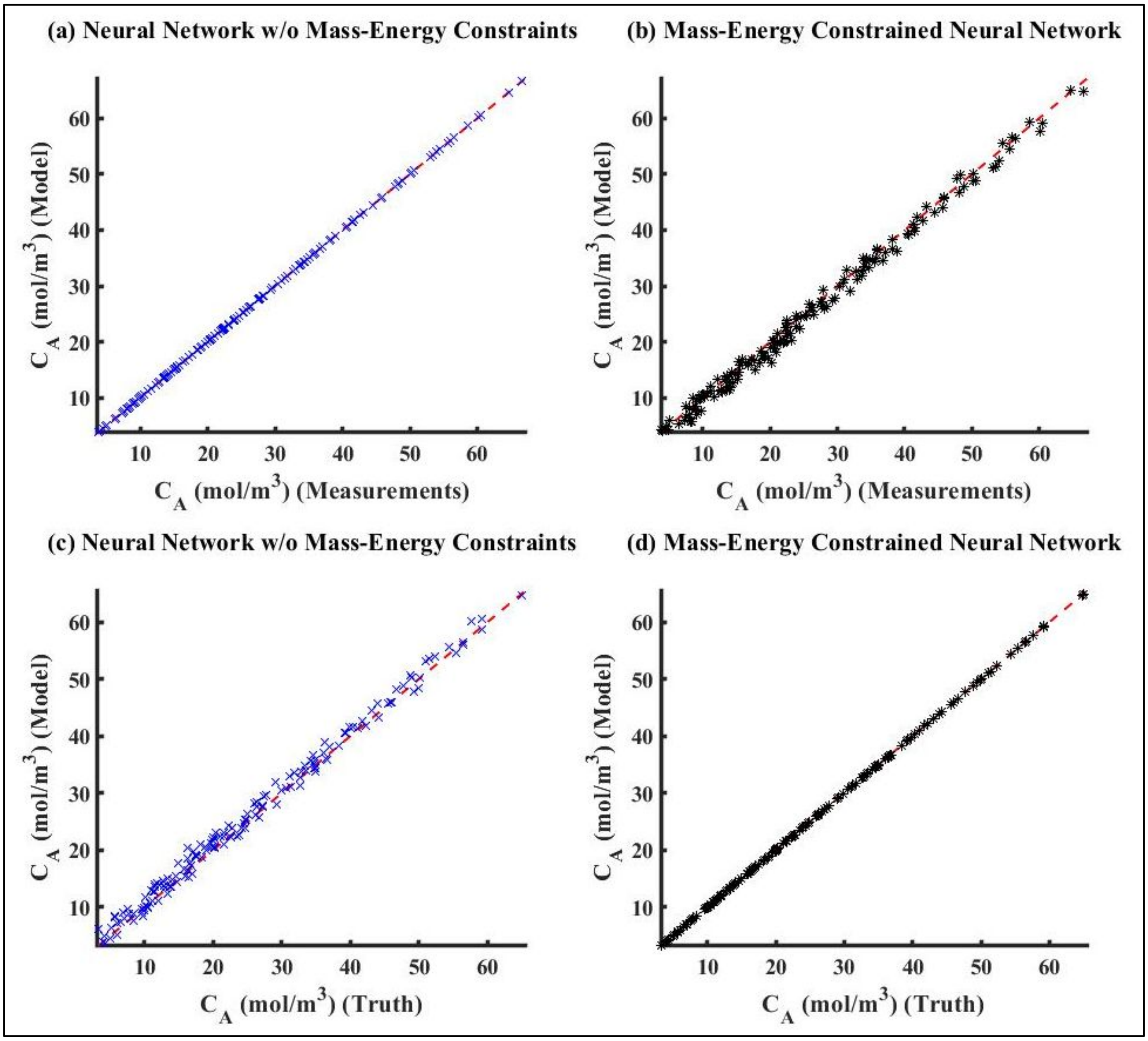

Fig. S39: Comparison of results between MECNN and NN w/o mass-energy constraints for the simulation data of C_A at system boundary (noise in the measurement data represented by Eq. (5))

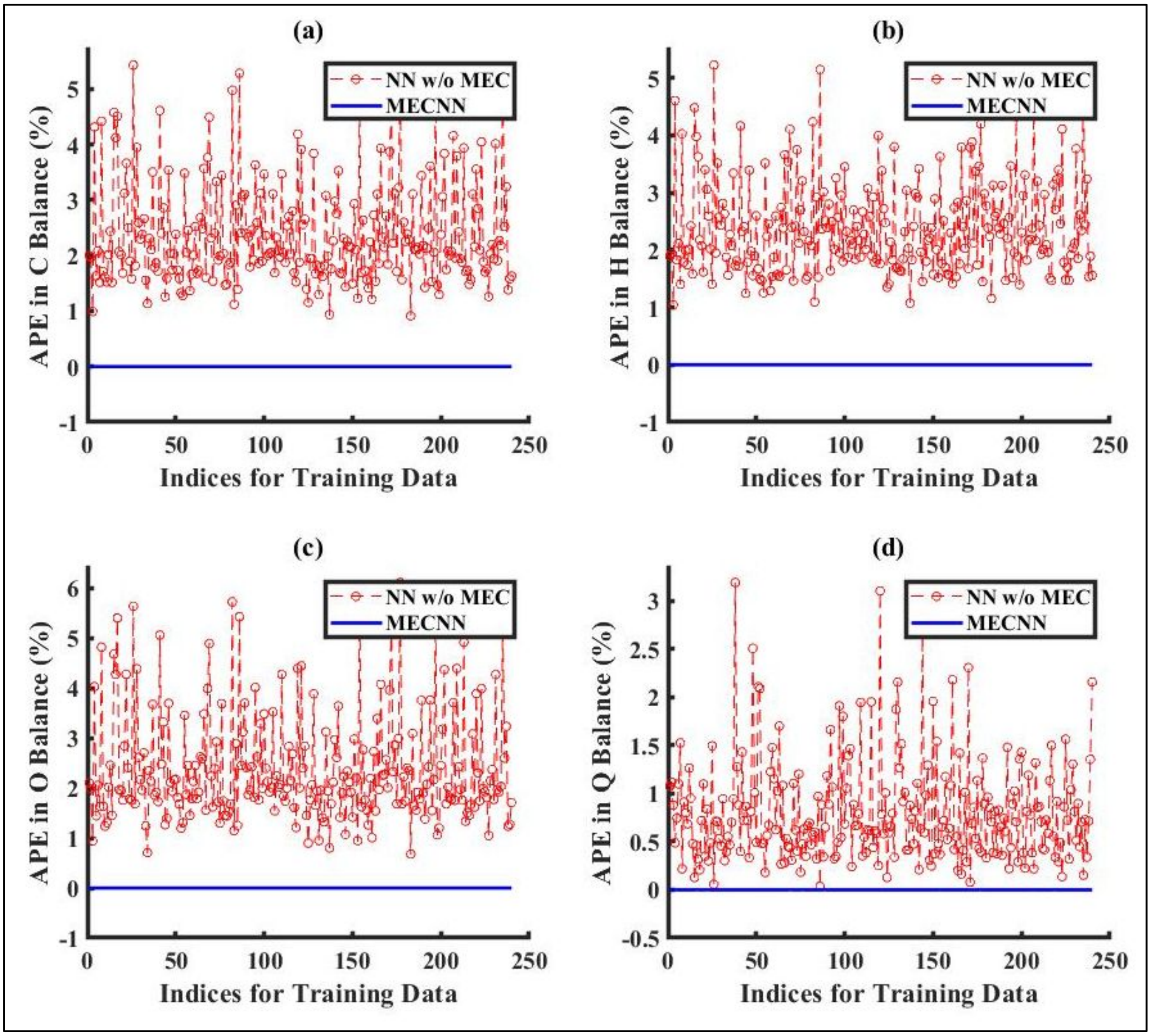

Fig. S40: Comparison between MECNN and NN w/o mass-energy constraints in terms of violating (a) carbon (C), (b) hydrogen (H), (c) oxygen (O) and (d) energy (Q) balance constraints at system boundary during training (noise in the measurement data represented by Eq. (5))

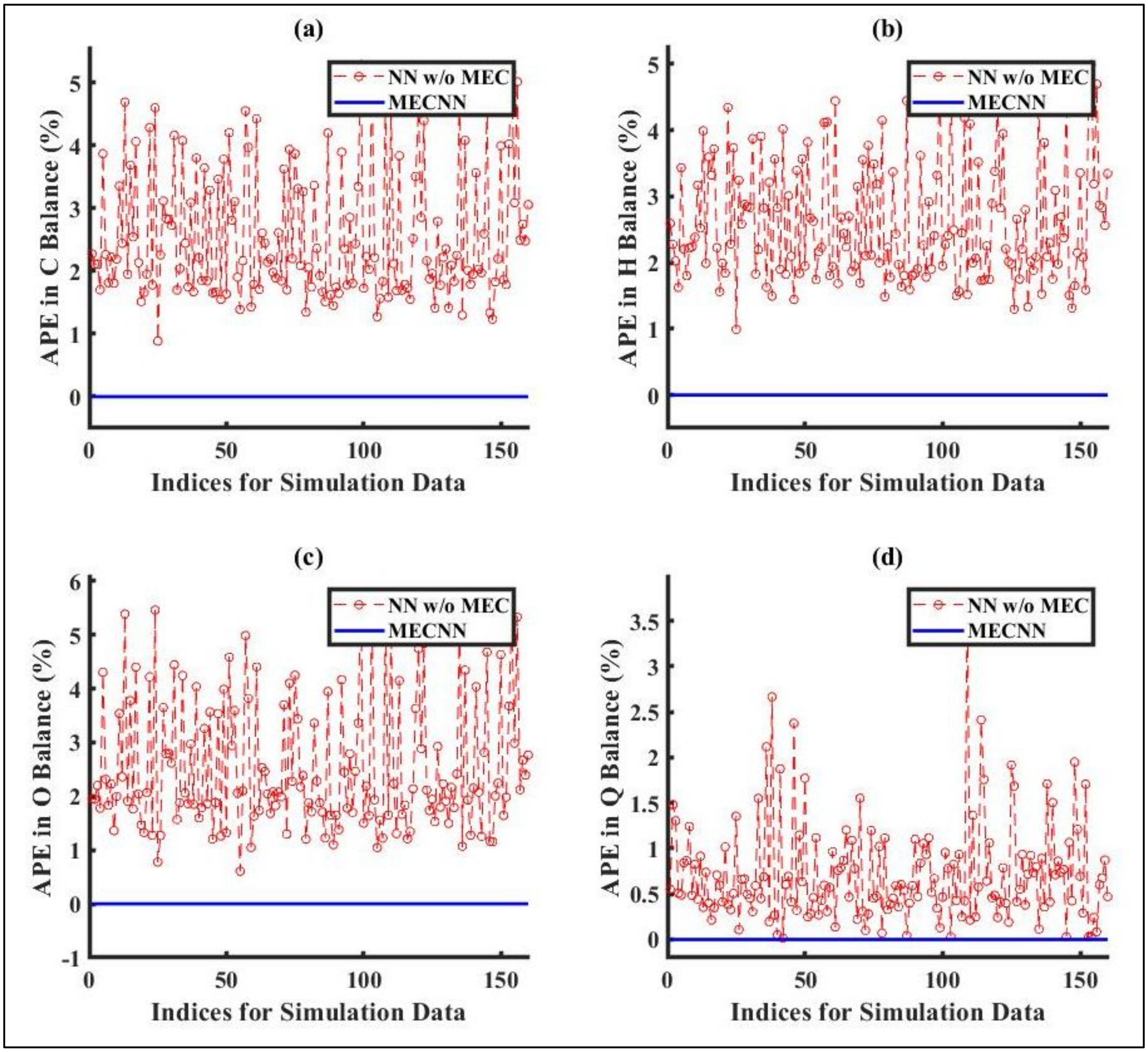

Fig. S41: Comparison between MECNN and NN w/o mass-energy constraints in terms of violating (a) carbon (C), (b) hydrogen (H), (c) oxygen (O) and (d) energy (Q) balance constraints at system boundary during simulation (noise in the measurement data represented by Eq. (5))

S.4: Additional Training / Simulation Results for Dynamic MECNNs

This section consists of additional training and simulation (forward problem) results obtained from dynamic implementations of MECNNs while modeling the three case study examples under different types of error characterizations. The results corresponding to both lumped and distributed models of the superheater / reheater system have been collectively compiled under Case Study 1. In this section,

- **Figs. S42** through **S50** include additional dynamic results for Case Study 1. Out of these, **Figs. S42**, **S43**, and **S44** show results for the lumped parameter model of the superheater / reheater system, while the rest show results for the distributed data-driven model.
- **Figs. S51** through **S55** include additional dynamic results for Case Study 2.
- **Figs. S56** through **S59** include additional dynamic results for Case Study 3.

S.4.1. Case Study 1: Adiabatic Superheater / Reheater System

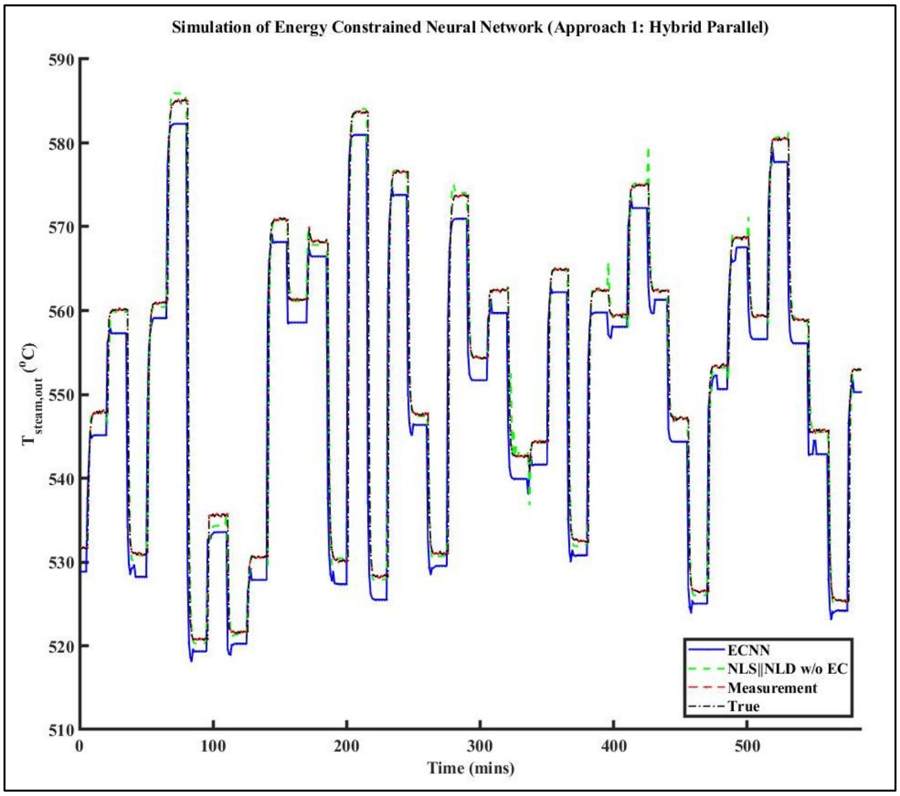

Fig. S42: Comparison of results between hybrid parallel ECNN and NLS || NLD w/o energy constraints for simulation data of $T_{St,out}$ (noise in the measurement data represented by Eq. (4))

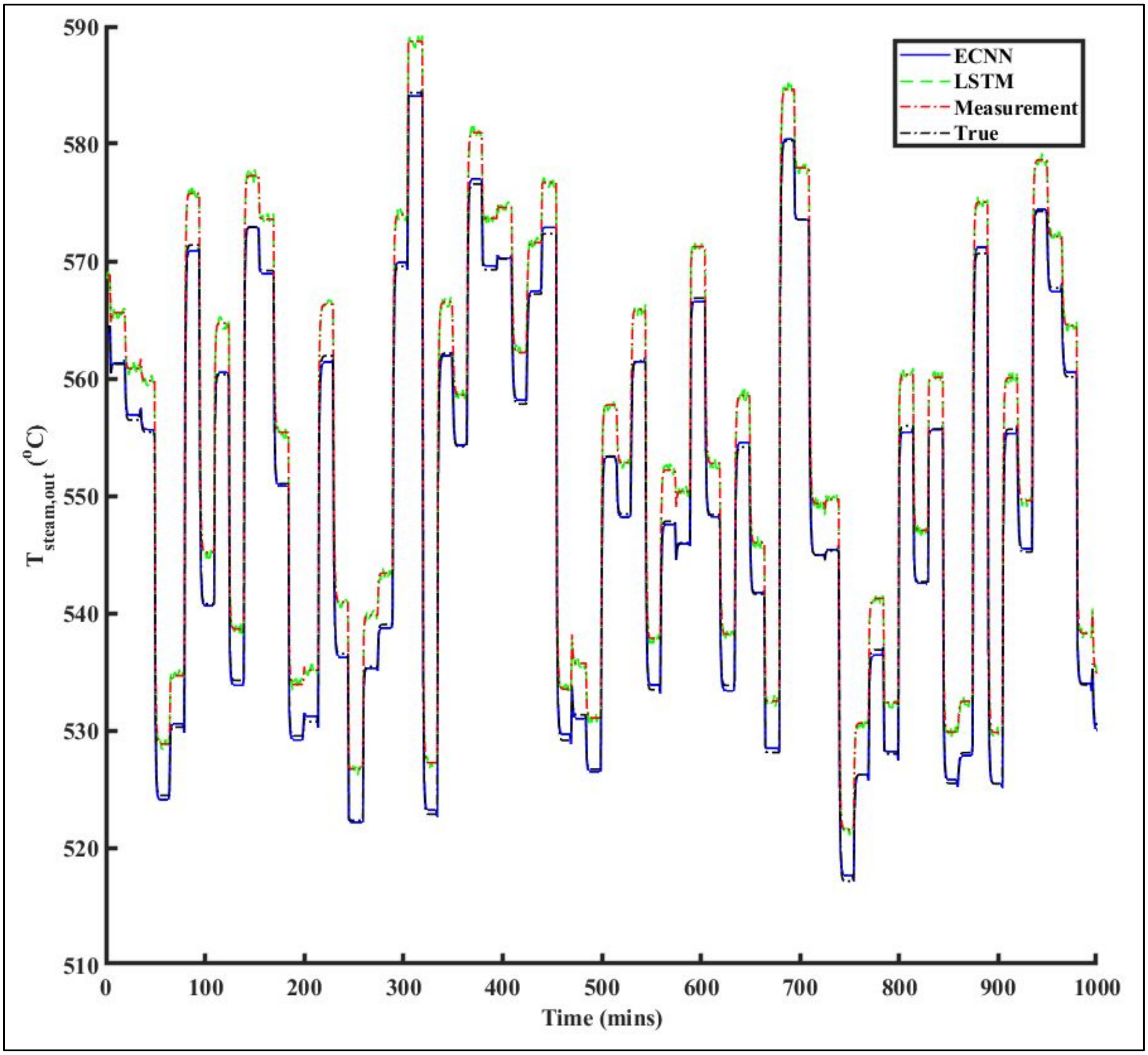

Fig. S43: Comparison of results between hybrid series (NLD – NLS) ECNN and LSTM-type RNN w/o energy constraints for simulation data of $T_{St,out}$ (noise in the measurement data represented by Eq. (4))

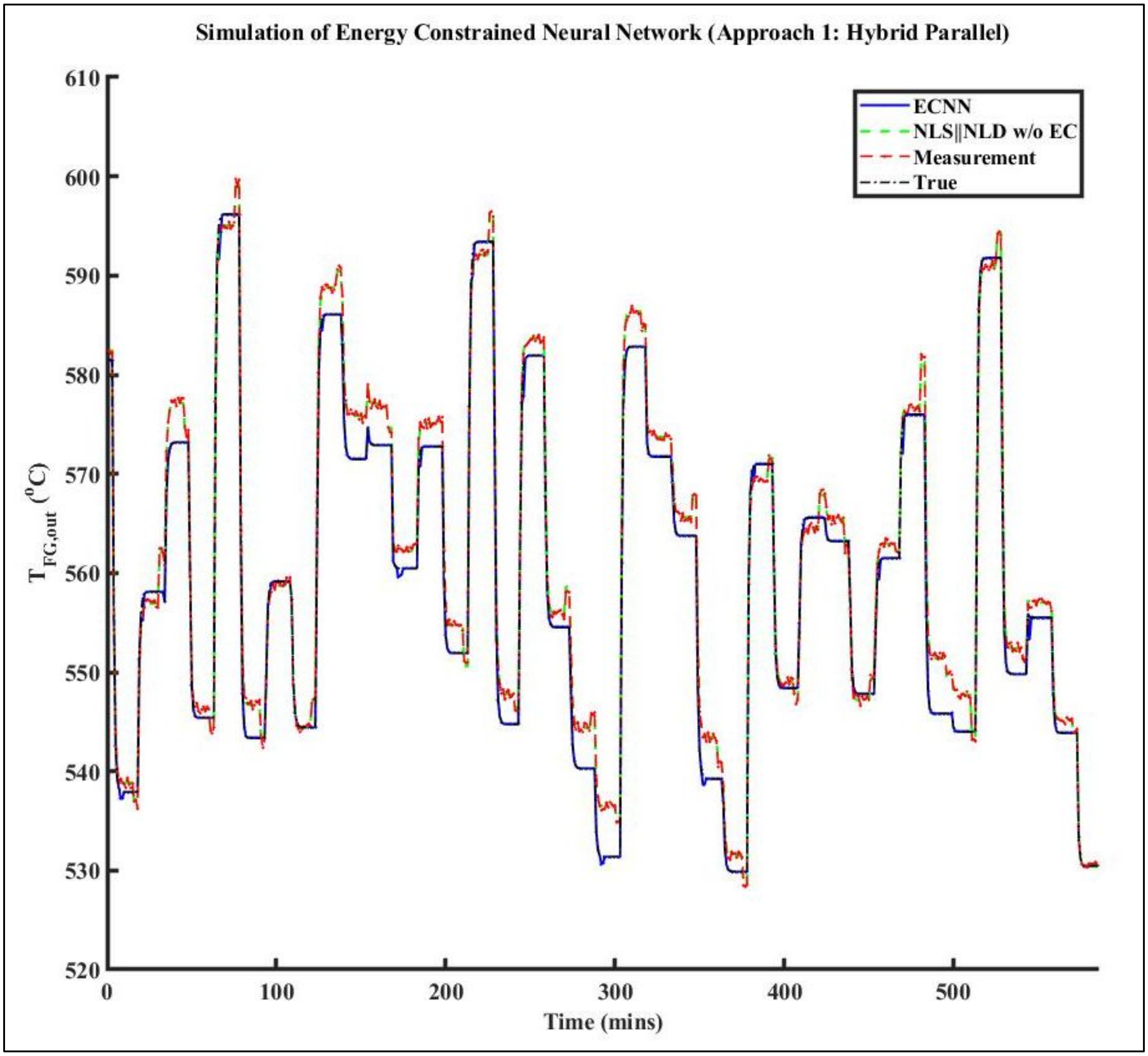

Fig. S44: Comparison of results between hybrid parallel ECNN and NLS || NLD model w/o energy constraints for simulation data of $T_{FG,out}$ (noise in the measurement data represented by Eq. (5))

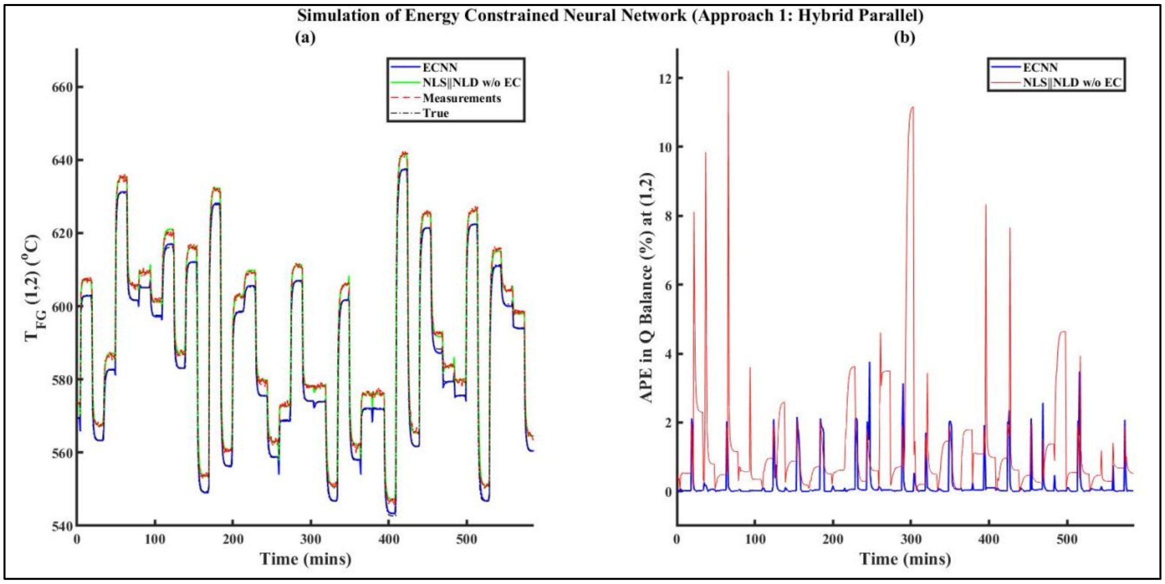

Fig. S45: Comparison of results between hybrid parallel ECNN and NLS || NLD w/o energy constraints (a) for simulation data of $T_{FG,out}$ and (b) in terms of violating energy (Q) balance constraints at grid (1,2) during simulation (noise in the measurement data represented by Eq. (4))

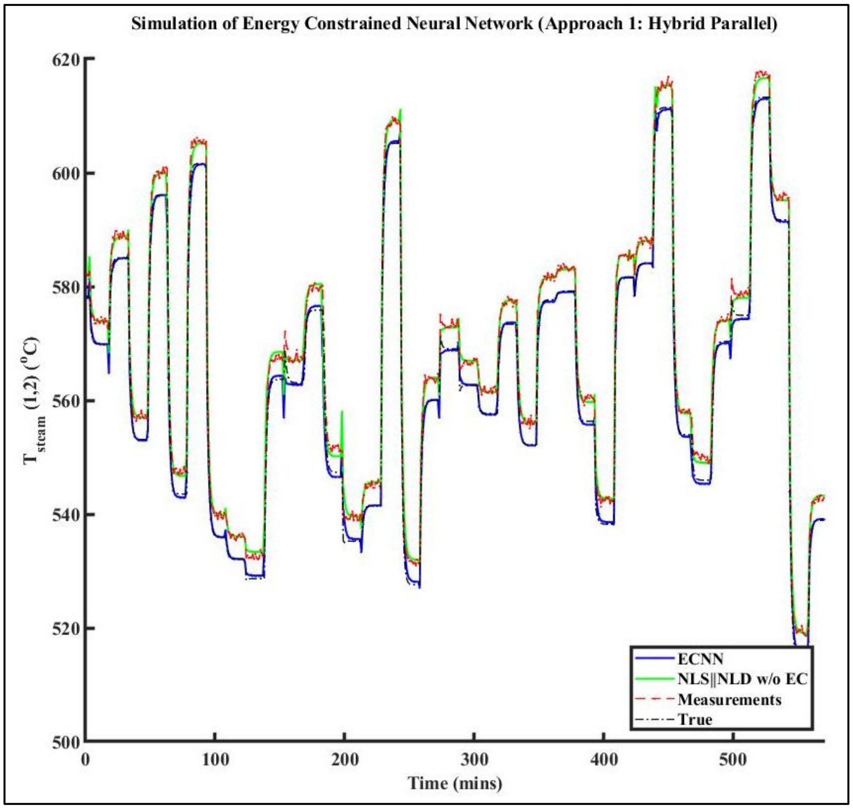

Fig. S46: Comparison of results between hybrid parallel ECNN and NLS || NLD w/o energy constraints for simulation data of $T_{St,out}$ (noise in the measurement data represented by Eq. (4))

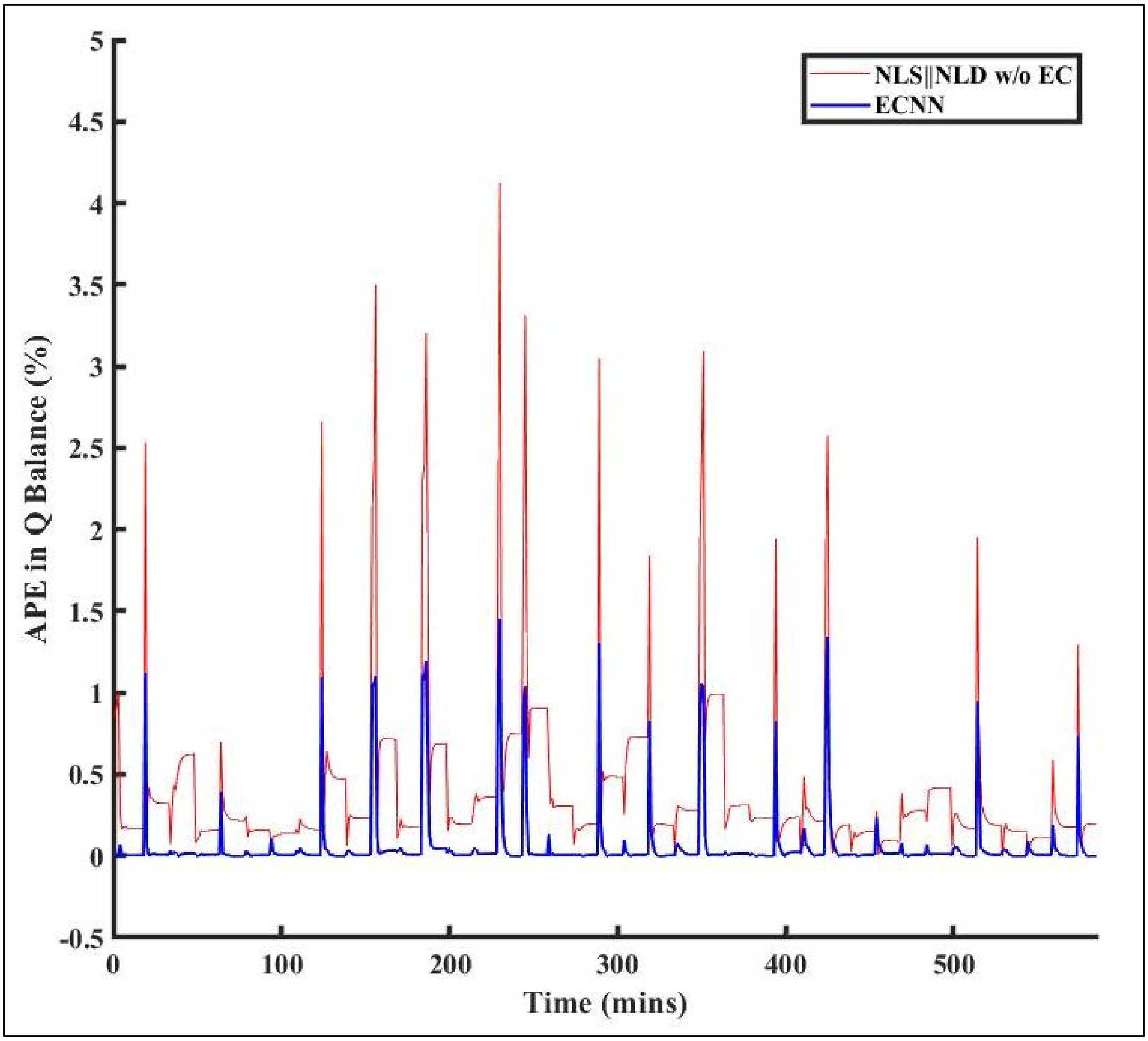

Fig. S47: Comparison of results between hybrid parallel ECNN and NLS || NLD w/o energy constraints in terms of violating energy (Q) balance constraints at system boundary during simulation (noise in the measurement data represented by Eq. (4))

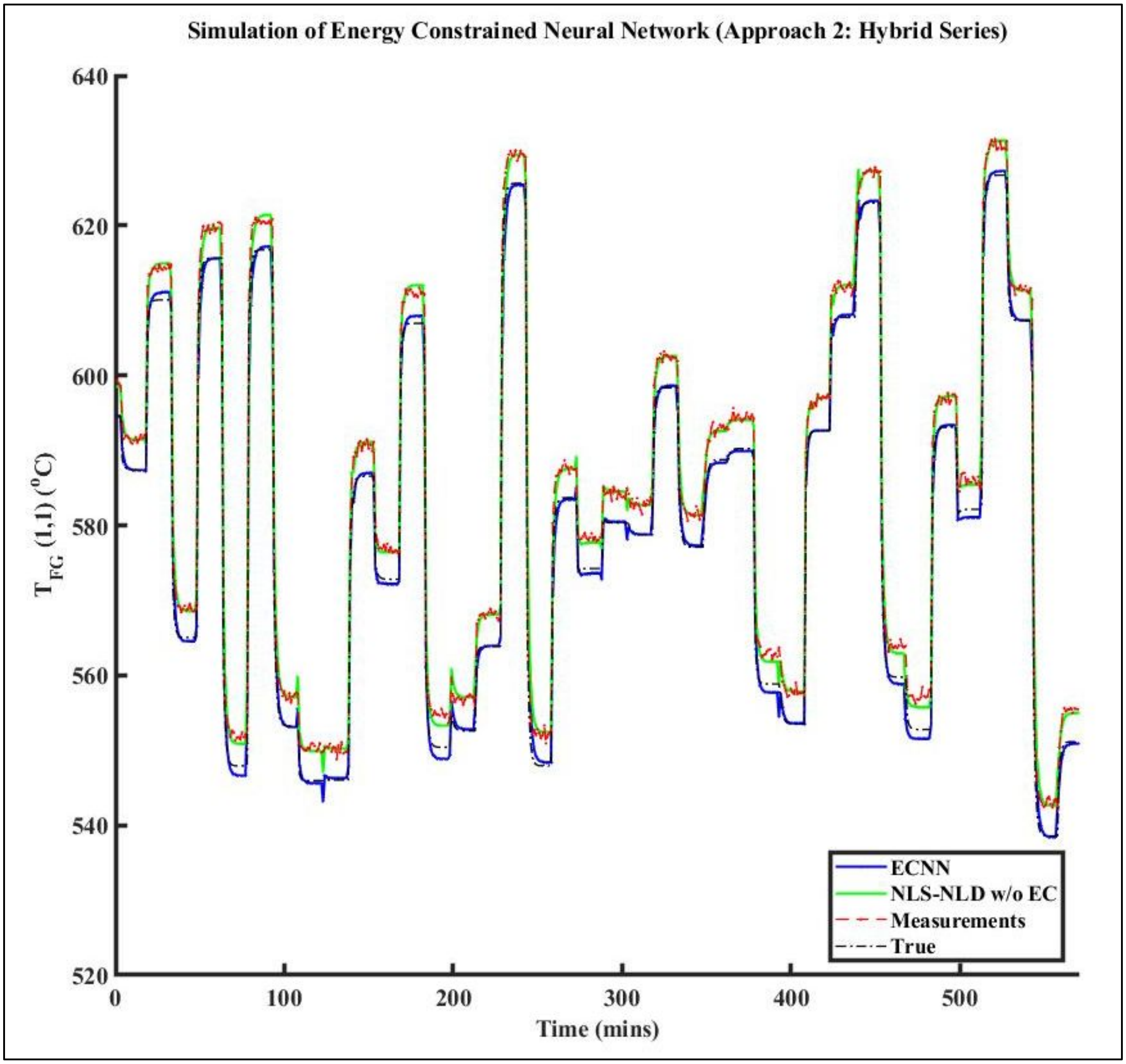

Fig. S48: Comparison of results between the hybrid series (NLS – NLD) ECNN and NLS – NLD w/o energy constraints for simulation data of $T_{FG,out}$ (noise in the measurement data represented by Eq. (4))

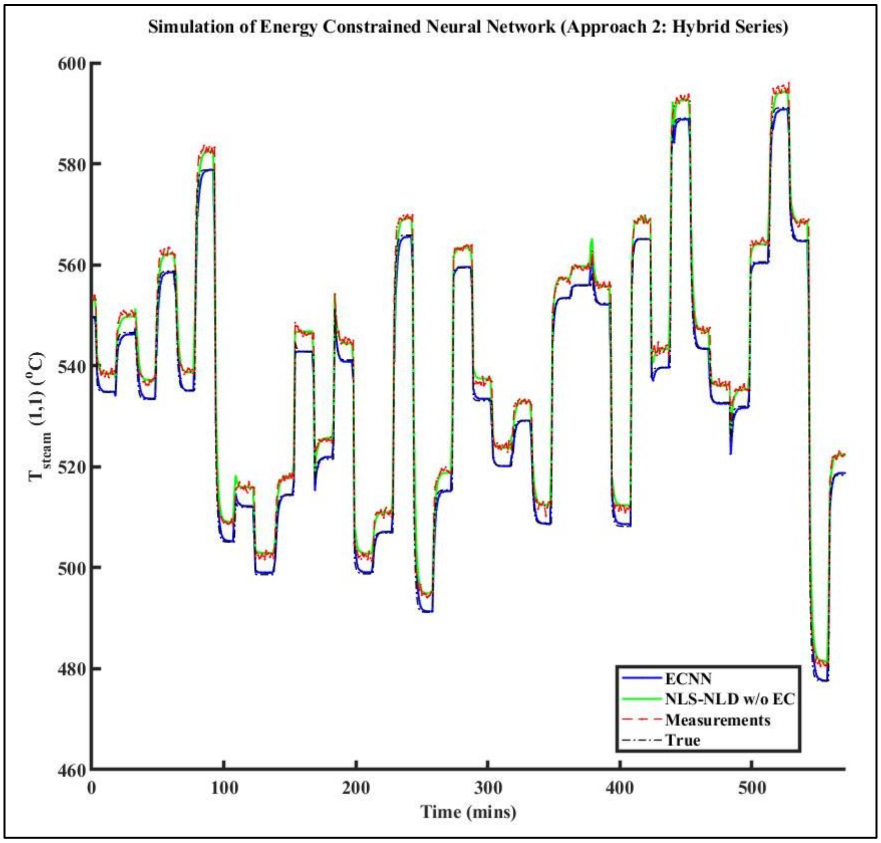

Fig. S49: Comparison of results between the hybrid series ECNN and NLS – NLD w/o energy constraints for simulation data of $T_{St,out}$ (noise in the measurement data represented by Eq. (4))

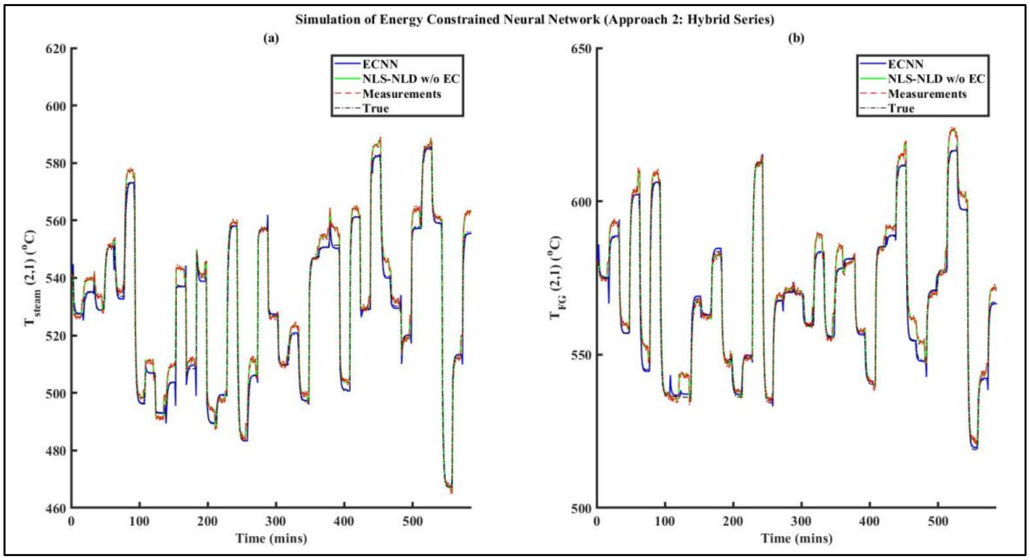

Fig. S50: Comparison of results between hybrid series (NLS – NLD) ECNN and NLS – NLD w/o energy constraints for simulation data of (a) $T_{St,out}$ and (b) $T_{FG,out}$ at grid $(2,1)$ (noise in the measurement data represented by Eq. (5))

S.4.2. Case Study 2: Non-Isothermal Van de Vusse Reactor System

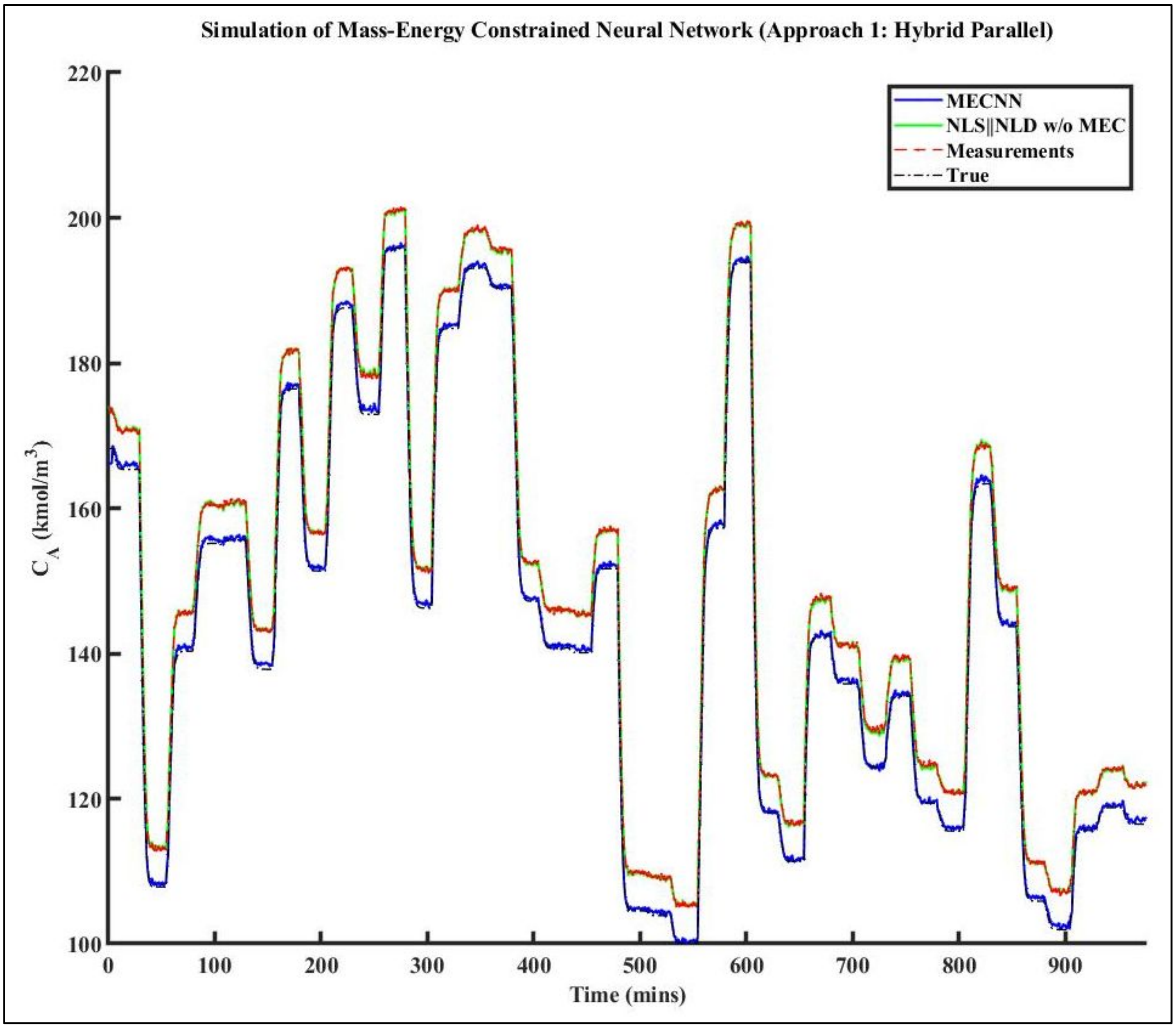

Fig. S51: Comparison of results between hybrid parallel MECNN and NLS || NLD w/o mass-energy constraints for simulation data of C_A (noise in the measurement data represented by Eq. (4))

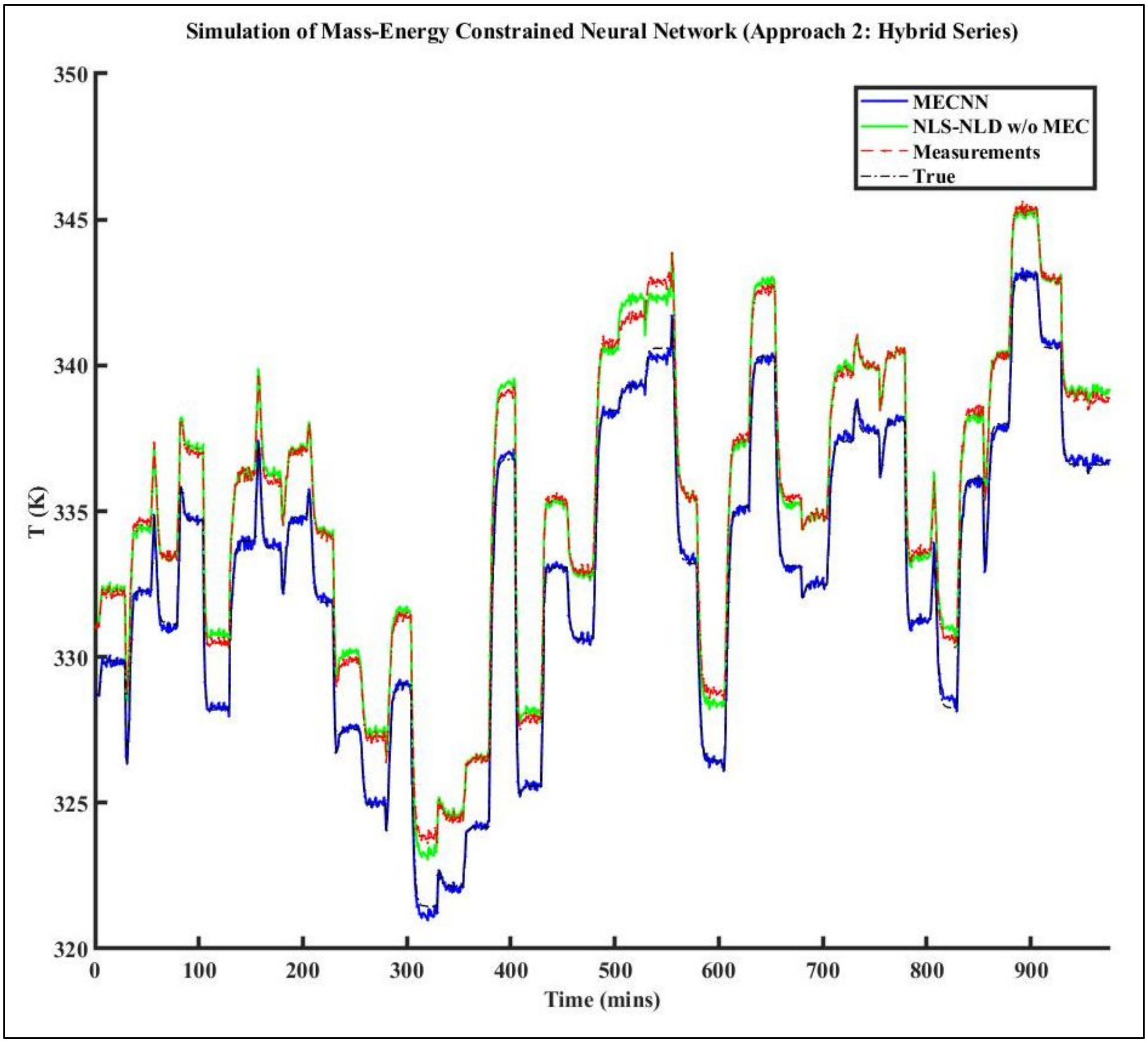

Fig. S52: Comparison of results between hybrid series MECNN and NLS – NLD w/o mass-energy constraints for simulation data of outlet reactor temperature (T) (noise in the measurement data represented by Eq. (4))

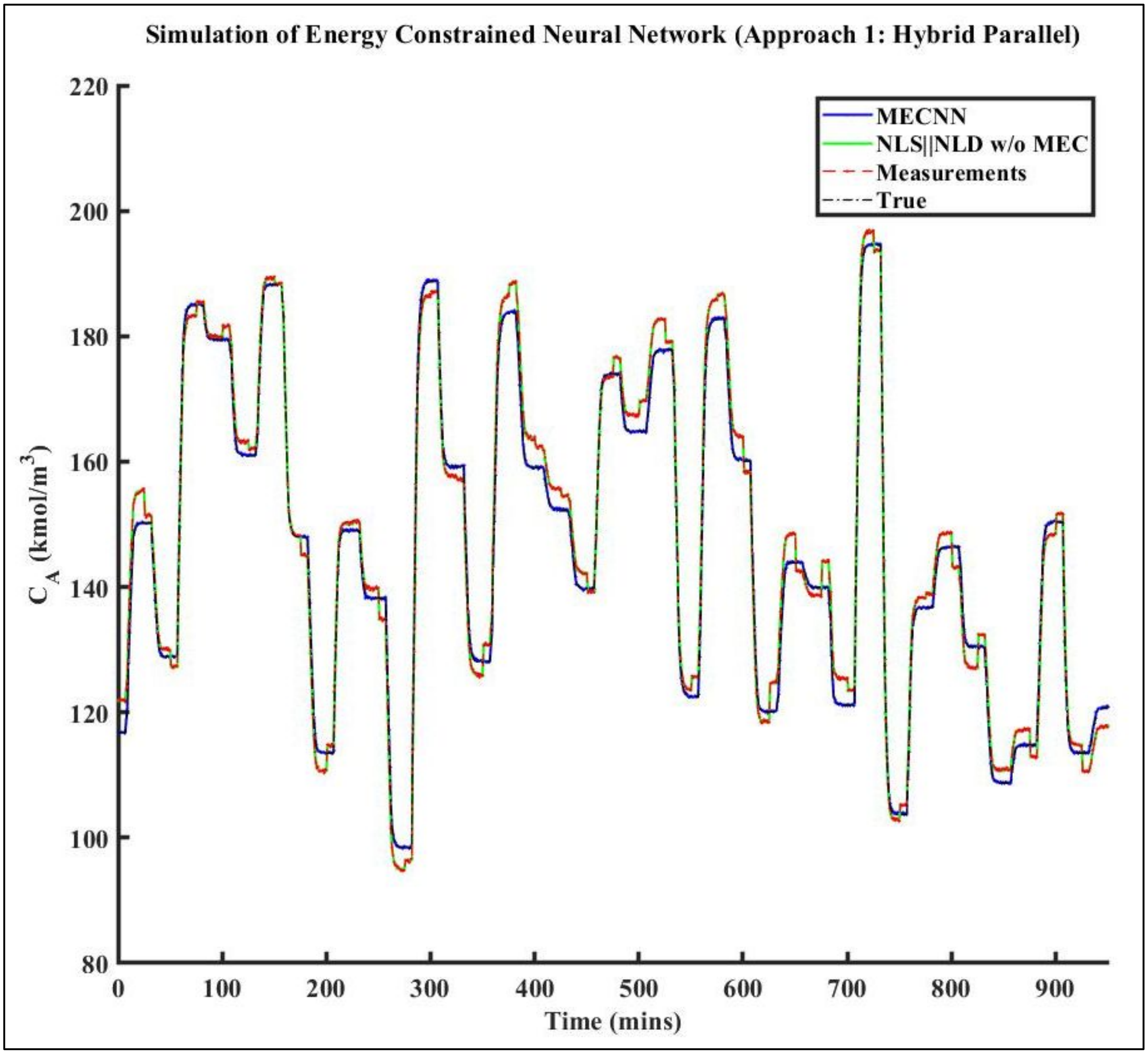

Fig. S53: Comparison of results between hybrid parallel ECNN and NLS || NLD w/o mass-energy constraints for simulation data of C_A (noise in the measurement data represented by Eq. (5))

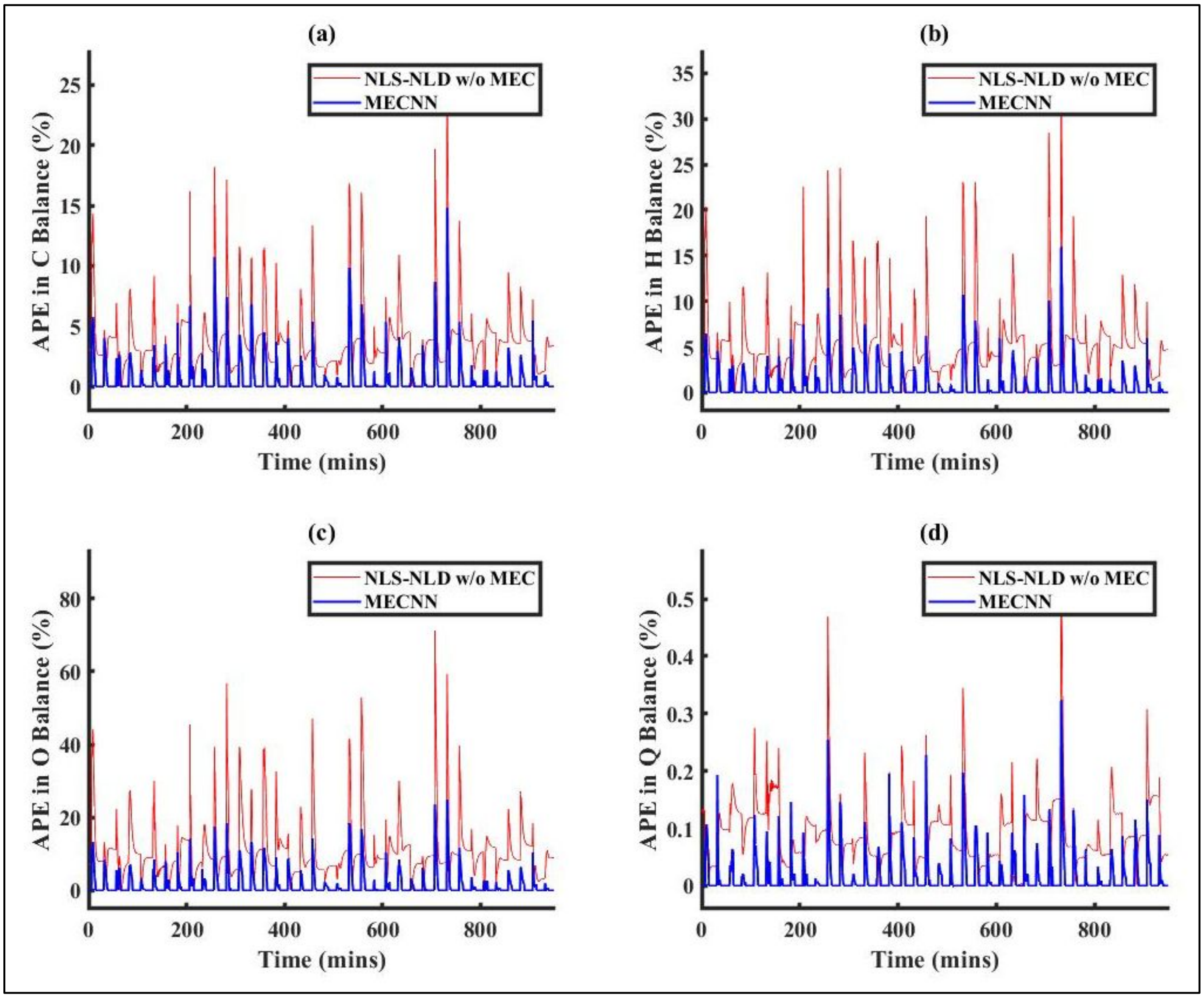

Fig. S54: Comparison between hybrid series MECNN and NLS – NLD model w/o mass-energy constraints in terms of violating (a) carbon (C), (b) hydrogen (H), (c) oxygen (O) and (d) energy (Q) balance constraints during simulation (noise in the measurement data represented by Eq. (5))

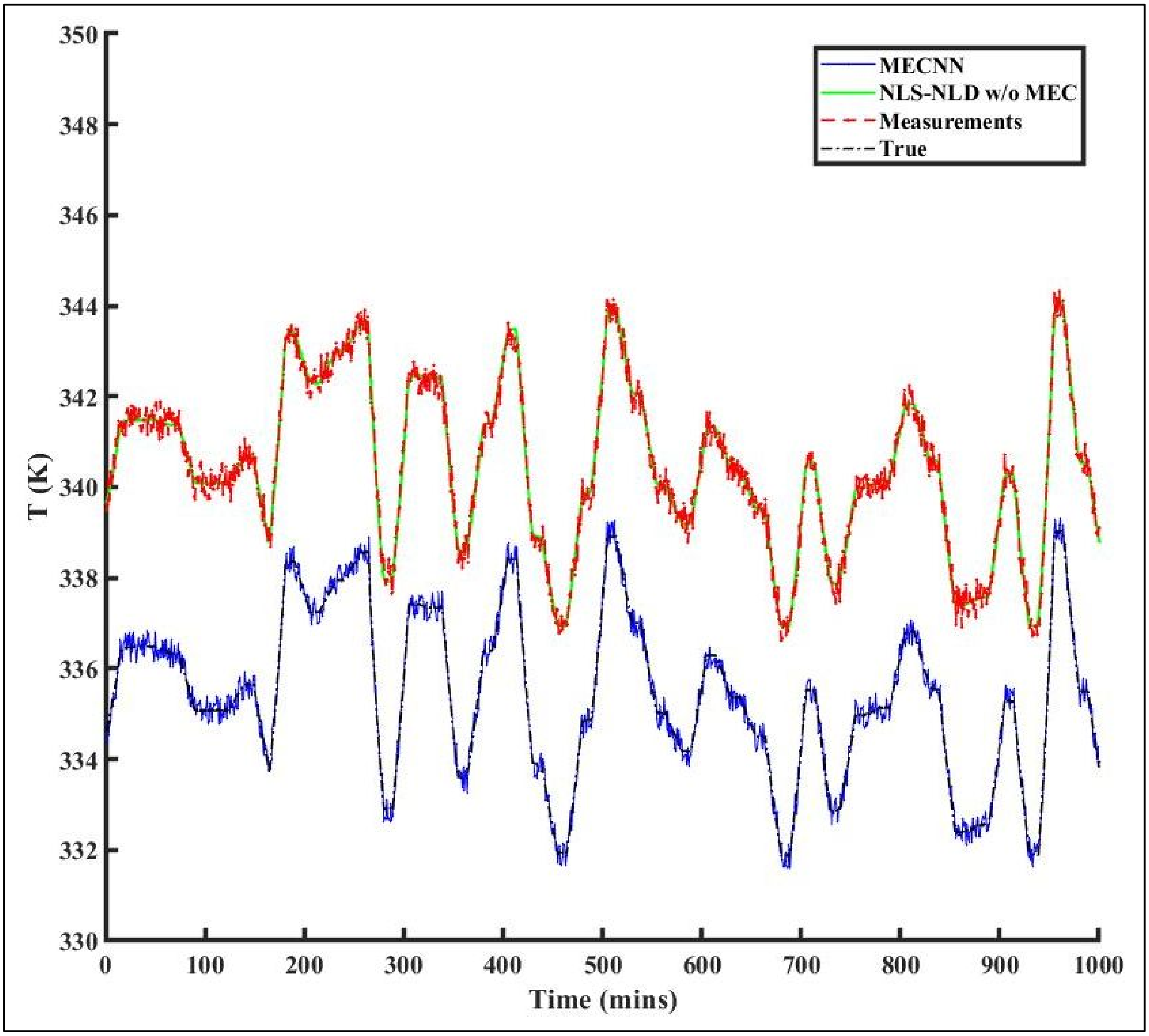

Fig. S55: Comparison of results between hybrid series (NLS – NLD) MECNN and unconstrained NLS – NLD for simulation data of T when holdup information is available (noise in the measurement data represented by Eq. (4))

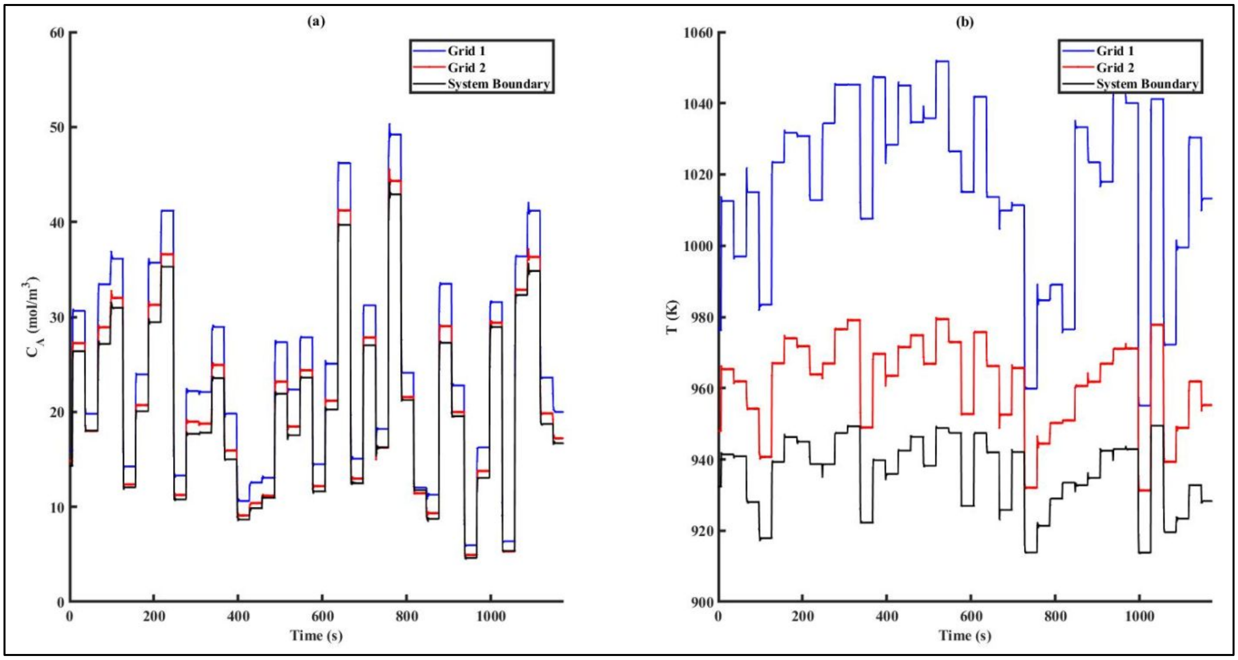

Fig. S56: Results from hybrid series (NLS – NLD) MECNN for distributions of (a) C_A and (b) T in axial coordinates during simulation (noise in measurement data represented by Eq. (4))

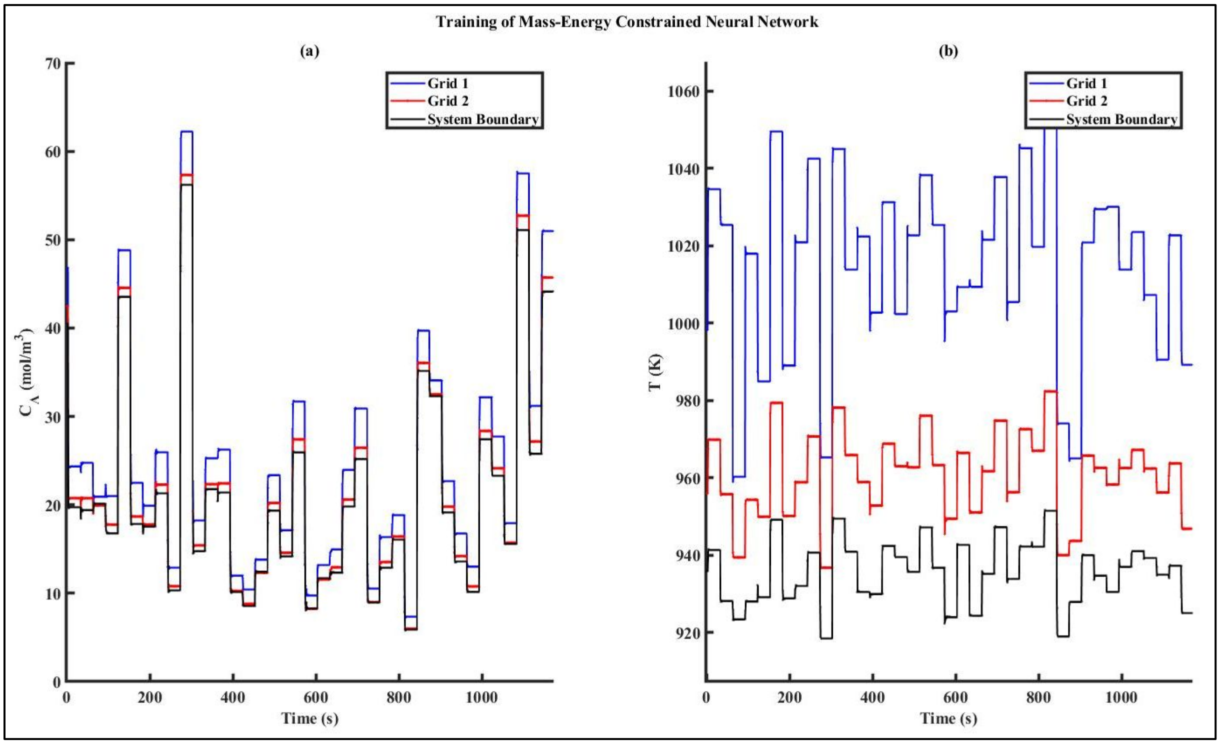

Fig. S57: Results from hybrid parallel (NLS || NLD) MECNN for distributions of (a) C_A and (b) T in axial coordinates during simulation (noise in measurement data represented by Eq. (5))

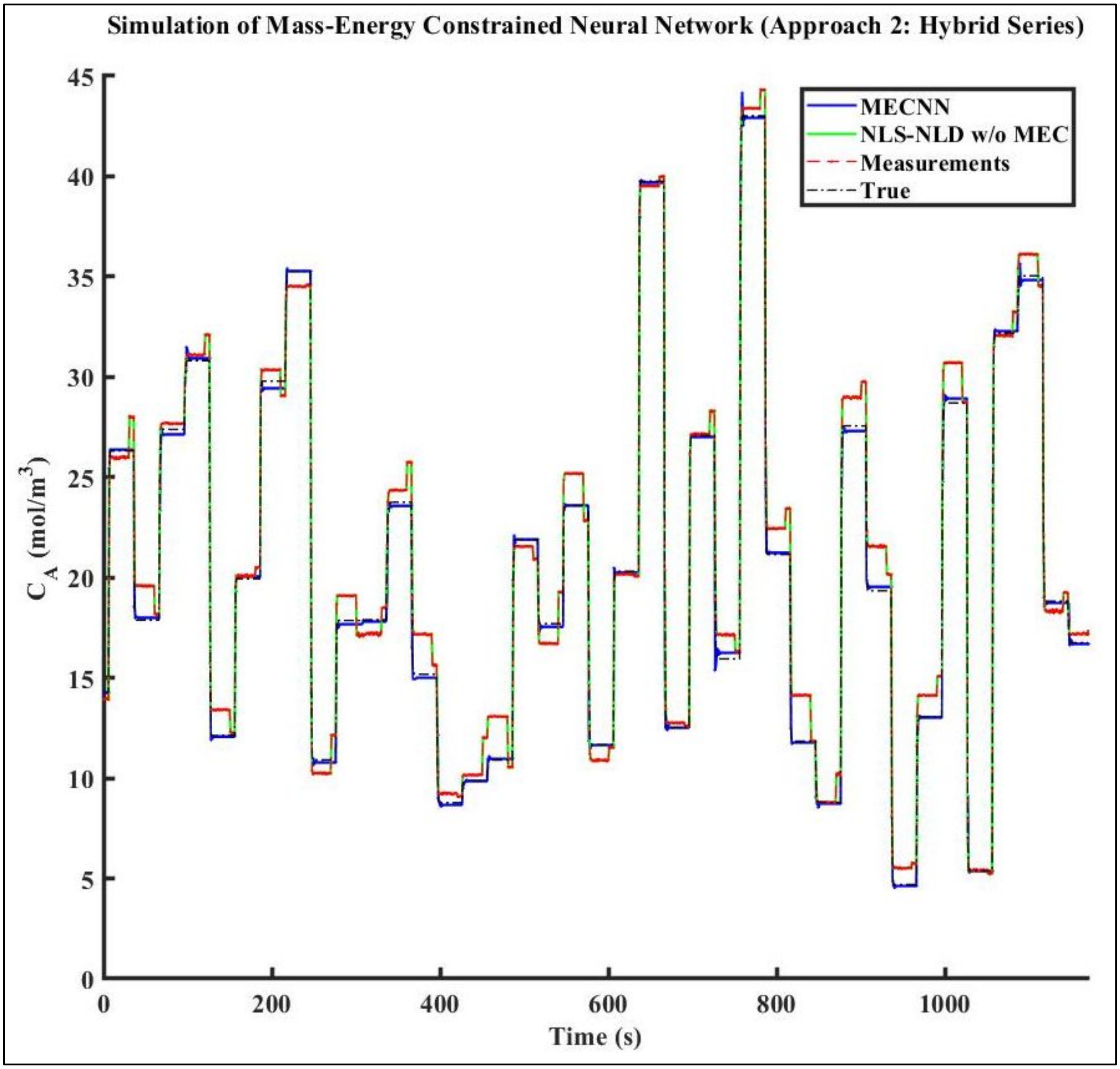

Fig. S58: Comparison of results between hybrid series (NLS – NLD) MECNN and NLS – NLD w/o mass-energy constraints for simulation data of C_A at system boundary (noise in the measurement data represented by Eq. (5))

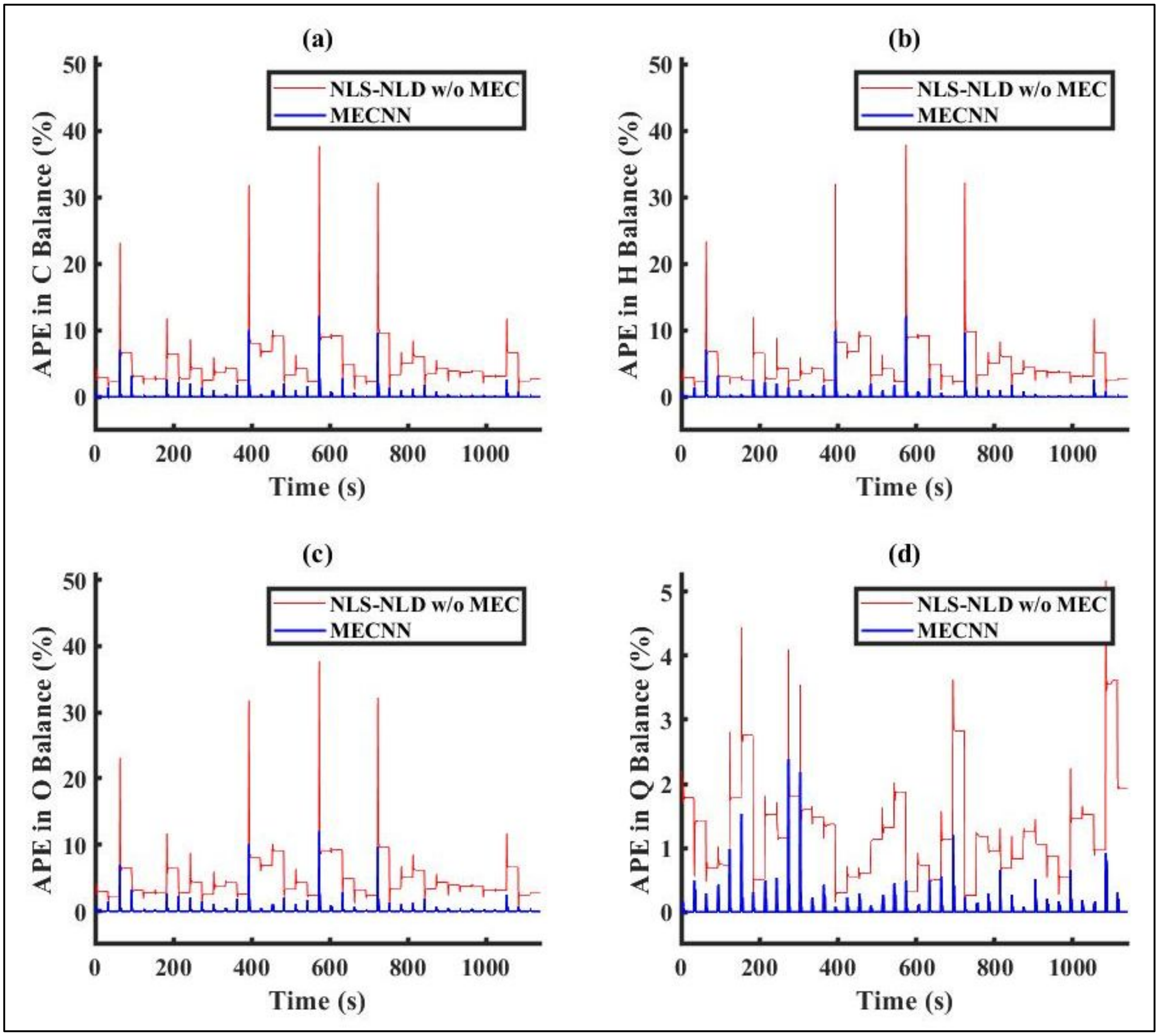

Fig. S59: Comparison between hybrid series (NLS – NLD) MECNN and NLS – NLD model w/o mass-energy constraints in terms of violating (a) C, (b) H, (c) O and (d) energy balance constraints at system boundary during simulation (noise in measurement data represented by Eq. (5))
